# Supplementary material for: Metal-organic framework nanocrystal-derived hollow porous materials: Synthetic strategies and emerging applications
Source: Innovation (Camb). 2022 Jul 6;3(5):100281. doi: 10.1016/j.xinn.2022.100281 (PMC9307687; doi:10.1016/j.xinn.2022.100281)
Supplement: Document S1. Figures S1–S5 and Tables S1–S4 [file mmc1.pdf]

The Innovation, Volume *3*

Supplemental Information

Metal-organic framework nanocrystal-derived hollow porous materials: Synthetic strategies and emerging applications

Xiaolu Liu, Gaurav Verma, Zhongshan Chen, Baowei Hu, Qifei Huang, Hui Yang, Shengqian Ma, and Xiangke Wang

Supplemental Information on

Metal-Organic Framework Nanocrystals Derived Hollow Porous Materials: Synthetic Strategies and Emerging Applications

Xiaolu Liu[a,b], Gaurav Verma[c], Zhongshan Chen[a], Baowei Hu[b], Qifei Huang[d], Hui Yang,[a,*] Shengqian Ma[c,*] Xiangke Wang,[a,b*]

[a] College of Environmental Science and Engineering, North China Electric Power University, Beijing, 102206, P. R. China. E-mail: h.yang@ncepu.edu.cn; xkwang@ncepu.edu.cn

[b] School of Life Science, Shaoxing University, Huancheng West Road 508, Shaoxing 312000, P.R. China

[c] Department of Chemistry, University of North Texas, 1508 W Mulberry St, Denton, TX 76201, USA. E-mail: shengqian.ma@unt.edu

[d] State Key Laboratory of Environmental Criteria and Risk Assessment, Chinese Research Academy of Environmental Sciences, Beijing 100012, P.R. China

Contents:

Supplementary Figures: 5
Supplementary Tables: 4
References for SI reference citations

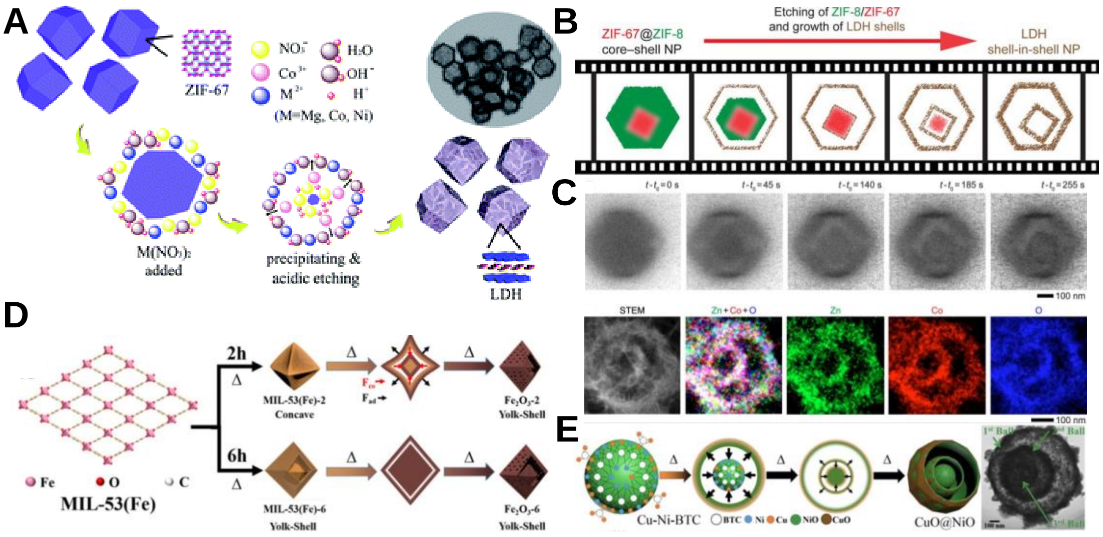

Figure S1. (A) The formation illustration of LDH nanocages by simultaneous precipitation and acidic etching.[1] (B) Schematic illustration depicting the conversion process of ZIF-67@ZIF-8 core-shell NPs into "shell-in-shell" LDH nanocages. (C) (top) Time series of in situ liquid-phase TEM images showing the room-temperature conversion of a ZIF-67@ZIF-8 NP into a shell-in-shell LDH nanocage, (bottom) STEM image and the corresponding EDX maps of the shell-in-shell LDH nanocage.[2] (D) The formation process of hollow Fe_2O_3 nanostructures with the octahedron and yolk-shell octahedron.[3] (E) Schematic illustration of strategies for using MOF nanocrystals as sacrificial templates and precursors for the synthesis of multilayer CuO@NiO hollow spheres.[4] Copyright Royal Society of Chemistry and American Chemical Society.

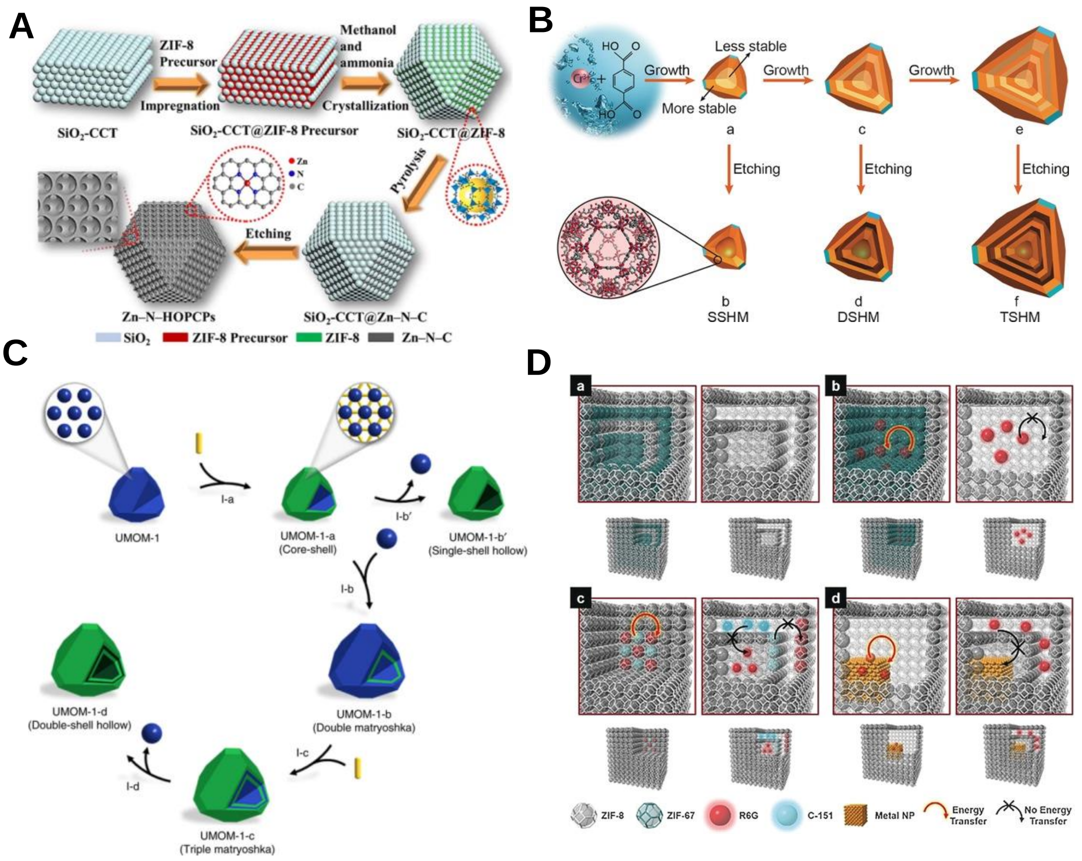

Figure S2. (A) Illustration of the fabrication of the Zn-N-HOPCPs.[5] (B) Fabrication of single-, double-, and triple-shelled hollow MIL-101.[6] (C) Schematic showing the synthetic process of single-crystal hollow MOFs with multishell via an etching-epitaxial growth strategy.[7] (D) Fabrication of multishelled hollow ZIF-8 by using multilayered ZIF-67@ZIF-8 microcrystals as templates.[8] Copyright Elsevier, John Wiley and Sons, and Springer Nature.

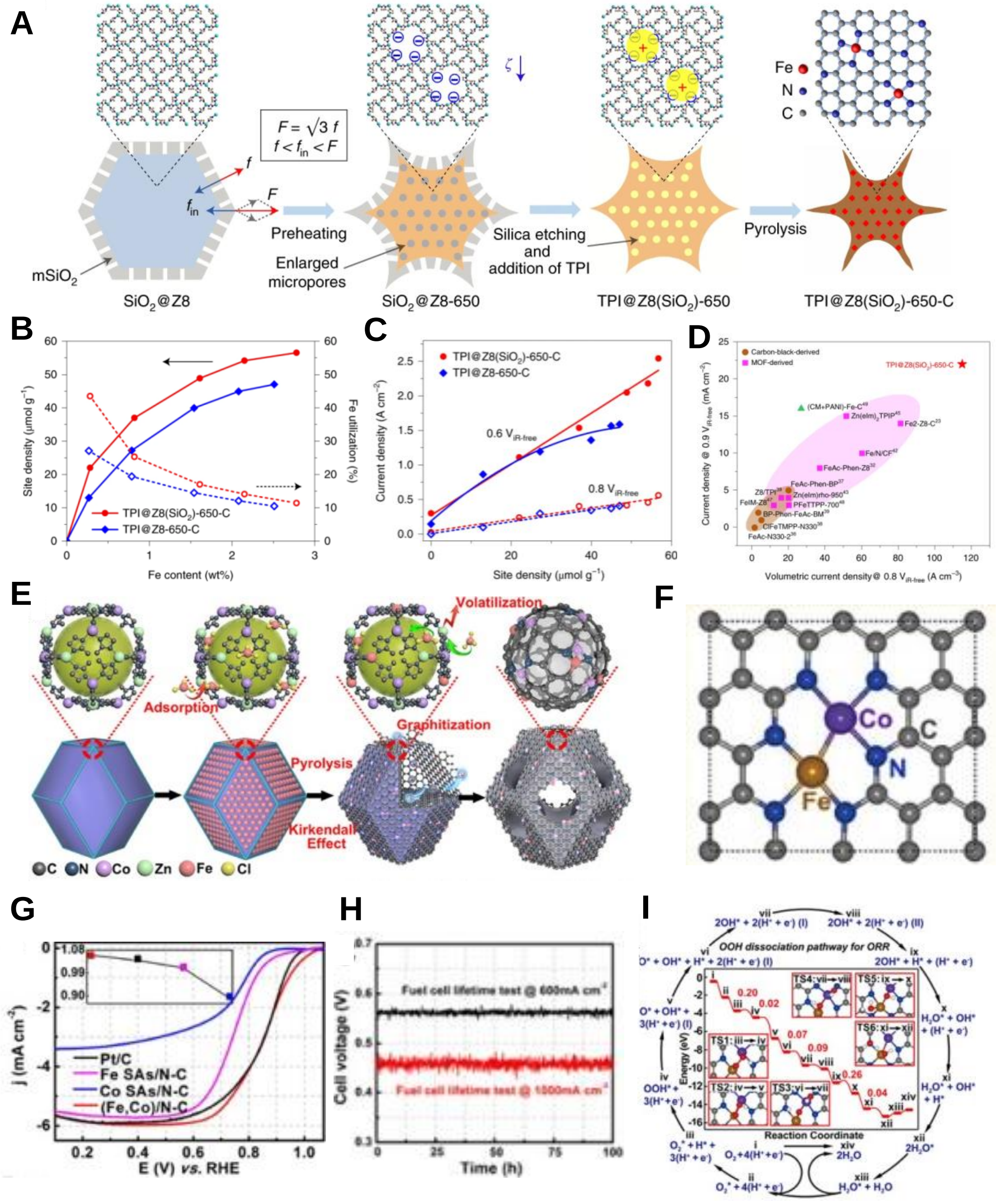

Figure S3. MOF-derived hollow porous materials and their application in ORR. (A) Schematic synthesis process of TPI@Z8(SiO_2)-650-C. (B) The SD and Fe utilization of indicated catalysts as a function of Fe content. (C) The PEMFC current density as a function of SD of indicated Fe-N-C catalysts (voltages of 0.8 and 0.6 $V_{iR\text{-}free}$, test conditions: 80 °C, 100% RH, 2.5 bar H_2-O_2). (D) PEMFC activity comparison between TPI@Z8(SiO_2)-650-C and the literature.[9] (E) Preparation of (Fe,Co)/N-C and (F) proposed architectures of Fe-Co dual sites. (G) RDE polarization curves of (Fe,Co)/N-C and reference sample in O_2-saturated 0.1 M $HClO_4$ with sweep rate 10 mV s^{-1} and rotation rate 1600 rpm. (H) Stability of (Fe,Co)/N-C in a H_2/air fuel cell measured at 600 mA cm^{-2}

and 1000 mA cm^{-2}. (I) Energies of intermediates and transition states in mechanism of ORR at (Fe,Co)/N-C from DFT.[10] Copyright Spring Nature and American Chemical Society.

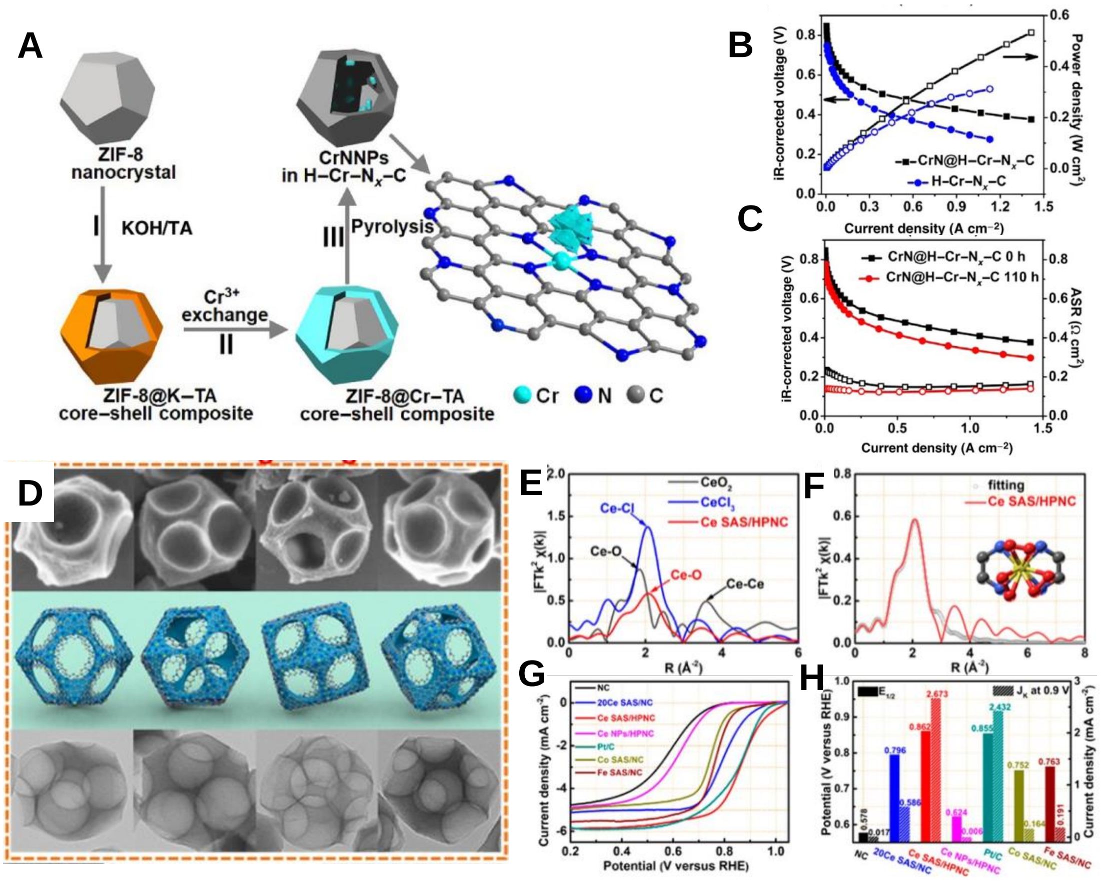

Figure S4. MOF-derived hollow porous materials and their application in ORR. (A) Synthetic scheme for the preparation of CrN@H-Cr-N_x-C. (B) I-V polarization and power density curves for H_2-O_2 PEMFC and (C) fuel cell durability tests of catalysts.[11] (D) Images of Ce SAS/HPNC corresponding to the models from different angles. (E) FT-EXAFS spectra of Ce SAS/HPNC, and reference sample at the Ce L_3-edge. (F) FT-EXAFS fitting curve of the sample and proposed architectures of Ce-N_4/O_6. (G) ORR performance of Ce SAS/HPNC and reference samples (electrolyte 0.1 M $HClO_4$, sweep rate 10 mV s^{-1}, rotating rate 1600 rpm). (H) Comparison of $E_{1/2}$ and J_K of different catalysts.[12] Copyright CCS Chemistry and American Chemical Society.

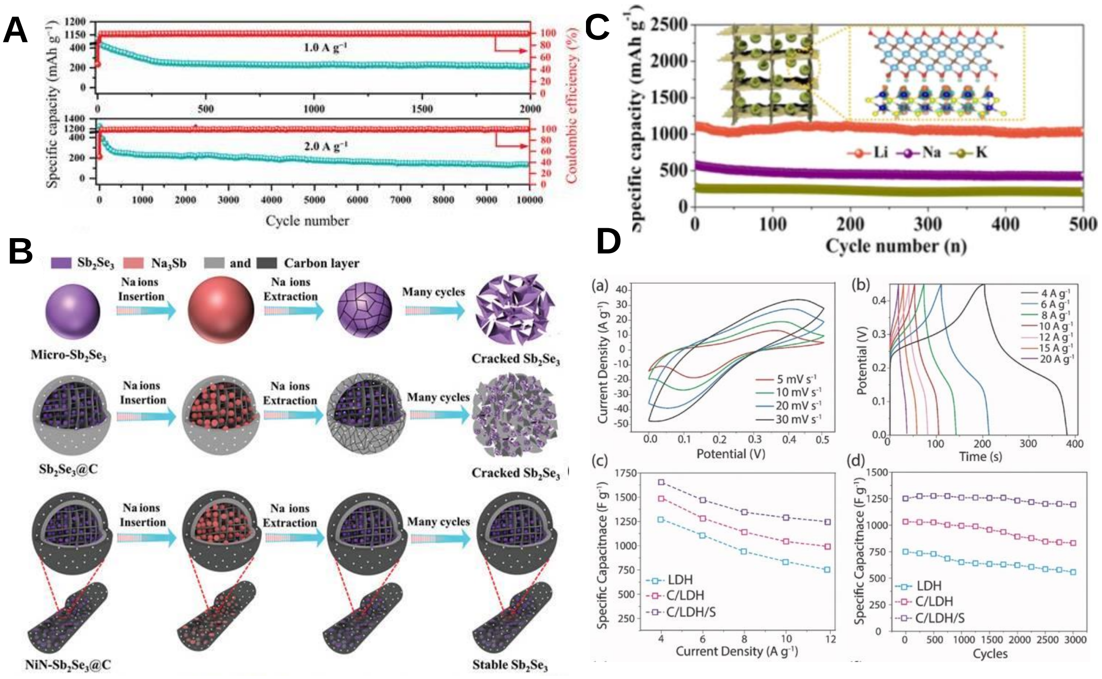

Figure S5. MOF-derived hollow porous materials and their application energy storage and conversions. (A) Cycling performance of NiN-Sb_2Se_3@C electrodes. (B) Schematic illustration of the structure evolution of different electrodes.[13] (C) The long-term cycling stability of (CoS NP@NHC)@MXene for LIBs, SIBs), and PIBs.[14] (D) Supercapacitive performance of NiCo-LDH/Co9S8, (a) CV curves at various scan rates, (b) charge-discharge profiles at various current densities, (c)specific capacitances as a function of current density, (d) cycling stability tests over 3000 cycles.[15] Copyright American Chemical Society, and John Wiley & Sons.

Table S1 A summary of synthetic strategies of MOFs- derived hollow porous materials.

Materials type	Derived from	Preparation strategy	Ref.
Yolk-shell $ZnO@SiO_2$	Core-shell ZIF-8@SiO_2	ZIF-8 as removable templates	16
Yolk–shell ZIF-8@SiO_2	Core-shell ZIF-8@SiO_2	ZIF-8 as removable templates	16
Hollow SiO_2	Core-shell ZIF-8@SiO_2	ZIF-8 as removable templates	16
Hollowparticle	ZIF@shell composite material	ZIF-8 as sacrificial templates	17
Hollow composite nanostructures	ZIF@shell composite material	ZIF-8 as sacrificial templates	17
Hollow ZIF/NP yolk@shell material	ZIF@shell composite material	ZIF-8 as sacrificial templates	17
Mg-Co LDH nanocages.	ZIF-67(Co)	ZIF-67 as both self-sacrificing templates and precursors	18
Ni-Co LDH nanocages	ZIF-67(Co)	ZIF-67 as both self-sacrificing templates and precursors	18
NiCoMn-OH	ZIF-67(Co)	ZIF-67 as both self-sacrificing templates and precursors	19
FeOOH@$Ni(OH)_2$	MIL-53(Fe)	MIL-53 as both self-sacrificing templates and precursors	20
Co^{2+}-Co^{3+} LDH	ZIF-67(Co)	ZIF-67 as both self-sacrificing templates and precursors	21
Ni-Fe LDH nanocages	MIL-88A	MIL-88A as both self-sacrificing templates and precursors	22
Hollow Co_3O_4 tetrahedra	Soild Co-based MOFs	MOFs as both self-sacrificing templates and precursors	23
Hollow Fe_2O_3 nanostructures	MIL-53 (Fe)	MIL-53 as both self-sacrificing templates and precursors	24
Multilayer CuO@NiO	Ni-Co-BTC MOF	Ni-Co-BTC MOF as both self-sacrificing templates and precursors	25
ZIF-67/Ni-Co LDH yolk-shell structure	ZIF-67(Co)	ZIF-67 as both self-sacrificing templates and precursors	26
Co_3O_4@$NiCo_2O_4$ core-shell nanocages	ZIF-67(Co)	ZIF-67 as both self-sacrificing templates and precursors	26
$Ni_xCo_{3-x}O_{4-y}$ nanocage	ZIF-67(Co)	ZIF-67 as both self-sacrificing templates and precursors	27
Hollow CuO-$CuCo_2O_4$ dodecahedrons	ZIF-67(Co)	ZIF-67 as both self-sacrificing templates and precursors	28
$ZnO/ZnCo_2O_4$ core-shell nanocages	ZIF-8	ZIF-8 as both self-sacrificing templates and precursors	29
Hollow NiO_x/Co_3O_4	ZIF-67(Co)	ZIF-67 as both self-sacrificing templates and precursors	30
Hollow carbon matrix	ZIF-8	ZIF-8 as both self-sacrificing templates and precursors	31
NPS-HCS	ZIF-8@PZS	ZIF-8@PZS composites as precursors	32

NC@Co-NGC nanocage	ZIF-8@ZIF-67 core-shell	ZIF-8@ZIF-67 core-shell composites as precursors	33
NiO_x/Ni@C composites	Ni-MOFs	Ni-MOFs composites as precursors	34
Hierarchical CoS_2 hollow prisms	ZIF-67 hollow prisms	ZIF-67 hollow prisms as precursors	35
Zn/Ni-MOF-2 hollow nanocubes	Zn/Ni-MOF-5	Zn/Ni-MOF-5 composites as precursors	36
3D hollow SOM-ZIF-8	ZIF-8@PSs	ZIF-8@PSs composites as precursors	37

Table S2 Summary of OER and HER performance of MOFs derived hollow porous electrocatalysts.

Materials	Application	Performance	Electrolyte	Ref.
Co/CoS/Fe-HSNC	OER	J=10 mA cm^{-2}, η=250 mV	1.0 M KOH	38
FeS_2–CoS_2/NCFs	OER	1.57 V at 10 mA cm^{-2}	0.1 M KOH	39
FeNiCo@NC-P	OER	1.54 V at 10 mA cm^{-2}	0.1 M KOH	40
Co-CNTs/NS-CoO_xAlO_y	OER	J=10 mA cm^{-2}, η=280 mV	0.1 M KOH	41
CoNCNTF/CNFs	OER	1.61 V at 10 mA cm^{-2}	0.1 M KOH	42
Co/Co_3O_4@PGS	OER	1.58 V at 10 mA cm^{-2}	0.1 M KOH	43
CoNi-SAs/NC	OER	J=10 mA cm^{-2}, η=340 mV	in alkaline media	44
Co@hNCTs-800	OER	1.63 V at 10 mA cm^{-2}	0.1 M KOH	45
Capsular-MOF	OER	1.59 V at 10 mA·cm^{-2}	1.0 M KOH	46
Co_3O_4/HNCP-40	OER	J=10 mA cm^{-2}, η=333 mV	1.0 M KOH	47
NC@GC	OER	1.59 V at 10 mA cm^{-2}	1.0 M KOH	48
		J=10 mA cm^{-2}, η=132 mV	1.0 M KOH	
Ni-CoP/HPFs	HER	J=10 mA cm−2, η=144 mV	0.5 M H_2SO_4	49
		J=10 mA cm−2, η=92 mV	1.0 M KOH	

Table S3 Summary of MOFs derived hollow porous materials for supercapacitor applications.

Materials	Capacity (F g^{-1})	Current density (A g^{-1})	scan rates (mV s^{-1})	BET/cm g^{-1}	Ref.
Co_9S_8@NiO	1627	1.0	/	42.62	50
NiCoMn-S	2098.2	1.0	/	94.9	51
CoS-NP/CoS-NS DSNBs	980	1.0	/	110	52
$Ti_3C_2T_X$/ZIF67/CoV_2O_6	253.8	5.0	100	/	53
NPC530	158	0.05	/	3040	54
MC-A	208	/	2	1674	55
Z-900	257	/	5	1075	56
AS-ZC-800	211	/	10	1972	57
NPC	226	/	5	1523	58
NC@GC	216	2	/	1276	59
MOF-DC	238	0.15	/	2714	60
Co-MOF	103	0.6	/	2900	61
UIO-66(Zr)	104	/	5	596	62
Ni-BDC	395	1.4		117	63
Ni-BDC-24	394	0.5	/	/	64

Co-BDC derived Co_3O_4	69	0.5	/	47.12	65
Fe-MIL-88B-NH_2 derived Fe_3O_4	139	0.5	/	37.7	66
MIL-101 (Cr) derived Cr_2O_3	180	/	2	438	67
Ce-BTC derived CeO_2	201	0.2	/	77	68
MOF-199 derived copper oxide	300	/	2	/	69
ZIF-67 derived hollow Co_3O_4	440	1.25	/	128	70
MOF-Ni-CoP@C@CNT	425	1	/	97	71
$Co(VO_3)_2$ $Co(OH)_2$	402	0.5	/	/	72
CC@$NiCo_2O_4$	633	2.5	/	11.6	73
MOF-Co_9S_8	1252	1	/	/	74
MOF-NiS	744	1	/	/	75
Ni–Zn–Co–S NSAs	1289	/	10	/	76
Co_3O_4-C/$Ni_2P_2O_7$	1142	1	/	/	77
C-ZIF-8	100	/	20	925	78

Table S4 Abbreviations

Abbreviation	Full title
BPA	Bisphenol A
BTC	Benzene-1,3,5-tricarboxylate
CCT	Colloidal crystal template
CD	Charge-discharge
CdI	Couble-layer capacitance
CHE	Computational hydrogen electrode
CM	Carbon matrix
CNTs	Carbon nanotubes
CO_2RR	CO_2 reduction reactions
DABCO	1,4-diazabicyclo [2.2.2] octane, as a linker
DFT	Density functional theory
DOE	Department of Energy
DPGG	1,2-dipalmitoyl-sn-glycero-3-galloyl
$E_{1/2}$	Positive half-wave potential
E_{onset}	Onset potential
FT-EXAFS	Fouriertransformed extended X-ray absorption fine structure
FESEM	Field emission scanning electron microscopy
HER	Hydrogen evolution reaction
HMF	5-hydroxymethylfurfural
HPC	Hollow porous carbons
HPMs	Hollow porous materials
J_k	Kinetic current density
K-TA	Polymer potassium tannate
LDH	Layered Double Hydroxide
LIBs	Lithium-ion batteries
LiPSs	Lithium polysulfides
MNPs	Metal nanoparticles
MO	Methyl orange
MOFs	Metal-organic frameworks
MOP	Metal−organic polyhedral
NCNTFs	N-doped carbon nanotube frameworks
NHPC	Hollow nitrogen-doped porous carbon
NPs	Nanoparticles
NPS-HCS	N, P, S-doped carbon shell
OCV	Open-circuit voltage
OER	Oxygen evolution reaction
ORR	Oxygen reduction reaction
PCPs	Porous coordination polymers
PIBs	Potassium-ion batteries
PMPS	Peroxymonosulfate
PZS	Cyclotriphosphazene-co-4,4’-sulfonyldiphenol
PS	Polystyrene microsphere

RH	Relative humidity
RhB	Rhodamine B
SIBs	Sodium-ion batteries
TEM	Transmission electron microscopy
TEOS	Tetraethylorthosilicate
ZABs	Zn-air batteries
ZIF-8@Cr-TA	ZIF-8@chromium-tannic acid

References

[1] Jiang, Z., Li, Z.P., Qin, Z.H., et al. (2013). LDH nanocages synthesized with MOF templates and their high performance as supercapacitors. Nanoscale ***5***, 11770-11775.

[2] Wang, W., Yan, H., Anand, U., et al. (2021). Visualizing the conversion of metal-organic framework nanoparticles into hollow layered double hydroxide nanocages. J. Am. Chem. Soc. ***143***, 1854-1862.

[3] Guo, W., Sun, W., Lv, L.P., et al. (2017). Microwave-assisted morphology evolution of Fe-based metal-organic frameworks and their derived Fe_2O_3 nanostructures for Li-ion storage. ACS Nano ***11***, 4198-4205.

[4] Guo, W., Sun, W., Wang, Y. (2015). Multilayer CuO@NiO hollow spheres: microwave-assisted metal-organic-framework derivation and highly reversible structure-matched stepwise lithium storage. ACS Nano ***9***, 11462-11471.

[5] Guo, Y., Feng, L., Wu, C., et al. (2020). Confined pyrolysis transformation of ZIF-8 to hierarchically ordered porous Zn-NC nanoreactor for efficient CO_2 photoconversion under mild conditions. J. Catal. ***390***, 213-223.

[6] Liu, W., Huang, J., Yang, Q., et al. (2017). Multi-shelled hollow metal-organic frameworks. Angew. Chem. Int. Edit. ***56***, 5512-5516.

[7] Lee, J., Kwak, J. H., Choe, W. (2017). Evolution of form in metal-organic frameworks. Nat. Commu. ***8***, 1-8.

[8] Liu, X.Y., Zhang, F., Goh T.W., et al. (2018). Using a multi-shelled hollow metal-organic framework as a host to switch the guest-to-host and guest-to-guest interactions. Angew. Chem. Int. Ed. ***57***, 2110-2114.

[9] Wan, X., Liu, X., Li, Y., et al. (2019). Fe-N-C electrocatalyst with dense active sites and efficient mass transport for high-performance proton exchange membrane fuel cells. Nat. Catal. ***2***, 259-268.

[10] Wang, J., Huang, Z., Liu, W., et al. (2017). Design of N-coordinated dual-metal sites: a stable and active Pt-free catalyst for acidic oxygen reduction reaction. J. Am. Chem. Soc. ***139***, 17281-17284.

[11] Yang, H., Wang, X., Zheng, T., et al. (2021). CrN-encapsulated hollow Cr-NC capsules boosting oxygen reduction catalysis in PEMFC. CCS Chemistry ***3***, 208-218.

[12] Zhu, M., Zhao, C., Liu, X., et al. (2021). Single atomic cerium sites with a high coordination number for efficient oxygen reduction in proton-exchange membrane fuel cells. ACS Catal. ***11***, 3923-3929.

[13] Li, Q., Zhang, W., Peng, J., et al. (2022). Nanodot-in-nanofiber structured carbon-confined Sb_2Se_3 crystallites for fast and durable sodium storage. Adv. Funct. Mater. 2112776.

[14] Yao, L., Gu, Q.M., Yu, X.B. (2021). Three-dimensional MOFs@MXene aerogel composite derived MXene threaded hollow carbon confined CoS nanoparticles toward advanced alkali-ion batteries. ACS Nano ***15***, 3228-3240.

[15] Yilmaz, G., Yam, K.M., Zhang, C., et al. (2017). In situ transformation of MOFs into layered double hydroxide embedded metal sulfides for improved electrocatalytic and supercapacitive performance. Adv. Mater. ***29***, 1606814.

[16] He, L., Li, L., L. Zhang, L.Y., et al. (2014) ZIF-8 templated fabrication of rhombic dodecahedron-shaped ZnO@SiO_2, ZIF-8@SiO_2 yolk-shell and SiO_2 hollow nanoparticles. Crystengcomm ***16***, 6534-6537.

[17] Yang, H., Kruger, P.E., Telfer, S.G., et al. (2015). Metal-organic framework nanocrystals as sacrificial templates for hollow and exceptionally porous titania and composite materials. Inorg. Chem. ***54***, 9483-9490.

[18] Jiang, Z., Li, Z.P., Qin, Z.H., et al. (2013). LDH nanocages synthesized with MOF templates and their high performance as supercapacitors. Nanoscale ***5***, 11770-11775.

[19] Du, Y.Q., Li, G.Y., Chen, M.D., et al. (2019). Hollow nickel-cobalt-manganese hydroxide polyhedra via MOF templates for high-performance quasi-solid-state supercapacitor. Chem. Eng. J. ***378***, 122210.

[20] Wang, J., Li, S., Lin, R., et al. (2019). MOF-derived hollow β-FeOOH polyhedra anchored with α-Ni(OH)2 nanosheets as efficient electrocatalysts for oxygen evolution. Electrochim Acta. ***301***, 258-266.

[21] Bai, X., Liu, J., Liu, Q., et al. (2017). In-situ fabrication of MOF-derived Co-Co layered double hydroxide hollow nanocages/graphene composite: a novel electrode material with superior electrochemical performance. Chemistry A European Journal ***23***, 14839-14847.

[22] Zhang, J., Yu, L., Chen, Y., et al. (2020). Designed formation of double-shelled Ni-Fe layered-double-hydroxide nanocages for efficient oxygen evolution reaction. Adv. Mater. ***32***, 1906432.

[23] Tian, D., Zhou, X.L., Zhang, Y.H., et al. (2015). MOF-derived porous Co_3O_4 hollow tetrahedra with excellent performance as anode materials for lithium-ion batteries. Inorg. Chem. ***54***, 8159-8161.

[24] Guo, W., Sun, W., Lv, L.P., et al. (2017). Microwave-assisted morphology evolution of Fe-based metal-organic frameworks and their derived Fe_2O_3 nanostructures for Li-ion storage. ACS Nano ***11***, 4198-4205.

[25] Guo, W., Sun, W., Wang, Y. (2015). Multilayer CuO@NiO hollow spheres: microwave-assisted metal-organic-framework derivation and highly reversible structure-matched stepwise lithium storage. ACS Nano ***9***, 11462-11471.

[26] Hu, H., Guan, B., Xia, B., et al. (2015). Designed formation of $Co_3O_4/NiCo_2O_4$ double-shelled nanocages with enhanced pseudocapacitive and electrocatalytic properties. J. Am. Chem. Soc. ***137***, 5590-5595.

[27] Antony, R.P., Satpati, A.K., Bhattacharyya, K., et al. (2016). MOF derived nonstoichiometric $Ni_xCo_{3-x}O_{4-y}$ nanocage for superior electrocatalytic oxygen evolution. Adv. Mater. Interfaces, 3, 1600632.

[28] Zhen, S.Y., Wu, H.T., Wang, Y., et al. (2019). Metal-organic framework derived hollow porous $CuO\text{-}CuCo_2O_4$ dodecahedrons as a cathode catalyst for LiO_2 batteries. RSC Adv. 9, 16288-16295.

[29] Qu, F., Jiang, H., Yang, M. (2016). Designed formation through a metal organic framework route of $ZnO/ZnCo_2O_4$ hollow core-shell nanocages with enhanced gas sensing properties. Nanoscale ***8***, 16349-16356.

[30] Chen, X., Li, J.J., Chen, X., et al. (2018). MOF-templated approach for hollow NiO_x/Co_3O_4 catalysts: enhanced light-driven thermocatalytic degradation of toluene. ACS Appl. Nano. Mater. ***1***, 2971-2981.

[31] Zhang, W., Jiang, X., Zhao, Y., et al. (2017). Hollow carbon nanobubbles: monocrystalline MOF nanobubbles and their pyrolysis. Chem. Sci. ***8***, 3538-3546.

[32] Yang, S., Peng, L., Huang, P., et al. (2016). Nitrogen, phosphorus, and sulfur Co-doped hollow

carbon shell as superior metal-free catalyst for selective oxidation of aromatic alkanes. Angew. Chem. ***128***, 4084-4088.

[33] Liu, S., Wang, Z., Zhou, S., et al. (2017). Metal-organic-framework-derived hybrid carbon nanocages as a bifunctional electrocatalyst for oxygen reduction and evolution. Adv. Mater. ***29***, 1700874.

[34] Lv, Z., Fan, Q., Xie, Y., et al. (2019). MOFs-derived magnetic chestnut shell-like hollow sphere NiO/Ni@C composites and their removal performance for arsenic(V). Chem. Eng. J. ***362***, 413-421.

[35] Yu, L., Yang, J.F., Lou, X.W. (2016). Formation of CoS2 nanobubble hollow prisms for highly reversible lithium storage. Angew. Chem. Int. Edit. ***128***, 13620-13624.

[36] Zhang, Z., Chen, Y., He, S., et al. (2014). Hierarchical Zn/Ni-MOF-2 nanosheet-assembled hollow nanocubes for multicomponent catalytic reactions. Angew. Chem. Int. Edit. ***53***, 12517-12521.

[37] Shen, K., Zhang, L., Chen, X., et al. (2018). Ordered macro-microporous metal-organic framework single crystals. Science ***359***, 206-210.

[38] Yan, L., Wang, H., Shen, J., et al. (2021). Formation of mesoporous Co/CoS/Metal-NC@S, N-codoped hairy carbon polyhedrons as an efficient trifunctional electrocatalyst for Zn-air batteries and water splitting. Chem. Eng. J. ***403***, 126385.

[39] Shi, X., He, B., Zhao, L., et al. (2021). FeS_2-CoS_2 incorporated into nitrogen-doped carbon nanofibers to boost oxygen electrocatalysis for durable rechargeable Zn-air batteries. J. Power Sources ***482***, 228955.

[40] Ren, D., Ying, J., Xiao, M., et al. (2020). Hierarchically porous multimetal-based carbon nanorod hybrid as an efficient oxygen catalyst for rechargeable zinc-air batteries. Adv. Funct. Mater. ***30***, 1908167.

[41] Najam, T., Shah, S.S.A., Ding, W., et al. (2019). Enhancing by nano-engineering: Hierarchical architectures as oxygen reduction/evolution reactions for zinc-air batteries. J. of Power Sources ***438***, 226919.

[42] Ji, D.X., L. F., Li, L.L., et al. (2019). Hierarchical catalytic electrodes of cobalt-embedded carbon nanotube/carbon flakes arrays for flexible solid-state zinc-air batteries. Carbon ***142***, 379-387.

[43] Jiang, Y., Deng, Y.P., Fu, J., et al. (2018). Interpenetrating triphase cobalt-based nanocomposites as efficient bifunctional oxygen electrocatalysts for long-lasting rechargeable zn-air batteries. Adv. Energy Mater. ***8***, 1702900.

[44] Han, X., Ling, X., Yu, D., et al. (2019). Atomically dispersed binary Co-Ni sites in nitrogen-doped hollow carbon nanocubes for reversible oxygen reduction and evolution. Adv. Mater. ***31***, 1905622.

[45] Zhou, Q., Zhang, Z., Cai, J., et al. (2020). Template-guided synthesis of Co nanoparticles embedded in hollow nitrogen doped carbon tubes as a highly efficient catalyst for rechargeable Zn-air batteries. Nano Energy ***71***, 104592.

[46] Wei, Y.S., Zhang, M., Kitta, M., et al. (2019). A single-crystal open-capsule metal-organic framework. J. Am. Chem. Soc. ***141***, 7906-7916.

[47] Ding, D., Shen, K., Chen, X., et al. (2018). Multi-level architecture optimization of MOF-templated Co-based nanoparticles embedded in hollow N-doped carbon polyhedra for efficient OER and ORR. ACS Catal. 8, 7879-7888.

[48] Wang, Z., Lu, Y., Yan, Y., et al. (2016). Core-shell carbon materials derived from metal-organic frameworks as an efficient oxygen bifunctional electrocatalyst. Nano Energy ***30***, 368-378.

[49] Pan, Y., Sun, K., Lin, Y., et al. (2019). Electronic structure and d-band center control engineering over M-doped CoP (M= Ni, Mn, Fe) hollow polyhedron frames for boosting hydrogen production. Nano energy ***56***, 411-419.

[50] Wang, J., Huang, Y., Han, X., et al. (2021). Construction of hierarchical Co_9S_8@NiO synergistic microstructure for high-performance asymmetric supercapacitor. J. Colloid. Interf. Sci. ***603***, 440-449.

[51] Kang, C., Ma, L., Chen, Y., et al. (2022). Metal-organic framework derived hollow rod-like NiCoMn ternary metal sulfide for high-performance asymmetric supercapacitors. Chem. Eng. J. ***427***, 131003.

[52] Hu, H., Guan, B.Y., Lou, X.W. (2016). Construction of complex CoS hollow structures with enhanced electrochemical properties for hybrid supercapacitors. Chem ***1***, 102-113.

[53] Liu, C., Bai, Y., Li, W., et al. (2022). In situ growth of three-dimensional MXene/Metal-organic framework composites for high-performance supercapacitors. Angew. Chem. Int. Edit., ***134***, e202116282.

[54] Liu, B., Shioyama, H., Jiang, H., et al. (2010). Metal-organic framework (MOF) as a template for syntheses of nanoporous carbons as electrode materials for supercapacitor. Carbon ***48***, 456-463.

[55] Hu, J., Wang, H., Gao, Q., et al. (2010). Porous carbons prepared by using metal-organic framework as the precursor for supercapacitors. Carbon ***48***, 3599-3606.

[56] Chaikittisilp, W., Hu, M., Wang, H., et al. (2012). Nanoporous carbons through direct carbonization of a zeolitic imidazolate framework for supercapacitor electrodes. Chem.

Commun. ***48***, 7259-7261.

[57] Amali, A.J., Sun, J.K., Xu, Q. (2014). From assembled metal-organic framework nanoparticles to hierarchically porous carbon for electrochemical energy storage. Chem. Comm. ***50***, 1519-1522.

[58] Salunkhe, R.R., Kamachi, Y., Torad, N.L., et al. (2014). Fabrication of symmetric supercapacitors based on MOF-derived nanoporous carbons. J. Mater. Chem. A ***2***, 19848-19854.

[59] Tang, J., Salunkhe, R.R., Liu, J., et al. (2015). Thermal conversion of core-shell metal-organic frameworks: a new method for selectively functionalized nanoporous hybrid carbon. J. Am. Chem. Soc. ***137***, 1572-1580.

[60] Banerjee, A., Upadhyay, K. K., Puthusseri, D., et al. (2014). MOF-derived crumpled-sheet-assembled perforated carbon cuboids as highly effective cathode active materials for ultra-high energy density Li-ion hybrid electrochemical capacitors (Li-HECs). Nanoscale ***6***, 4387-4394.

[61] Diaz, R., Orcajo, M.G., Botas, J.A., et al. (2012). Co8-MOF-5 as electrode for supercapacitors. Mater. Lett. ***68***, 126-128.

[62]Tan, Y., Zhang, W., Gao, Y., et al. (2015). Facile synthesis and supercapacitive properties of Zr-metal organic frameworks (UiO-66). RSC Adv. ***5***, 17601-17605.

[63] Yan, Y., Gu, P., Zheng, S., et al. (2016). Facile synthesis of an accordion-like Ni-MOF superstructure for high-performance flexible supercapacitors. J. Mater. Chem. A ***4***, 19078-19085.

[64] Yang, J., Xiong, P., Zheng, C., et al. (2014). Metal-organic frameworks: a new promising class of materials for a high performance supercapacitor electrode. J. Mater. Chem. A ***2***, 16640-16644.

[65] Meng, F., Fang, Z., Li, Z., et al. (2016). Porous Co_3O_4 materials prepared by solid-state thermolysis of novel Co-MOF crystal and their superior storage-energy performances for supercapacitors. J. Mater. Chem. A, ***1***, 1.

[66] Meng, W., Chen, W., Zhao, L., et al. (2014). Porous Fe_3O_4/carbon composite electrode material prepared from metal-organic framework template and effect of temperature on its capacitance. Nano Energy, ***8***, 133-140.

[67] Ullah, S., Khan, I.A., Choucair, M., et al. (2015). A novel Cr_2O_3-carbon composite as a high performance pseudo-capacitor electrode material. Electrochimi. Acta ***171***, 142-149.

[68] Maiti, S., Pramanik, A., Mahanty, S. (2014). Extraordinarily high pseudocapacitance of metal organic framework derived nanostructured cerium oxide. Chem. Commun. ***50***, 11717-11720.

[69] Khan, I.A., Badshah, A., Nadeem, M.A., et al. (2014). A copper based metal-organic framework as single source for the synthesis of electrode materials for high-performance supercapacitors and glucose sensing applications. Int. J. Hydrogen. Energ. ***39***, 19609-19620.

[70] Zhang, Y.Z., Wang, Y., Xie, Y.L., et al. (2014). Porous hollow Co_3O_4 with rhombic dodecahedral structures for high-performance supercapacitors. Nanoscale ***6***, 14354-14359.

[71] Gu, J., Sun, L., Zhang, Y., et al. (2020). MOF-derived Ni-doped CoP@C grown on CNTs for high-performance supercapacitors. Chem. Eng. J. ***385***, 123454.

[72] Zhang, Y., Chen, H., Guan, C., et al. (2018). Energy-saving synthesis of MOF-derived hierarchical and hollow $Co(VO_3)_2$-$Co(OH)_2$ composite leaf arrays for supercapacitor electrode materials. ACS appl. Mater. Inter. ***10***, 18440-18444.

[73] Guan, C., Liu, X., Ren, W., et al. (2017). Rational design of metal-organic framework derived hollow $NiCo_2O_4$ arrays for flexible supercapacitor and electrocatalysis. Adv. Energy Mater. *7*, 1602391.

[74] Yang, Q., Wang, Q., Long, Y., et al. (2020). In situ formation of Co_9S_8 quantum dots in MOF-derived ternary metal layered double hydroxide nanoarrays for high-performance hybrid supercapacitors. Adv. Energy Mater. ***10***, 1903193.

[75] Qu, C., Zhang, L., Meng, W., et al. (2018). MOF-derived α-NiS nanorods on graphene as an electrode for high-energy-density supercapacitors. J. Mater. Chem. A ***6***, 4003-4012.

[76] Huang, Y., Quan, L., Liu, T., et al. (2018). Construction of MOF-derived hollow Ni-Zn-Co-S nanosword arrays as binder-free electrodes for asymmetric supercapacitors with high energy density. Nanoscale ***10***, 14171-14181.

[77] Zhou, Y., Li, X., Li, J., et al. (2019). MOF-derived Co_3O_4-C/$Ni_2P_2O_7$ electrode material for high performance supercapacitors. Chem. Eng. J. ***378***, 122242.

[78] Tang, J., Salunkhe, R.R., Zhang, H., et al. (2016). Bimetallic metal-organic frameworks for controlled catalytic graphitization of nanoporous carbons. Sci. Rep. ***6***, 30295.
