# Supplementary material for: Metal-organic framework nanocrystal-derived hollow porous materials: Synthetic strategies and emerging applications
Source: Innovation (Camb). 2022 Jul 6;3(5):100281. doi: 10.1016/j.xinn.2022.100281 (PMC9307687; doi:10.1016/j.xinn.2022.100281)
Supplement: Document S2. Article plus supplemental information [file mmc2.pdf]

Review

Metal-organic framework nanocrystal-derived hollow porous materials: Synthetic strategies and emerging applications

Xiaolu Liu,[1,2] Gaurav Verma,[3] Zhongshan Chen,[1] Baowei Hu,[2] Qifei Huang,[4] Hui Yang,[1,*] Shengqian Ma,[3,*] and Xiangke Wang[1,2,*]
*Correspondence: h.yang@ncepu.edu.cn (H.Y.); shengqian.ma@unt.edu (S.M.); xkwang@ncepu.edu.cn (X.W.)
Received: April 16, 2022; Accepted: June 29, 2022; Published Online: July 6, 2022; https://doi.org/10.1016/j.xinn.2022.100281
© 2022 The Author(s). This is an open access article under the CC BY license (http://creativecommons.org/licenses/by/4.0/).

GRAPHICAL ABSTRACT

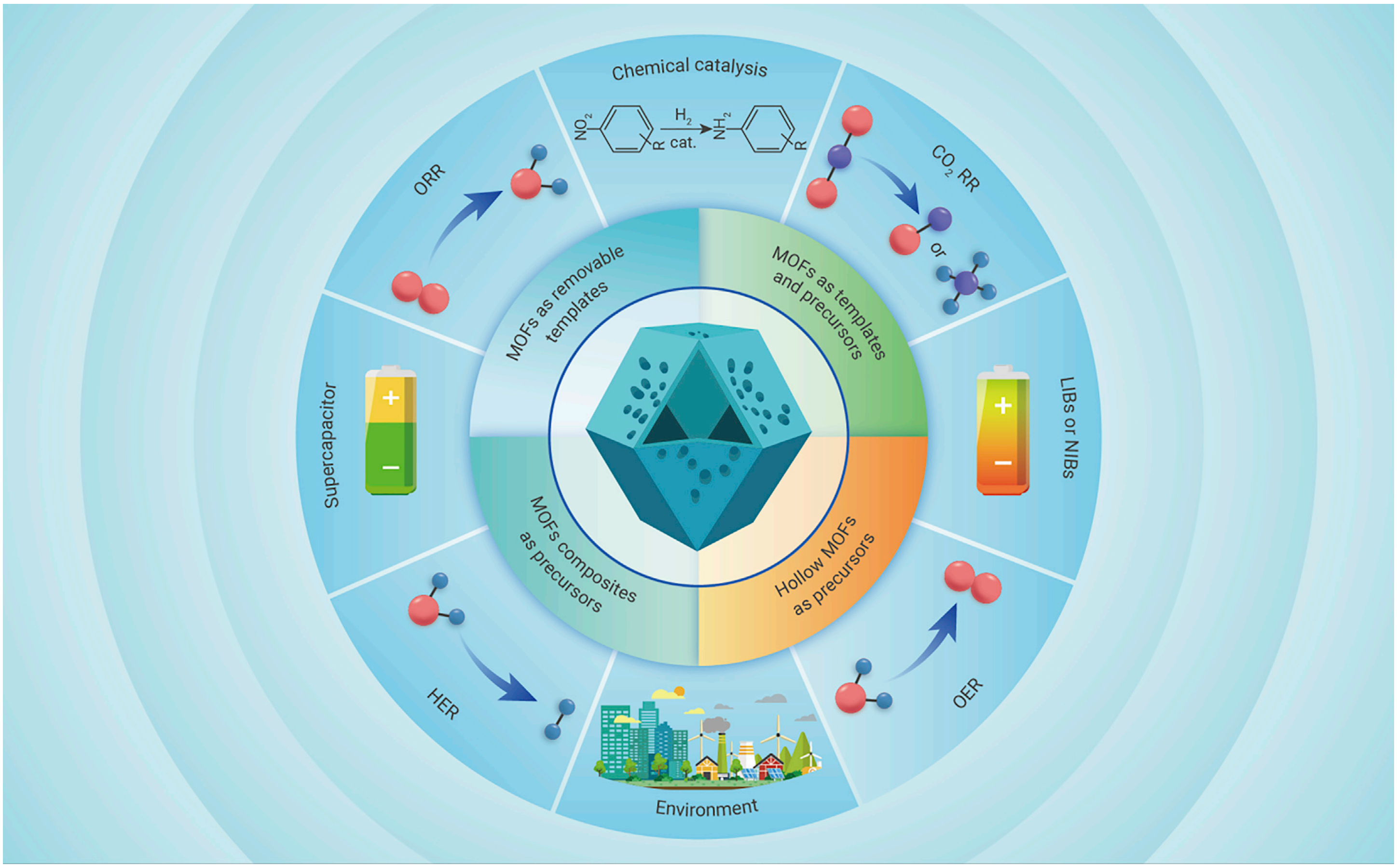

PUBLIC SUMMARY

- MOFs have garnered multi-disciplinary attention due to their unique inherent properties
- Various synthetic strategies of MOFs-derived hollow porous materials are summarized
- Emerging applications of MOFs-derived hollow porous materials are reviewed

Metal-organic framework nanocrystal-derived hollow porous materials: Synthetic strategies and emerging applications

Xiaolu Liu,[1,2] Gaurav Verma,[3] Zhongshan Chen,[1] Baowei Hu,[2] Qifei Huang,[4] Hui Yang,[1,*] Shengqian Ma,[3,*] and Xiangke Wang[1,2,*]

[1]College of Environmental Science and Engineering, North China Electric Power University, Beijing 102206, China

[2]School of Life Science, Shaoxing University, Huancheng West Road 508, Shaoxing 312000, China

[3]Department of Chemistry, University of North Texas, 1508 W Mulberry Street, Denton, TX 76201, USA

[4]State Key Laboratory of Environmental Criteria and Risk Assessment, Chinese Research Academy of Environmental Sciences, Beijing 100012, China

*Correspondence: h.yang@ncepu.edu.cn (H.Y.); shengqian.ma@unt.edu (S.M.); xkwang@ncepu.edu.cn (X.W.)

Received: April 16, 2022; Accepted: June 29, 2022; Published Online: July 6, 2022; https://doi.org/10.1016/j.xinn.2022.100281

© 2022 The Author(s). This is an open access article under the CC BY license (http://creativecommons.org/licenses/by/4.0/).

Citation: Liu X., Verma G., Chen Z., et al., (2022). Metal-organic framework nanocrystal-derived hollow porous materials: Synthetic strategies and emerging applications. The Innovation **3(5)**, 100281.

Metal-organic frameworks (MOFs) have garnered multidisciplinary attention due to their structural tailorability, controlled pore size, and physicochemical functions, and their inherent properties can be exploited by applying them as precursors and/or templates for fabricating derived hollow porous nanomaterials. The fascinating, functional properties and applications of MOF-derived hollow porous materials primarily lie in their chemical composition, hollow character, and unique porous structure. Herein, a comprehensive overview of the synthetic strategies and emerging applications of hollow porous materials derived from MOF-based templates and/or precursors is given. Based on the role of MOFs in the preparation of hollow porous materials, the synthetic strategies are described in detail, including (1) MOFs as removable templates, (2) MOF nanocrystals as both self-sacrificing templates and precursors, (3) MOF@secondary-component core-shell composites as precursors, and (4) hollow MOF nanocrystals and their composites as precursors. Subsequently, the applications of these hollow porous materials for chemical catalysis, electrocatalysis, energy storage and conversion, and environmental management are presented. Finally, a perspective on the research challenges and future opportunities and prospects for MOF-derived hollow materials is provided.

INTRODUCTION

Metal-organic frameworks (MOFs) or porous coordination polymers (PCPs), which consist of inorganic metal nodes linked by organic linkers via coordination bonds, represent a new class of crystalline porous materials.[1,2] During the past few decades, MOF materials have allowed extraordinary achievements in both the synthesis of novel nanostructures and a wide variety of potential applications, such as energy storage and conversion, sensing, catalytic reaction, environmental management, and so on. MOFs have highly ordered crystalline framework structures, and these structures are durable enough to remove the contained guest species and yield permanent pores. In particular, through the rational design and/or control of organic ligands, metal nodes, and synthesis environment, the structure and related peculiarity of MOFs can be conveniently adjusted and modified to meet specific requirements. These attractive advantages are particular to MOFs and not easily accessible in most of the other traditional porous nanomaterials.

Due to their excellent intrinsic attributes, MOFs can be used as precursors or templates to fabricate MOF-derived secondary hollow porous nanomaterials (HPMs): metal carbides, metal oxides, metal sulfides, layered double hydroxides, porous carbon materials, and their hybrid composites. MOF-derived porous nanomaterials have also played a significant role in photocatalysis, energy storage and conversion, and particularly electrocatalysis, owing to their superior attributes, such as large specific surface area, micro- or mesoporosity for efficient electron and mass transport during the reaction, inherited structural versatility, high tolerance to acid/alkaline environments, and electrical conductivity, as well as diverse chemical components.

Among these MOF-derived hollow nanomaterials, discrete, hollow structures have garnered considerable attention. In most cases, the synthesis of MOF-derived HPMs essentially relies on a decomposition of the MOF or its composites under a certain condition or chemical reaction with desirable reagents: (1) hollow SiO_2 derived from a secondary constituent of MOF@shell composite, (2) hollow porous carbon materials derived from the organic components of the MOF and its shell or guest molecules, and (3) hollow metal oxides, sulfides, carbides, supported MNPs, etc., derived from the metallic components of the MOF or MOF composite. Traditional synthetic methods for HPMs require a hard template, for example, SiO_2, and polystyrene (PS), while a significant drawback is that harsh conditions are required for template removal. Compared with the traditional hard template method, the conditions of preparing hollow porous materials from MOFs as a template and precursor system are mild, and there is no need to remove the template. Furthermore, in contrast to the traditional molecular sieve and amorphous porous materials, hollow nanomaterials derived from MOFs demonstrate numerous advantages, including uniform hollow morphology and chemical constituents, tunable distribution of chemical and structural compositions, and tunable porosity. Benefiting from these attributes, MOF-derived hollow nanomaterials exhibit excellent performance in chemical catalysis, electrocatalysis, energy storage and conversion, and other applications.

Although several reviews have summarized the MOF-derived hollow nanomaterials and their potential applications,[3–9] most of these review articles are mainly focused on MOF-derived hollow materials and their applications in energy storage and conversion. Up to now, a comprehensive summary of the synthetic strategies and emerging applications of hollow porous materials derived from MOF-based templates and/or precursors is lacking in the literature. In this context, we aim to provide a systematic and detailed overview of the most recent achievements of MOFs as templates and/or precursors for fabricating hollow nanomaterials and their application in chemical catalysis, electrocatalysis, environmental management, energy storage, and energy conversions. Finally, this review concludes with some personal insights that we hope open a broad new avenue for future directions in this attractive research field.

DESIGN STRATEGIES FOR MOF-DERIVED HOLLOW POROUS MATERIALS

For this part, we will provide various detailed synthetic strategies for fabricating HPMs, such as hollow porous carbons and their composites, hollow layered double hydroxides, hollow metal-based compounds, and their composites (Table S1).

MOF nanocrystals as removable templates

Usually, the sacrificial template controls the size and morphology of the hollow inorganic nanostructures. He and co-workers proposed a synthetic route for SiO_2-based hollow nanocrystals with tunable components.[10] As shown in Figure 1A, a layer of SiO_2 was uniformly coated on ZIF nanocrystals from a soluble TEOS precursor through the Stöber method. This produces a ZIF-8@SiO_2 core-shell material. Subsequently, hollow SiO_2-based materials were obtained from the ZIF-8@SiO_2 core-shell composite by different ways of treatment: (1) a ZnO@SiO_2 yolk-shell composite was formed by calcination in the air or (2) a ZnO@SiO_2 yolk-shell composite and a hollow SiO_2 polyhedron were formed by thermal treatment in acidic solution at 100°C for 2 and 24 h, respectively. Inspired by the above research, we proposed a route to prepare hollow TiO_2 using ZIF nanocrystals as a removable template and gave rare examples of hollow cubic and polyhedral morphologies (Figure 1B). It was particularly interesting to see that the surface of ZIF-8 can promote the growth of TiO_2 coatings.[11] The ZIF-8 core could be removed completely and quickly without affecting the integrity of the TiO_2 shells. The sacrificial ZIF-8 template controls the shape and size of the obtained hollow TiO_2 structure. These TiO_2 materials demonstrated exceptional textural properties. Upon using ZIF-8 nanocrystals injected with Pt nanoparticles (NPs) as templates, the Pt NPs were successfully encapsulated into the hollow TiO_2 cubes.[11] Brinker and colleagues also proposed a novel concept for the functionalization of the surface of MOFs based on the direct coordination of a phenolic-inspired lipid molecule, DPGG (Figure 1C). Consequentially,

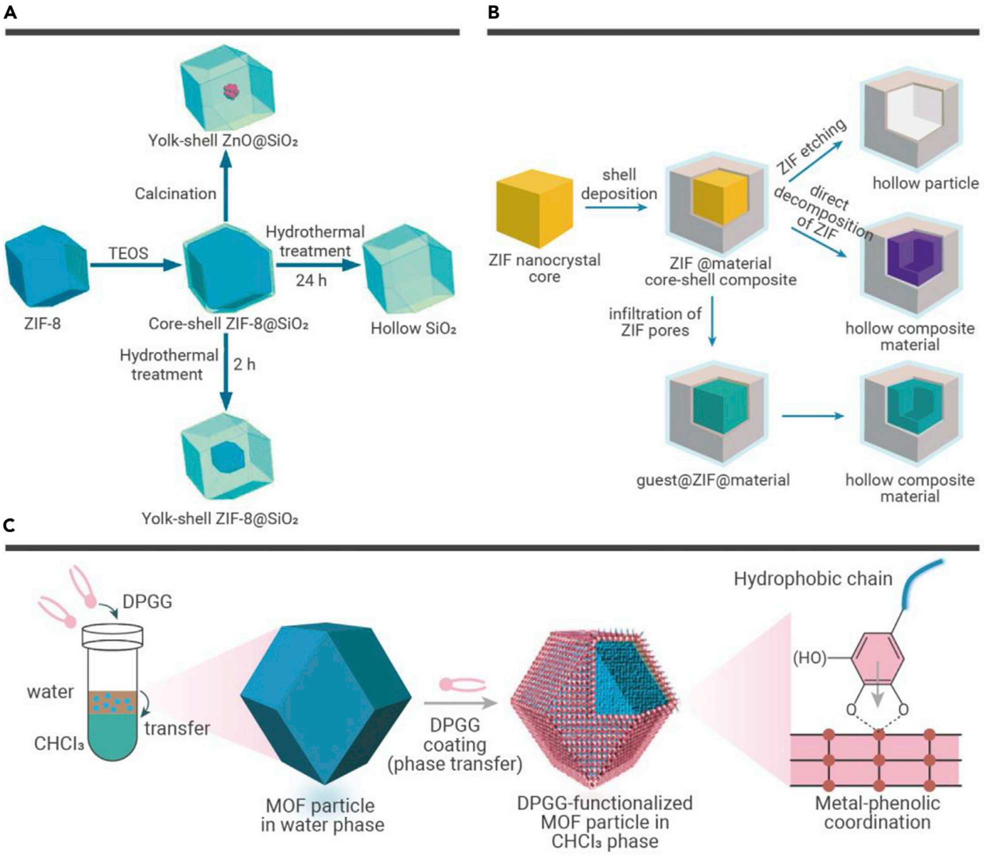

Figure 1. Synthesis of hollow nanocrystals (A and B) Schematic illustrations of the strategies for using ZIF nanocrystals as sacrificial templates for the synthesis of (A) hollow SiO_2-based structures[10] and (B) hollow TiO_2-based structures.[11] (C) Schematic illustration of the surface functionalization of MOF particles by a phase transfer reaction.[12] Copyright Royal Society of Chemistry, American Chemical Society, and John Wiley & Sons.

MIL-88 nanocrystals and UiO-66 were also used as a removable template for the production of the hollow polymer.[12]

Taken together, when an MOF is used as a template to fabricate hollow materials, the shape and size of the obtained hollow structures are controlled by the sacrificial MOF template. And the other advantage is that the MOF template can be completely and rapidly eliminated without affecting the integrity of the hollow shells.

MOF nanocrystals as both self-sacrificing templates and precursors

In addition to the removable templates, MOF nanocrystals can be used as both self-sacrificing templates and precursors for fabricating hollow nanostructures. Many approaches have been developed to synthesize MOF derivatives, for instance, hollow metal hydroxides, metal sulfides, metal oxides, metal phosphides, hollow carbon matrixes, and so on.[13–16] ZIF-8 (Zn) and ZIF-67 (Co) were applied as self-sacrificial template and precursor to prepare M-Co-LDH (M = Mg, Co, Ni) nanocages with hollow structures (Figure S1A).[14] Control of the simultaneous reactions, the precipitation of the shells, and the template etching is extremely crucial to the preparation of perfect nanocages. Subsequently, similar synthesis strategies were used for producing other LDH and metal hydroxide nanocages, such as Mg-Co LDH,[13,14] Ni-Co LDH,[14] Co^{2+}-Co^{3+} LDH,[17] Ni-Fe LDH,[18] and a series of metal hydroxides, including Co-OH, NiCo-OH, Ni_2Co-Mn_1-OH, Ni_1Co-Mn_2-OH, NiCoMn-OH-AD, CoMn-OH, and FeOOH@$Ni(OH)_2$.[15,16] The process of MOF-derived hollow LDH could be observed by *in situ* technical characterization: etching of the NPs and growth of LDHs on the NP surfaces.[19] The conversion process of ZIF-8 nanocubes and ZIF-8 rhombic dodecahedrons into LDH nanocages is observed by *in situ* transmission electron microscopy (TEM). Conversion of ZIF-67@ZIF-8 core-shells into "shell-in-shell" LDH nanocages is demonstrated in Figures S1B and S1C.

The metal components of the MOF template are known to be converted into metal oxide by oxidation and pyrolysis in an air atmosphere. Thus, MOF nanomaterials are very suitable as sacrificial templates and precursors for producing various hollow metal oxides. Hollow Co_3O_4 tetrahedrons were successfully synthesized through the thermolysis of $[Co_3L_2(TPT)_2 \cdot xG]_n$ (G = guest molecules) at 773 K for 4 h in the air.[20] Guo et al. fabricated hollow Fe_2O_3 nanostructures with an octahedron and a yolk-shell octahedron by calcinating MIL-53 (Fe) in the air for 2 and 6 h, respectively (Figure S1D).[21] Other hollow metal oxides, such as ZnO,[22] CeO_2,[23] In_2O_3,[24] and V_2O_5[25] were also synthesized by using the different MOF precursors. These works provided guidance for the fabrication of multicomponent hollow metal oxides composed of two or more different metals. To achieve this target, it is necessary to dope the secondary metal components into the precursor prior to thermolysis. Guo et al. reported a simple and novel method to synthesize multilayer CuO@NiO with hollow spheres by using Ni-Co-BTC MOF as a sacrifice template and precursor (Figure S1E).[26] The ZIF-67/Ni-Co LDH yolk-shell nanostructure was prepared using a chemical etching ZIF-67 nanocrystalline surface in a methanol solution containing $Ni(NO_3)_2$. Subsequently, the Co_3O_4@$NiCo_2O_4$ core-shell structure nanocages were formed from ZIF-67/Ni-Co LDH by thermal treatment under air conditions.[27] Those synthetic approaches were further applied to fabricate other hollow multicomponent metal oxides, such as $Ni_xCo_{3-x}O_{4-y}$ nanocages,[28] NiO/ZnO hollow spheres,[29] hollow porous CuO-$CuCo_2O_4$ dodecahedrons,[30] ZnO/$ZnCo_2O_4$ hollow core-shell nanocages,[31] and hollow NiO_x/Co_3O_4.[32] Guan et al. utilized a practical and versatile strategy to fabricate multicomponent metal oxides, including Co-Ni, Co-Mn, Mn-Ni, Zn-Mn, and Co-Mn-Ni structures (Figures 2A and 2B).[33]

To prepare hollow porous carbon, MOF precursors are usually pyrolyzed under an Ar or N_2 atmosphere, and metal species will experience evaporation and/or subsequent leaching in the pyrolysis process. For example, Zhang et al. utilized ZIF-8 as a sacrificial template and precursor for fabricating a hollow carbon matrix (Figure 2C).[34] First, the inner parts of the ZIF-8 template were etched with a tannic acid solution, and then hollow ZIF-8 was treated at 873 K under an inert atmosphere, with subsequently etching of the obtained sample with acid solution to eliminate the remaining Zn species, and the hollow carbon matrix was acquired. In addition, Wang et al. proposed a host-guest strategy to fabricate a hollow N-doped porous carbon electrocatalyst with Fe-Co dual sites.[36]

Reasonable introduction of other species into MOFs and then pyrolysis can provide an excellent opportunity to manufacture the targeted hollow material with the desired compositions. For example, Wu et al. proposed an MOF-assisted strategy for synthesizing MoC_x NPs confined within hollow nano-octahedrons of porous carbon (Figure 2D).[35] They first prepared $[Cu_2(BTC)_{4/3}(H_2O)_2]_6[H_3PMo_{12}O_{40}]$ nanocrystals and then pyrolyzed this sample at 1,073 K to prepare MoC_x-Cu through an *in situ* carbonization process involving the Cu-MOF host framework and the guest $H_3PMo_{12}O_{40}$ components. $FeCl_3$ solution is used to selectively etch the Cu species to generate hollow MoC_x octahedrons (Figures 2E and 2F). Many research groups extended the fabrication method to other hollow nanostructures, such as hollow metal sulfides, metal selenides, and metal phosphides, that were synthesized by using MOF nanocrystals as both sacrificial templates and precursors.

MOF@secondary-component core-shell composites as precursors

The growth of MOF@secondary-component core-shell structures is very popular for fabricating MOF-derived hollow porous materials. Hu et al.

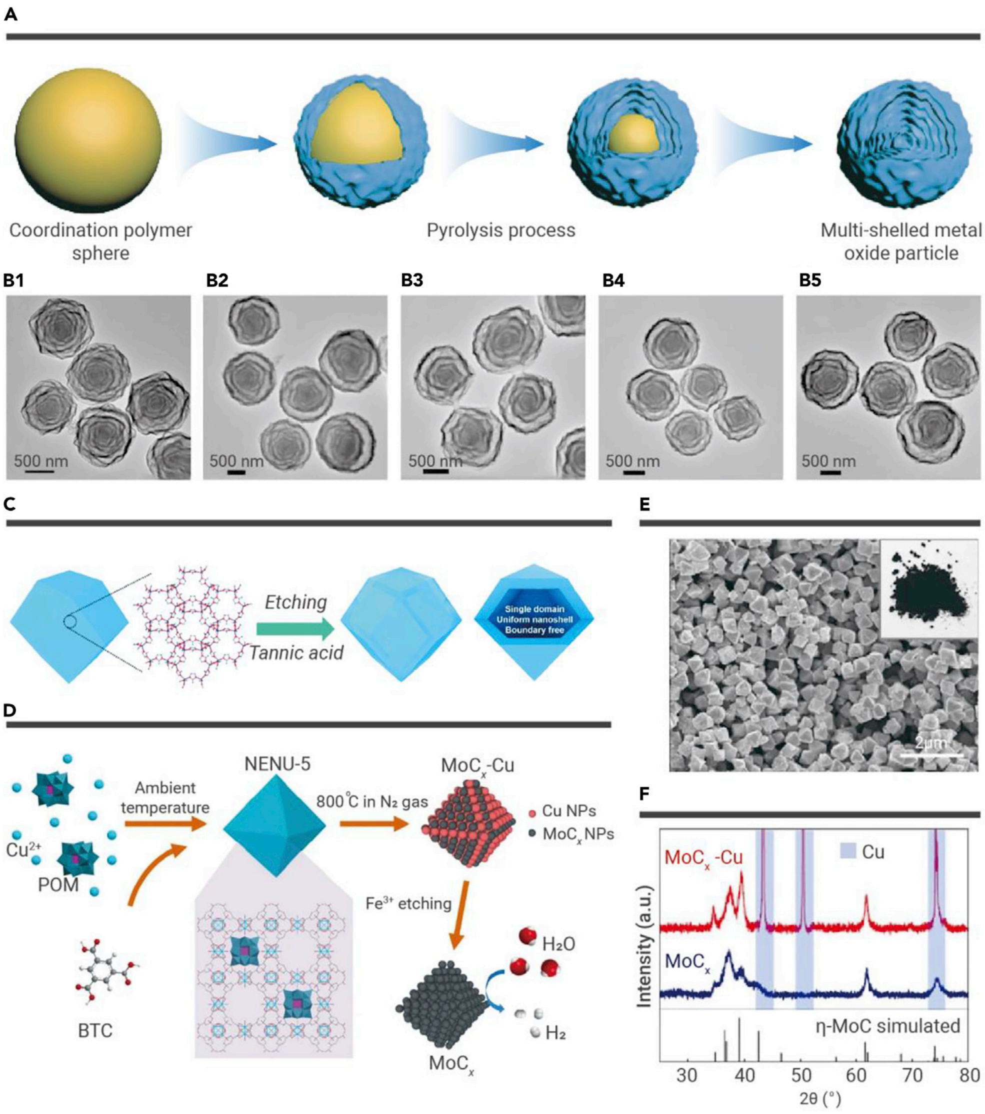

Figure 2. Schematic illustrations of strategies for using MOF nanocrystals as sacrificial templates and precursors (A) The synthesis of multicomponent metal oxides.[33] (B) FESEM images of multicomponent metal oxides: (B1) Co-Ni, (B2) Co-Mn, (B3) Mn-Ni, (B4) Zn-Mn, and (B5) Co-Mn-Ni.[33] (C) A hollow carbon matrix.[34] (D) Porous MoC_x nano-octahedrons.[35] (E) FESEM image of porous MoC_x nano-octahedrons. (F) XRD pattern spectra of MoC_x-Cu and MoC_x nano-octahedrons.[35] Copyright John Wiley & Sons, Royal Society of Chemistry, and Springer Nature.

utilized phenolic acids as surface functionalization and etching reagents to prepare hollow MOFs (Figure 3A).[37] Metal-phenolic networks (MPNs) were formed on the MOF surfaces by the coordination of phenolic acid and metal ions and finally functionalized the surface of the resulting hollow MOFs. As a seminal study, we proposed the synthetic routes toward the first N-HPC capsule, and then the N-HPC capsules encapsulated NPs with tunable components employing ZIF-8 nanocrystals as a template (Figure 3B).[38] First, ZIF-8@K-TA was prepared by coating the metal-phenolic coordination (K-TA) as an auxiliary material on the surface of the ZIF-8 crystal. Afterward, ZIF-8@K-TA was pyrolyzed under an Ar atmosphere at 900°C. It is worth noting that this process converted the organic components of ZIF-8@K-TA into hollow microcapsules with the shape of the ZIF-8 templates. During the pyrolysis process, the Zn species vaporized and escaped from the material to form N-HPC capsules. Interestingly, post-synthetic ion exchange of the K^+ ions in the K-TA shell by Co(II) or Ni(II) could be realized by facilely immersing ZIF-8@K-TA in a methanolic solution of $Co(NO_3)_2$ or $Ni(NO_3)_2$, respectively. Subsequently, monometallic Co or Ni NPs encapsulated in N-HPC capsules could be achieved by heating ZIF-8@Co-TA or ZIF-8@Ni-TA, respectively, under an Ar atmosphere. We further extended this strategy to synthesize supported multicomponent metal NPs. The heating of ZIF-8/Pt@M-TA or ZIF-8/Pt@Mmix-TA results in N-HPC capsules with Pt-based alloyed NPs embedded in the capsule walls. This approach can be extended to prepare NPs composed of four different metals.[41] Inspired by the above successes, we subsequently fabricated a series of atomically dispersed metal catalyst-decorated hollow carbon capsules, including H-Fe-N_x-C, H-Co-N_x-C, H-FeCo-N_x-C, H-FeNi-N_x-C, H-FeCoNi-N_x-C, etc.[42]

Similarly, the synthetic route toward an N, P, S-co-doped carbon shell (NPS-HCS) was reported.[39] They first prepared ZIF-67 nanocrystals and then deposited a cross-linked PZS layer on the surface of ZIF-67 to obtain a ZIF-67@PZS core-shell structure (Figure 3C). After heat treatment in Ar and acid etching, NPS-HCSs were obtained (Figure 3D). Inspired by the study, Fe single atoms supported on an N, P, S-co-doped hollow carbon polyhedron were fabricated. They revealed that the long-range interaction of the active metal center with sulfur and phosphorus facilitated the formation of a hollow structure and improved catalytic performance.[43]

Moreover, Liu et al. fabricated NC@Co-NGC nanocages via pyrolysis of a ZIF-8@ZIF-67 core-shell composite.[40] The core-shell composites were originally obtained by coating the epitaxial ZIF-67 shell on the surface of the ZIF-8 core (Figure 3E). After pyrolysis under N_2 atmosphere and subsequent etching treatment, double-shelled NC@Co-NGC nanocages with surface-anchored carbon nanotubes (CNTs) were obtained. It should be noted that the formation of hollow nanostructures can be attributed to surface-stabilized shrinkage of core-shell ZIF-8@ZIF-67 nanocrystals at high temperatures. Similarly, Pan and colleagues used core-shell ZIF-8@ZIF-67 composites to prepare hollow composites of CoP NPs embedded in N-doped CNTs through a continuous pyrolysis-oxidation-phosphorylation process.[44]

In addition, various hollow carbon[34,39] and hollow carbon-supported metal-based materials, including metals,[45] metal oxides,[46] metal sulfides, metal phosphides,[47] and metal selenides,[48] can be easily produced through the reaction of MOF@secondary-component templates with the corresponding thermolysis.

Hollow MOF nanocrystals and their composites as precursors

Similar to the previously mentioned design strategy, direct thermolysis of hollow MOFs is a facile route to fabricate functional hollow porous materials. Zou and co-workers[49] fabricated NiO/Ni/graphene composites with hierarchical hollow structure through annealing of Ni-MOF with hierarchical hollow structure. Figure 4A shows that hierarchical hollow Ni-MOF nanocrystals were prepared with a uniform diameter. Then, the pyrolysis of the hollow MOF nanocrystals under a N_2 gas environment resulted in Ni/graphene core-shell composites. After further annealing treatment under air, the final sample NiO/Ni/graphene composites were acquired.[49] Moreover, hollow ZnO/C composites,[53] NiO_x/Ni@C composites,[54] Z67-Co_3O_4/C-4, and Z9-Co_3O_4/C-4[55] were also synthesized by a similar process. Similarly, Yu et al.[50] fabricated hierarchical CoS_2 hollow prisms by using ZIF-67 as a precursor (Figure 4B). Nanosized CoS_4 bubble-like subunits were prepared via sulfidation of ZIF-67 hollow prisms with thioacetamide in ethanol solution. During the sulfidation process, Co^{2+} cations in the ZIF-67 were converted into hollow CoS_4 nanocrystals. After pyrolysis under the N_2 atmosphere, the hierarchical CoS_2 with multistage hollow interiors was acquired.[50] Moreover, Zhang et al.

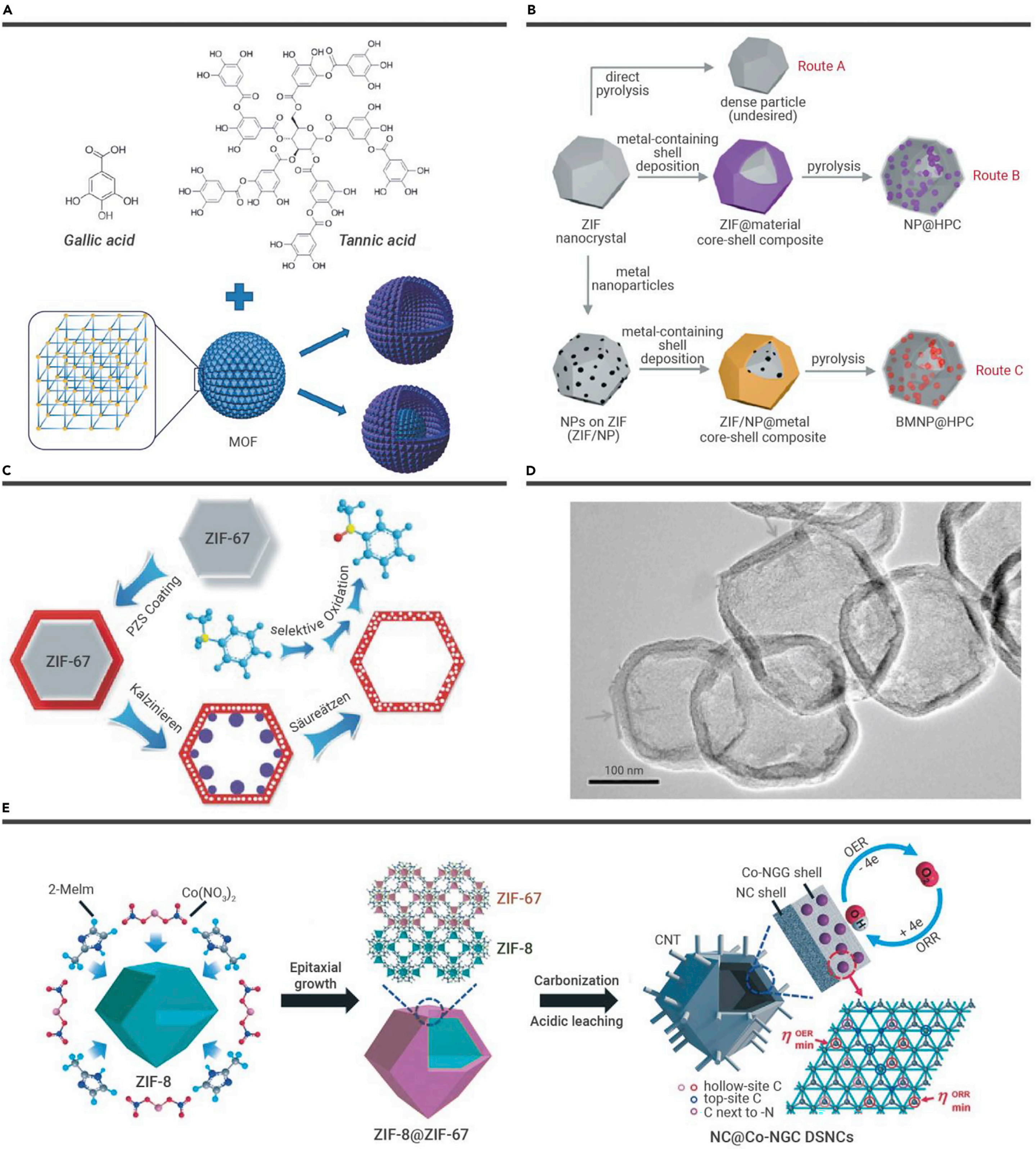

Figure 3. Schematic illustrations of strategies for using MOF nanocrystals as sacrificial templates (A–C and E) the formation of (A) hollow MOFs,[37] (B) porous carbon capsules,[38] (C) N, P, S-doped carbon shells,[39] and (E) double-shelled NC@Co-NGC nanocages.[40] (D) TEM image of NPS-HCS.[39] Copyright John Wiley & Sons and American Chemical Society.

also employed Zn/Ni-MOF-5 and nanocubes as the precursor to prepare Zn/Ni-MOF-2 nanosheet with hierarchical hollow nanocubes.[56]

External templates can also be used to construct hollow porous materials derived from MOF nanocrystals. Su and co-workers reported a multicavity hollow Au@ZIF-8 nanoreactor (Figure 4C) produced via the exterior template method.[51] As depicted in Figure 4D, silica was employed as the template source coating on the surface of Au NPs to produce Au NP@silica core-shell composites. Subsequently, the growth of ZIF-8 led to packaging of multiple Au NP@silica particles embedded in its surface to form "raisin bun"-like structures. Then, multicavity hollow Au NP@ZIF-8 nanoreactors were formed after etching the silica. The hollow products feature intrinsic monodispersed micropores and introduced macropores, and each microvoid unit structure has only one Au NP inside. NP@MOF[57] and yolk-shell nanocrystal@ZIF-8[58] were also synthesized with a similar process.

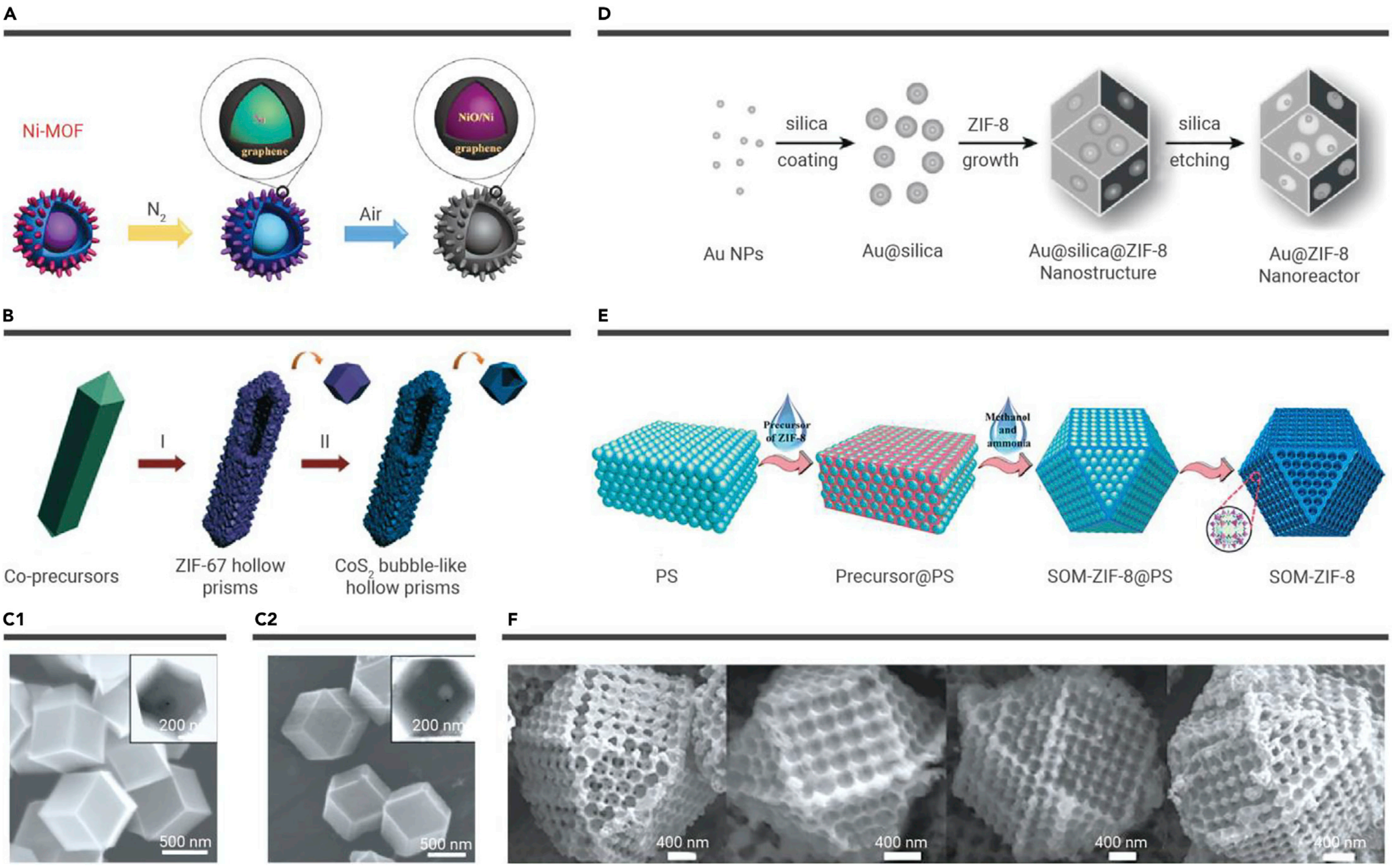

Figure 4. Schematic illustrations of strategies for using hollow MOF nanocrystals and their composites as precursors to formation (A) NiO/Ni/graphene composites.[49] (B) Hierarchical CoS_2 hollow prisms.[50] (C) TEM images of (C1) core-shell-shell Au@silica@ZIF-8 nanostructures and (C2) yolk-shell Au@silica nanostructures.[51] (D) Au@ZIF-8 nanoreactor.[51] (E) Periodic hollow SOM-ZIF-8.[52](F) SEM images of SOM-ZIF-8 were taken from four different directions.[52] Copyright John Wiley & Sons and American Chemical Society.

Moreover, hollow MOF nanocrystals and their composites have also been applied for synthesizing periodic hollow porous MOF structures. For instance,[52] 3D hollow SOM-ZIF-8 with oriented and ordered macro-micropores were acquired by packaging ZIF-8 and PS spheres (PSs) as a well-assembled "precursor@PS" template (Figure 4E). Specifically, PSs were assembled into a highly ordered 3D opal structure, and then ZIF-8 precursors filled the PS monolith interstices to form "precursor@PS." It was immersed in the CH_3OH and $NH_3 \cdot H_2O$ solution to reach a balanced process between the growth of the ZIF-8 crystal and the removal of the PSs. As a result, the highly oriented and ordered macropores SOM-ZIF-8 were obtained (Figure 4F). Subsequently, Guo et al. synthesized Zn-N-HOPCPs with ordered pores through confined growth and pyrolysis of ZIF-8 crystalline template voids.[59] As shown in Figure S2A, in the preparation of ZIF-8, SiO_2 CCT was added for the preparation of SiO_2-CCT@ZIF-8 precursors. The obtained SiO_2-CCT@ZIF-8 was pyrolyzed under N_2 conditions and then SiO_2-CCT was removed in 1 M NaOH to obtain the final products.

In addition, multishelled hollow MOFs can be fabricated by controlling crystal production and etching or self-assembly strategies. As shown in Figure S2B, Liu et al. developed a rational method to prepare single-, double-, and triple-shelled hollow MIL-101 with single-crystalline shells by step-by-step crystal growth and subsequent etching processes.[60] The cavity size and shell thickness of each layer can be tailored by rational regulation of MOF nucleation and crystallization. Choe and colleagues demonstrated the process of a solid MOP transformed to a hollow MOF with controlled layers of shells through self-assembly (Figure S2C).[61] First, they immersed solid UMOM-1 (MOP) in DABCO solution to generate a core-shell structure. The external surface of the MOP crystal was coordinated with the linker; thus, the center of the MOP can be retained even if the reaction were stopped halfway. Due to the difference in solubility, the core was selectively dissolved in methanol solution and formed a single-crystal hollow MOF with a single shell. By repeating the above process, a single-crystal hollow MOF with multiple shell layers can be obtained. Similarly, Liu and co-workers[62] synthesized a multishelled ZIF-8 by selectively dissociating ZIF-67 from the multilayered ZIF-67@ZIF-8 (Figure S2D). The shell number of hollow ZIF-8 can be tuned by controlling the epitaxial layer-by-layer overgrowth of ZIF-8 and ZIF-67. Furthermore, the interactions between different guests can also be tuned by precisely immobilizing them in MOF shells or by encapsulating them in cavities between MOF shells.[62]

APPLICATIONS OF MOF NANOCRYSTAL-DERIVED HOLLOW POROUS MATERIALS

Chemical catalysis

Chemical catalysis is a widespread phenomenon and a central topic for modern industry. To date, tremendous efforts have been committed to preparing and constructing excellent chemical catalysts. Recently, various novel MOF-derivative constructions have been reported and used in selective hydrogenation, goal-directed oxidation, CO_2 reduction reaction, and so on.[63]

Selective hydrogenation. Hollow MOF composites with noble metals possess remarkable selectivity in hydrogenation reactions. Tsung and co-workers fabricated Pd on ZIF-8, yolk-shell Pd@ZIF-8, and core-shell Pd@ZIF-8 by utilizing Pd nanocrystals and hollow ZIF-8.[58] Through the gas-phase hydrogenation reaction of ethylene, cyclohexene, and cyclooctene, the molecular size selectivity of the prepared catalysts was studied. Experimental results revealed that all of the catalysts demonstrated superior activity for ethylene hydrogenation, while only the Pd on the ZIF-8 catalyst exhibited excellent catalytic performance for the cyclooctene. This is because ethylene molecules (2.5 Å) can diffuse through the pore size (3.4 Å) of the ZIF-8 shell, while cycloalkene molecules (5.5 Å) are much larger than the pore size of the ZIF-8 shell (Figure 5A). Similarly, Yang et al. fabricated hollow Pd@ZIF-8 nanosphere catalysts with different thicknesses and studied their selectivity in liquid-phase hydrogenation.[64] As shown in Figure 5B, when Pd@ZIF-8 was applied to the hydrogenation reactions of 1-hexene, *trans*-stilbene, and tetrastyrene, experimental results showed that the smaller the size of the reactant, the higher the conversion. Moreover, Pd@ZIF-8 catalysts with thicker shells (Pd@ZIF-8(S)) possessed lower conversion efficiency. A similar conclusion was obtained when void@HKUST-1/Pd@ZIF-8

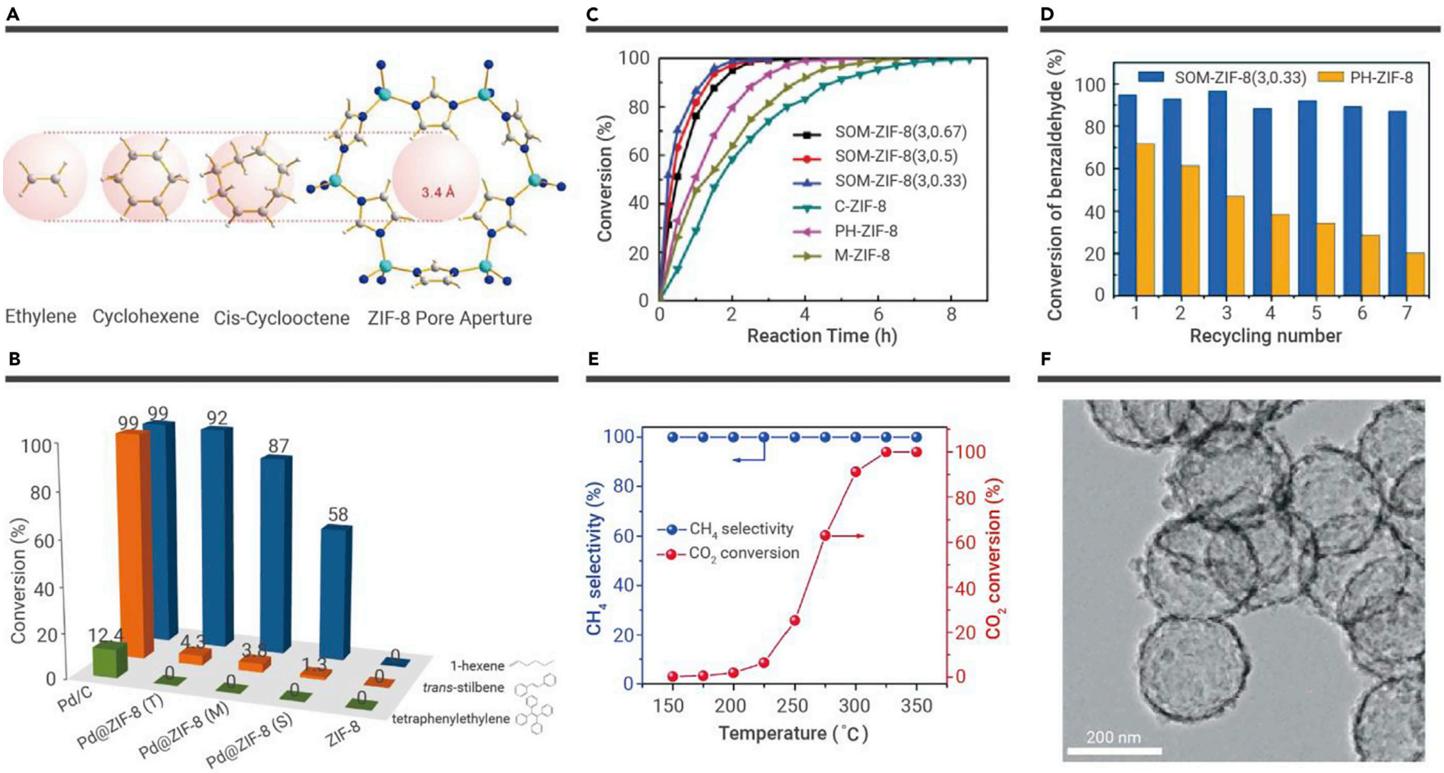

Figure 5. The application of MOF-derived hollow porous materials in chemical catalysis (A) The molecular sizes of ethylene, cyclohexene, cyclooctene, and the ZIF-8 pore aperture.[58] (B) Catalytic performance of Pd@ZIF-8 nanospheres for the liquid-phase hydrogenation of 1-hexene, *trans*-stilbene, and tetraphenylethylene with different ZIF-8 shell thicknesses.[64] (C) Benzaldehyde conversion over various samples as a function of reaction time. (D) Recyclability tests of SOM-ZIF-8 and PH-ZIF-8.[52] (E) Catalytic CO_2 methanation performance of Ni@C at different reaction temperatures.[65] (F) TEM image of HPC-800.[66] Copyright John Wiley & Sons, American Chemical Society, and Royal Society of Chemistry.

was used as a hydrogenation reaction catalyst.[67] Moreover, hollow PtAuDNP@HKUST-1 petalous heterostructures also exhibited excellent performance in the hydrogenation of olefin.[57] Noble-metal catalysts exhibited remarkable activity in selective hydrogenation, but the high cost limited their practical application in the hydrogenation reaction. One strategy is to combine noble metals with low-cost non-noble metals, which can reduce costs and maintain activity and selectivity. Analogously, yolk-shell PdCu@Fe^{III}-MOF-5,[68] yolk-shell (PTA)@CdCu@MOF-5(FeIII),[69] and hollow Pd@Zn-Co ZIF[70] exhibited excellent selective hydrogenation performance.

Goal-directed oxidation. MOF-derived hollow porous materials have demonstrated remarkable performance in goal-directed oxidation of organic matters. Shen et al. reported a single-crystalline SOM-ZIF-8 with the 3D ordering of macro-micropores.[52] They investigated the catalytic performance of SOM-ZIF-8 in the Knoevenagel reaction of benzaldehydes and malononitrile. As shown in Figures 5C and 5D, SOM-ZIF-8 catalysts possessed significantly improved performance compared with the other catalysts and exhibited superior structural stability and enhanced recyclability. Doping heteroatoms can significantly improve the catalytic ability of hollow porous materials in various organic reactions. MOF-derived hollow Co_3O_4 polyhedrons exhibited superior catalytic performance and excellent stability, and the complete conversion was up to 100% for toluene oxidation.[71] MOF-derived yolk-shell Co@C-N demonstrated prominently improved catalytic performance in the aqueous oxidation of alcohols while yielding >99% conversion. The enhanced catalytic performance is due to the unique yolk-shell structure, which can accelerate the transfer rate of reactants/products and the synergistic effect between the Co NPs and the N-doped carbon nanosheet.[72] Similarly, both MOF-derived hollow Fe-Co nanocatalyst[73] and hollow yolk-shell Co@CN catalyst[74] also afforded high productivity and excellent selectivity for oxidation of HMF. In addition, MOF derivatives can be used as catalysts for CO_2 conversion. Dai and co-workers fabricated hierarchical hollow Ni@C spheres derived from Ni-MOFs, which exhibited superior catalytic performance and enhanced stability for CO_2 reduction reaction (Figure 5E).[65] Novel HPC with ultrahigh concentrations of Zn single atoms (Figure 5F) was synthesized for catalytic CO_2 cycloaddition with epoxides under light and showed excellent performance. The hollow structure of the porous carbon cavity can convert the absorbed light energy into heat energy, which significantly improves the conversion of endothermic CO_2. Meanwhile, single Zn atoms act as a Lewis acid site, and a Lewis base site can cooperate to boost substrate activation. Moreover, the carbon shell with hierarchically porous character accelerated CO_2 enrichment and improved the transport rate of reactants/products. That was the first report on integrating the photothermic effect into endothermic CO_2 conversion.[66]

Briefly, hollow porous materials derived from MOFs have the following advantages as catalysts in industrial catalytic processes: (1) the porous MOF shell can serve as a host matrix to prevent NP aggregation and (2) be applied to realize selective catalysis, and (3) the presence of porosity allows for promoting mass transport of chemical species. It is noteworthy that the thickness of the shell has a certain degree of influence on the catalytic performance.

Electrocatalysis

ORR. Fuel cells and metal-air batteries have garnered extensive attention owing to their being a possible solution to the fossil energy shortage and increasing environmental pollution.[75] ORR is important for fuel cells and metal-air batteries.[5] The precious metal Pt is applied as an outstanding and effective four-electron transfer electrocatalyst for ORR. However, the low abundance and preciousness limit its widespread applications. Among all of the reported electrocatalysts, MOF-derived nanomaterials with high specific surface area, an abundance of accessible active sites, and improved mass/charge transfer rate have been viewed as highly beneficial for the ORR process (Table 1).

Among the studied non-noble metals, Fe and Co are the most active metal species for ORR.[76,90,91] A lot of excellent studies about Fe and Co as efficient cathode electrocatalysts have been reported. Tang and co-workers reported carbonized Fe NPs derived from MOFs (MIL-88B-NH_3), which showed excellent ORR property with E_{onset} and $E_{1/2}$ reaching 1.03 and 0.92 V (versus RHE), respectively, in alkaline medium. In real ADMFC, carbonized Fe NPs possessed high output power density and were evenly 1.7 times higher than the 20% Pt/C.[77] Guo and colleagues fabricated Co@Co_3O_4@core@bishell NPs derived from MOFs into a highly ordered porous CM to prepare the catalyst Co@Co_3O_4@C-CM used for ORR.[78] This work created a solid interaction/contact between the metal oxide and the carbon shell linked to the porous CM, which is significant for facilitating the

Table 1. Summary of ORR performance of MOF-derived hollow porous electrocatalysts

Material	Performance	Electrolyte	PEMFC/Zn-air battery	Stability	Reference
A-CoNC	$E_{1/2}$ = 0.79 V	0.1 M KOH	peak power density 144.0 mW cm^{-2}	–	Zhong et al.[76]
	E_{onset} = 0.91 V		capacity 612.3 mAh g^{-1} (Zn-air battery)		
CNPs	$E_{1/2}$ = 0.92 V	0.1 M KOH	power density 22.7 mW cm^{-2}	–	Zhao et al.[77]
	E_{onset} = 1.03 V				
$Co@Co_3O_4@C$-CM	$E_{1/2}$ = 0.7 V	0.1 M KOH	–	–	Xia et al.[78]
	E_{onset} = 0.85 V				
$TPI@Z8(SiO_2)$-650-C	–	–	power density 1.18 W cm^{-2} at 0.8 $V_{iR\text{-}free}$	–	Wan et al.[79]
			current density 0.047 A cm^{-2} at 0.88 $V_{iR\text{-}free}$		
CrN@H-Cr-N_x-C	$E_{1/2}$ = 0.72 V	0.1 M $HClO_4$	current density 0.888 A·cm^{-2}	110 h retain 77%	Yang et al.[80]
	E_{onset} = 0.85 V		peak power density 0.382 W cm^{-2} at 0.43 V		
	Tafel slope = 55 mV dec^{-1}				
Ce SAS/HPNC	$E_{1/2}$ = 0.862 V	0.1 M $HClO_4$	circuit voltage 0.95 V	–	Zhu et al.[81]
	Jk = 2.673 mA cm^{-2}		power density 0.525 W cm^{-2} at 2.0 bar		
PSTA-Co-T	$E_{1/2}$ = 0.878 V	0.1 M KOH	–	–	Wei et al.[82]
HNCSs	$E_{1/2}$ = 0.82 V	0.1 M KOH	–	10 h retain 96.5%	Chai et al.[83]
	E_{onset} = 0.92 V				
	Tafel slope = 65.7 mV dec^{-1}				
N-HPCs	$E_{1/2}$ = 0.92 V	0.1 M KOH	power density 158 mW cm^{-2} (Zn-air battery)	–	Kong et al.[84]
	E_{onset} = 1.06 V		power density 486 mW cm^{-2} (H_2-O_2 fuel cell)		
Fe/NC-700	$E_{1/2}$ = 0.854 V	0.1 M KOH	–	8 h retain 97.7%	Zhang et al.[85]
$(CoS_x$/N, S-HCS$)_{700}$	$E_{1/2}$ = 0.87 V	0.1 M KOH	–	60,000 s retain 59.6%	Xiao et al.[86]
	E_{onset} = 0.93 V				
C-PANI-MIL-2	$E_{1/2}$ = 0.87 V	0.1 M KOH	–	–	Yang et al.[87]
	E_{onset} = 1.0 V				
C-FeHZ8@g-C_3N_4-950	$E_{1/2}$ = 0.845 V	0.1 M KOH	–	80,000 s retain 91.6%	Deng et al.[88]
	E_{onset} = 0.97 V				
H-Fe-N_x-C	$E_{1/2}$ = 0.92 V	0.1 M KOH	–	–	Yang et al.[89]

electron transfer rate between NPs and the porous CM and improving mass transport of O_2 and electrolytes, making the NPs hard to detach from the porous CM support. It was found that, in alkaline medium, $Co@Co_3O_4@C$-CM displayed almost identical catalytic performance but enhanced stability and better methanol tolerance for ORR relative to the 20% Pt/C.

In addition to MOF-derived composites, MOF-derived materials with an M-N-C structure (M represents a metal atom) have also demonstrated supernormal electrocatalytic performance for ORR and have attracted widespread attention.[92] For instance, Wan et al. prepared a concave-shaped Fe-N-C SAC ($TPI@Z8(SiO_2)$-650-C) with enhanced surface area and dense Fe-N_4 sites (Figure S3A).[79] Further investigation revealed that $TPI@Z8(SiO_2)$-650-C has the special properties of additional mesoporosity, high surface area, and high exposure to Fe-N_4 active-site density (Figures S3B and S3C). The obtained catalysts showed excellent PEMFC performance, achieving current densities of 0.022 A cm^{-2} at 0.9 $V_{iR\text{-}free}$ and 0.047 A cm^{-2} at 0.88 $V_{iR\text{-}free}$, which achieved the DOE 2018 target. $TPI@Z8(SiO_2)$-650-C achieved a superior performance of 129 mA cm^{-2} at 0.8 $V_{iR\text{-}free}$ under 1 bar H_2-air and a P_{max} of 1.18 W cm^{-2} under 2.5 bar H_2-O_2. This was better than the performance reported in most of the literature (Figure S3D). Li and colleagues synthesized N-coordinated Fe/Co dual-site catalysts derived from Zn/Co bimetallic MOF-encapsulated $FeCl_3$ molecules (Figure S3E). Multiple means of characterization confirmed the presence of Fe/Co single-atom dual sites.[36] According to EXAFS results, the dual metal center was named N_3Fe-CoN_3 in Figure S3F. The catalyst with Fe-Co dual active sites exhibited remarkable ORR performance under acidic conditions with $E_{1/2}$ (0.863 V versus RHE) and enhanced stability compared with 20% Pt/C, individual Fe SAs/NC, and Co SAs/NC (Figures S3G and S3H). Density functional theory (DFT) calculations proved the single-atom dual sites can decrease the cleavage energy of O-O bonds and realize excellent performance toward ORR and high selectivity for the four-electron reduction path (Figure S3I). Recently, we reported on CrN@H-Cr-Nx-C derived from ZIF-8@Cr-TA core-shell nanocrystals (Figure S4A).[80] Due to the synergies between CrNNPs and single-atomic-site CrN_x, the enriched CrN_x sites, discrete CrNNPs, and affluent micro/mesopores, as an electrocatalyst, CrN@H-Cr-N_x-C displayed superior electrocatalytic performance for ORR in an acidic medium with outstanding OCV, and excellent current power densities were also observed when used as a cathode electrocatalyst applied in PEMFC (Figure S4B). The CrN@H-Cr-Nx-C also showed enhanced stability, proved by durability tests (Figure S4C). In addition, Ce-SAS/HPNC with a hierarchically macro/meso/microporous structure derived from MOFs can also be applied as an electrocatalyst for ORR (Figure S4D).[81] XAS analysis results verified that the Ce sites were stabilized by four coordinated N atoms and six O atoms (Ce-N_4/O_6) (Figures S4E and S4F). Remarkably, the Ce-SAS/HPNC displayed an outstanding ORR performance with E_{onset} of 1.04 V, $E_{1/2}$ of 0.862 V, and J_K of 2.673 mA cm^{-2} at 0.9 V compared with the referenced catalysts (Figures S4G and S4H). In addition, the doping of heteroatoms (such as S, P, or O) will change

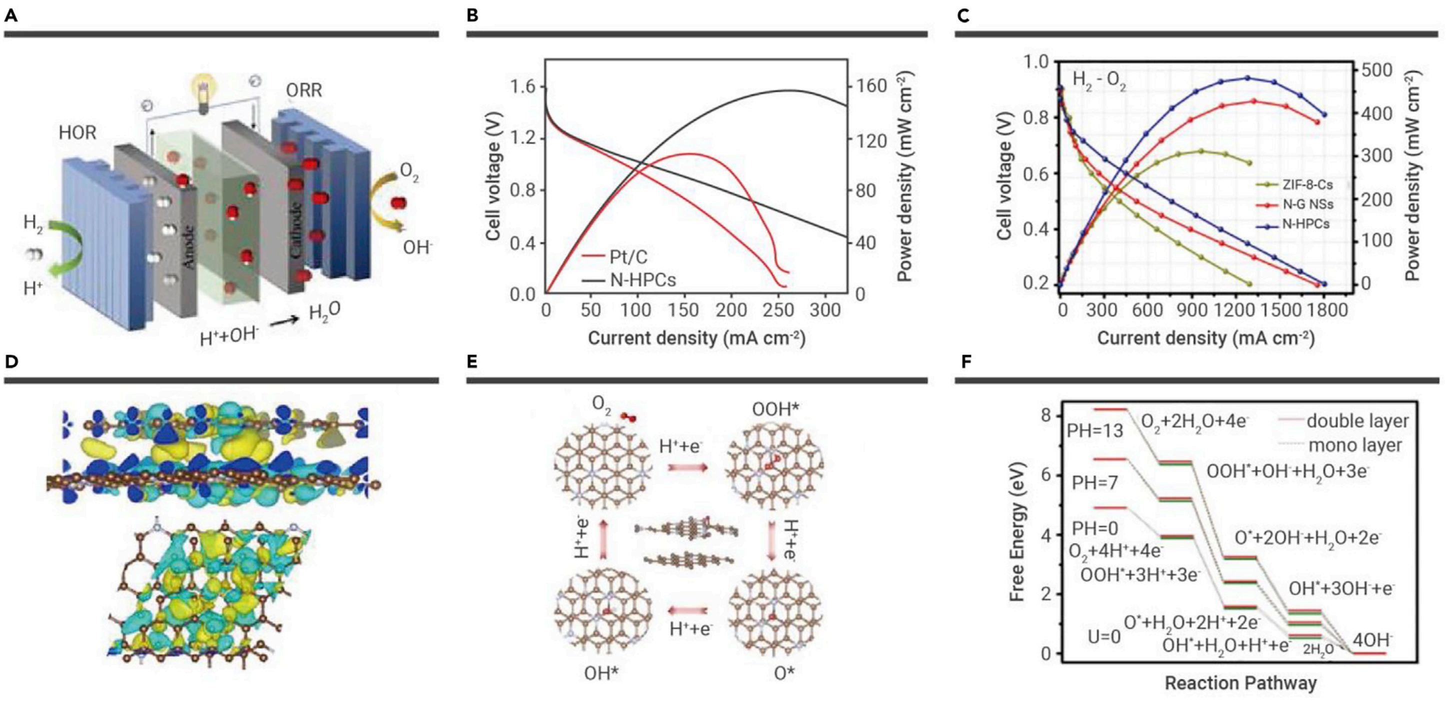

Figure 6. MOF-derived hollow porous materials and their application in ORR (A) Schematic of the primary configuration of H_2-O_2 cells. (B) Discharge polarization and the corresponding power density curves of ZABs using N-HPCs or Pt/C catalyst as the air cathode. (C) Polarization and power density plots of PEMFC using N-HPCs as cathode catalysts under H_2-O_2 (test conditions: 60°C, 100% RH, 1 bar H_2-O_2). (D) Interfacial electron transfer schematics in bilayer model (N-HPCs). (E) The reaction pathway of ORR in acidic solution. (F) Free energy diagrams for ORR at U = 0 on a double-layer structure in the whole pH range.[84] Copyright Springer Nature.

the intrinsic performance of M-N-C catalysts. In this regard, P-doped P-CNCo-20[93] and N/P-doped Co-N_2P_2[82] were also reported to have enhanced ORR performance. In addition, MOF-derived hollow heteroatom-doped carbon materials also displayed outstanding electrocatalytic ORR activity.[83]

As an attractive study, a N-doped carbon electrocatalyst with a negligible amount (0–0.08 wt %) of Fe (N-HPC) was synthesized. Such N-HPCs feature a hollow and hierarchically porous architecture, which shows excellent ORR activity and durability.[84] When used as cathode catalysts, the N-HPCs demonstrated distinguished power densities of 486 and 158 mW cm^{-2} for PEMFCs and Zn-air batteries, respectively. Interestingly, Fe sites do not contribute to ORR activity (Figures 6A–6C). Further, spin-polarized DFT calculations and CHE methodology were applied to investigate the source of the original catalytic activity of N-HPCs for ORR. The results indicated that the non-covalent-bonded N-deficient/N-rich heterostructure in the hierarchically porous architecture of N-HPCs could accelerate electron transfer between the layers and provide the active sites for oxygen adsorption and activation (Figures 6D–6F).

OER. OER plays an essential role in energy conversion technologies. To reduce the expensive cost of OER, extensive efforts have been devoted to exploiting high-performance and inexpensive OER electrocatalysts, with MOF-derived hollow materials being one of them (Table S2).[94]

According to numerous reports, MOF-derived bimetal or metal oxide NPs (especially Fe, Co, and Ni) are beneficial to OER catalysis.[95–100] For example, Lou and co-workers reported NiCoP/C (Figure 7A) to have a remarkable OER catalytic performance,[47] which displayed a low overpotential of 330 mV at 10 mA cm^{-2}, and enhanced stability of 96.5% of the initial current is retained after 10 h (Figures 7B and 7C). In addition, Yao and colleagues prepared two types of hollow Co_3O_4/C (Z67-Co_3O_4/C-4 and Z9-Co_3O_4/C-4) via carbonized and then oxidized ZIF-67 and ZIF-9 precursors.[55] Figures 7D and 7E demonstrate that the catalysts of Z67-Co_3O_4/C-4 and Z9-Co_3O_4/C-4 exhibited excellent activity for OER. This is because the unique hollow porous carbon structure improves the electron transfer rate, and vast oxygen vacancies in hollow Co_3O_4/C can improve the adsorption of water molecules to promote OER activity. Apart from MOF-derived metal oxides, MOF-derived $CoSe_2$ with a hollow structure was also synthesized as an OER electrocatalyst with improved performance.[48] The optimized $CoSe_2$-450 microspheres displayed 10 mA cm^{-2} at η = 330 mV with a small Tafel slope of 79 mV dec^{-1}, better than the reference catalysts. This is due to the hollow structure and uniformly distributed active sites, which facilitate a fast mass and electron transport rate. Integration of MOF-derived materials with metallic oxide proved to be a facile and effective strategy to design high-activity OER catalysts.[101]

MOF-derived LDH materials also showed excellent OER properties. Zhang et al. fabricated Ni-Fe LDH nanocages with tunable shells that used MIL-88A particles as sacrificial templates (Figure 7F).[18] By mixing different proportions of ethanol and water, Fe-Ni LDH single-shelled nanocages (SSNCs) and double-shelled nanocages (DSNCs) were easily acquired. Owing to more Fe elements in the outer shell, the obtained Ni-Fe LDH DSNCs with effective surface area exposure showed superior OER performance and a high C_{dl} value compared with the single shelled ones (Figure 7G).

HER. Like the OER, HER is an essential reaction for water splitting. Although the overpotential of HER is much lower than OER, electrocatalysts are still needed to improve the HER reaction.[102] Recently, earth-abundant transition metal carbides, especially MoC, have been extensively studied as high-efficiency HER catalysts under acidic and basic conditions. Mesoporous molybdenum carbide nano-octahedrons (MoC_x nano-octahedrons) were prepared through confined carburization in an MOF.[35] The unique nanostructure MoC_x nano-octahedrons showed outstanding catalytic performance for HER in acidic medium and alkaline conditions. Moreover, on repeated potential sweeps, the MoC_x nano-octahedrons exhibited promising durability in acidic and alkaline media (Figure 8A). Stability tests are demonstrated in Figure 8B. The current density of MoC_x nano-octahedrons is generally stable for more than 10 h in 0.5 M H_2SO_4, with slight degradation observed during long-term operation in 1 M KOH. A TEM image conveyed that the nanostructure and crystallinity after degradation measurement are well retained, further confirming its good stability in an acidic environment (Figure 8B). The HER activity under basic conditions is also superiorly favorable for many HER catalysts, such as graphene and CNTs, and even state-of-the-art β-Mo_2C-based electrocatalysts. Subsequently, hollow Ni-decorated molybdenum-carbide was also synthesized through a similar strategy.[104] Benefiting from the advantages of combined composition (MoC and Ni NPs) and unique structure, the obtained samples showed a small overpotential of 123 mV at 10 mA cm^{-2}, a small Tafel slope of 83 mV dec^{-1} in alkaline medium, and superior stability. Zhang et al. reported ultrafine Pt-Co alloy NPs confined on surfaces of CNTs (Pt_3Co@NCNT catalyst) and, benefiting from the synergistic effects of the bimetallic alloy components, the Pt_3Co@NCNT catalyst showed remarkably enhanced HER performance in both acidic and alkaline media. The exquisite porous carbon shell

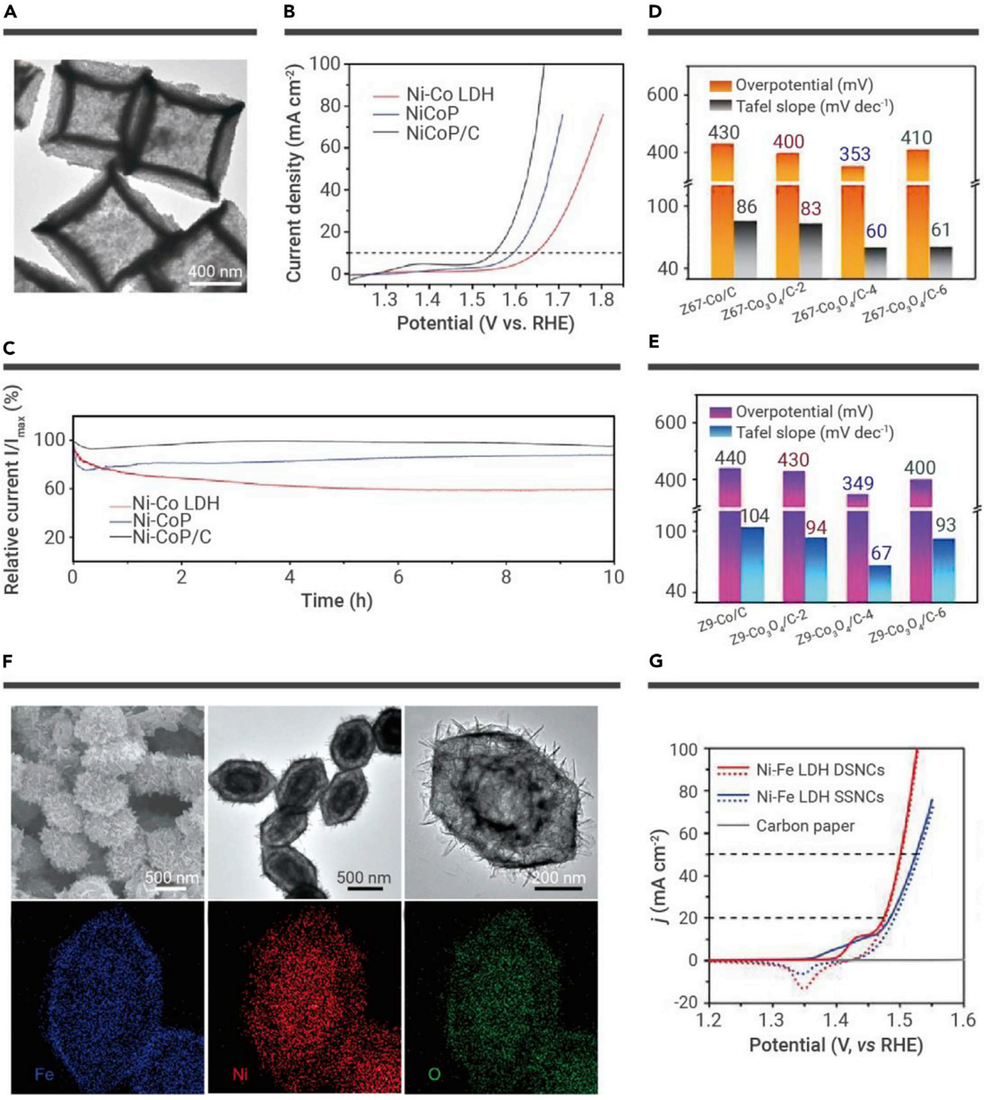

Figure 7. MOF-derived hollow porous materials and their application in OER (A) TEM image of the NiCoP/C nanoboxes. (B and C) (B) Polarization curves and (C) chronoamperometry curves of NiCoP/C nanoboxes and other samples in an O_2-saturated 1.0 M KOH solution.[47] (D and E) Summarized overpotentials at j = 10 mA cm^{-2} and Tafel plots of Z67-Co_3O_4/C-4 and Z9-Co_3O_4/C-4 in 1 M KOH solution.[55] (F) SEM, TEM, and mapping of Ni-Fe LDH nanocages. (G) CV curves of Ni-Fe LDH catalysts and carbon paper.[18] Copyright John Wiley & Sons.

structure host prevents the ultrasmall Pt_3Co NPs from aggregating, thus ensuring long-term durability.[105] In a fascinating study, Huang et al.[106] synthesized a series of hollow Co-based bimetallic sulfide ($M_xCo_{3-x}S_4$, M = Zn, Ni, and C) polyhedrals by using MOFs as self-templates. $Zn_{0.30}Co_{2.70}S_4$ exhibited outstanding electrocatalytic HER activity over a wide pH range, with overpotentials of 80, 90, and 85 mV at 10 mA cm^{-2} and 129, 144, and 136 mV at 100 mA cm^{-2} in 0.5 M H_2SO_4, 0.1 M phosphate buffer, and 1 M KOH, respectively. $Zn_{0.30}Co_{2.70}S_4$ further exhibited photocatalytic HER activity when working with an organic photosensitizer (Eosin Y) or semiconductors (TiO_2 and C_3N_4). This study provides a reference for the synthesis of transition metal sulfides used for HER.

Bifunctional electrocatalysts. Electrocatalysis, as an important process in energy conversion, usually involves more than one process. Specifically, water splitting includes HER and OER, and metal-air batteries involve ORR and OER. The ORR is also the fundamental electrochemical reaction for fuel cells. The design of bifunctional electrocatalysts is significant for the electrochemical process and puts forward higher requirements on the componential tuning and morphological control of the catalyst. MOF-derived hollow structure materials are considered high-efficiency electrocatalysts due to their unique advantages, such as tailorable composition and multilevel porosity.

Transition metals doped with heteroatoms are considered to be highly efficient electrocatalysts. Pan et al. proposed the preparation of CoP/NCNHP by a successive pyrolysis-oxidation-phosphatization process applied to core-shell-structured ZIF-8@ZIF-67 as the precursor.[44] The excellent catalytic performance of both HER and OER of CoP/NCNHP can be attributed to the synergistic effects between highly active CoP NPs and NCNHP. And the overpotentials are 140 and 115 mV for the HER under acidic and alkaline conditions to achieve the current density of 10 mA cm^{-2}, and under alkaline conditions, the overpotential is 310 mV for OER. Hollow heterojunctions derived from MOFs can also be used as outstanding bifunctional electrocatalysts. For instance, hollow Co_3S_4@MoS_2 heterostructures[107] and hollow CoS_x@MoS_2 microcubes[108] exhibited outstanding catalytic performance for HER and OER under acidic and alkaline media. The highly efficient electrocatalytic activities benefited from the distinct heterostructures and synergistic effects between Co_3S_4/CoS_x and MoS_2.

Heteroatom-doped carbon nanomaterials are considered to be promising electrocatalysts for the electrocatalytic reaction process. Direct formation of NCNTFs (Figures 8C and 8D) derived from MOFs could be achieved through control of the pyrolysis atmosphere.[103] The as-prepared NCNTFs demonstrated higher $E_{1/2}$, with 0.87 V, for ORR than the 20% Pt/C electrocatalyst with 0.84 V (Figure 8E) and possessed superior stability after 5,000 cycles. In addition, the NCNTFs demonstrated superior electrocatalytic properties for the OER. As shown in Figure 8F, they gave a current density of 10 mA cm^{-2} at 1.60 V (versus RHE), which compared favorably with reported nanocarbon-based catalysts and was lower by ~180 mV than the 20% Pt/C (10 mA cm^{-2}, about 1.78 V). The advantageous traits of NCNTFs, such as hierarchical shells of interconnected crystalline NCNT, optimum graphitic degree, and N-doping level, are the source of the excellent electrocatalytic activity of both ORR and OER.

In summary, MOF-derived hollow nanostructures are promising candidates for applications in electrocatalytic reactions owing to their hierarchical porous structures, accessible active sites, high chemical stability, and good electron conductivity. Electrocatalytic reactions such as ORR, HER, and OER are crucial reaction steps in energy conversion and require catalysts to accelerate their slow kinetics. The performance of the catalyst plays an important role in energy conversion efficiency. Although MOF-derived catalysts have made great achievements in electrocatalytic applications, several problems and challenges still exist, such as device durability, stability, cost issues, catalytic performance, structure-activity relationships, etc. Therefore, the above issues should be considered when designing electrocatalysts.

Energy storage and conversion

MOF-derived hollow nanostructures with extremely porous structures, low density, robust architecture, large surface area, and rich redox reactions of metal ions have verified their unique capabilities and exhibited improved energy storage and conversion performance.[109] Recently, MOF derivatives have been extensively applied in energy storage, especially for LIBs, SIBs, and supercapacitors, and showed high reversible capacity, superior rate, and cycling performance.[109,110]

LIBs and SIBs. Various novel MOF-derived hollow structures, such as CuO,[26,111–113] Co_3O_4,[20,27,114,115] $Zn_xCo_{3-x}O_4$,[116] $CoMnO_4$/Co_3O_4,[117] Co_3O_4/TiO_2,[118] Fe_3O_4,[21] ZnO/$ZnFe_2O_4$/C,[46] $NiCo_2O_4$/NiO,[119] Mn_2O_3,[120] and Co_9S_8,[121] have been reported and used as LIB electrodes. Complex Co_3O_4@$Co_3V_2O_8$

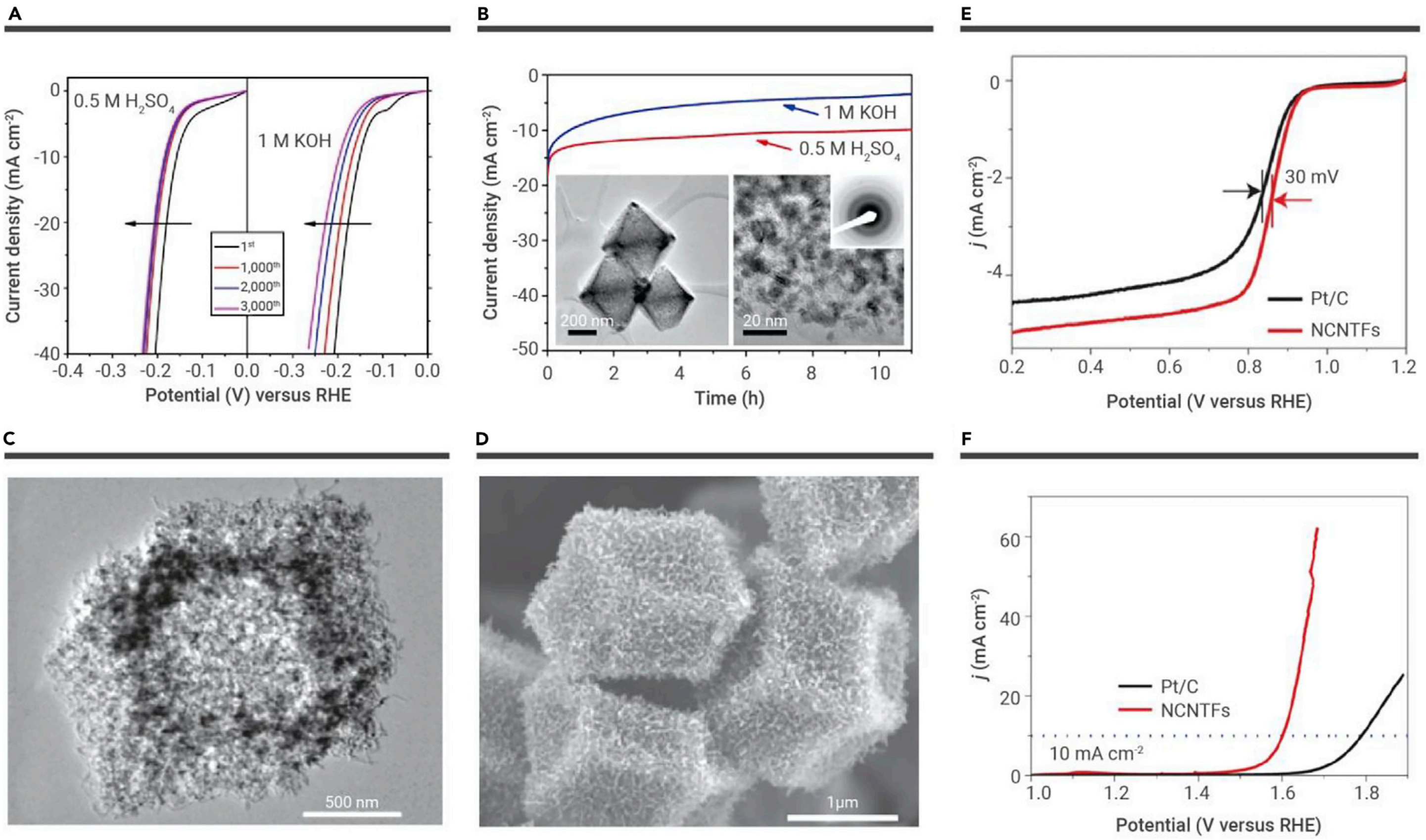

Figure 8. MOF-derived hollow porous materials and their application in electrocatalytic reaction (A and B) HER performance of porous MoC_x nano-octahedrons. (A) Polarization curves after continuous potential sweeps at 50 mV s^{-1} in 0.5 M H_2SO_4 (left) and 1 M KOH (right). (B) Time-dependent current density curves in 0.5 M H_2SO_4 and 1 M KOH (insets: TEM images and SAED pattern after 5,000 potential sweeps in 0.5 M H_2SO_4.[35] (C–F) (C) TEM and (D) SEM images of NCNTFs. LSV curves of Pt/C and NCNTFs for (E) ORR and (F) OER.[103] Copyright Springer Nature.

hollow structures were prepared and applied for LIBs.[122] The experiment revealed that Co_3O_4@$Co_3V_2O_8$ shows superior rate capability, cycling stability, and reversible capacity. Moreover, after a long charge and discharge cycle, the complex hollow structure of Co_3O_4@$Co_3V_2O_8$ is almost unchanged, indicating the stability of the hollow structure in LIBs. In addition to LIBs, MOF-derived hollow structures, i.e., VN-NBs,[123] MHPCS/Se,[124] and Se_xS_y,[125] were also used as electrode materials in Li-S and Li-Se batteries.

The sluggishness of the LiPSs redox reactions is a great challenge in the large-scale practical application of Li-S cells.[126] Aiming at the above problems, Li and co-workers constructed a hollow N-doped porous carbon (Ni-N_5/HNPC) with an optimal Ni-N_5 active moiety, which acted as an ideal host for a sulfur cathode under the guidance of theoretical simulations.[127] First, the Ni-N_x/C structure (x = 3–5) with the highest cathode performance was selected by first-principles calculation. The energy calculation results of the reaction step indicated that the Ni-N_5/C structure is the best cathode candidate. Therefore, they fabricated Ni-N_5/HNPC with Ni-N_5 active sites using MOFs as self-sacrificing templates. As cathode of Li-S batteries, Ni-N_5/HNPC exhibited outstanding rate performance and long-term cycling stability.

Recently, SIBs have been proposed as a potential alternative to LIBs due to the advantage of the high abundance and low cost of the sodium source, high system safety, and wide distribution, as well as storage mechanisms and components similar to those of lithium.[3] Similar to LIBs, MOF-derived hollow metal materials can also be applied as electrodes with remarkable rate capability, cycling stability, and reversible capacity to SIBs.[34,49,128–131] Liu et al. reported on Ni_3S_2/Co_9S_8/N-doped carbon composites via carbonization and sulfurization of binary Ni/Co MOFs (Ni-Co-MOF).[132] Due to the integrated merits of ultrafine Ni_3S_2 and Co_9S_8 NPs (~7 nm), hollow porous structure, and an ultrathin N-doped carbon coating, the final composite material exhibited excellent performance when used as an anode in SIBs. Experiments showed that a reversible specific capacity of 419.9 mAh g^{-1} was achieved after 100 cycles at 0.1 A g^{-1}, and a superior capacity retention rate of 98.6% was achieved. In addition, excellent rate performance was observed: at a current density of 2 A g^{-1}, an average capacity of 323.2 mAh g^{-1} can be reached. The formation of metal sulfide and N-doped carbon layers provides larger capacity and enhanced conductive surface coating. With these virtues, Ni_3S_2/Co_9S_8/N-doped carbon composite materials possess fast sodium storage kinetics and high conductivity, thereby achieving high rate properties.

The construction of composite nanomaterials can effectively alleviate the huge volume expansion/contraction of antimony-based materials when used in sodium-ion batteries, resulting in low cycle life. For instance, Huang and co-workers proposed a NiN-Sb_2Se_3@C composite with a hierarchical nanodot-in-nanofiber structure in which antimony selenide (Sb_2Se_3) nanocrystallites are confined by both 0D and 1D carbon layers.[133] As expected, the NiN-Sb_2Se_3@C composite anode achieved enhanced capacity and exceptional cycle lifespan for over 10,000 cycles at 2.0 A g^{-1} (Figure S5A). Figure S5B demonstrates the process of the structure evolution of micro-Sb_2Se_3, Sb_2Se_3@C, and NiN-Sb_2Se_3@C during electrochemical processes. It is seen that the NiN-Sb_2Se_3@C composite can bear the volume-change-induced strain and avoid aggregation of Sb_2Se_3 NPs. Similarly, the (CoS NP@NHC)@MXene composite manifests distinguished electrochemistry performance when used as electrode material for all of the LIBs, SIBs, and PIBs, which benefit from the synergistic effect of the components (Figure S5C).[134]

Supercapacitors. Supercapacitors have become an ideal choice of energy storage device for their various advantages such as low internal resistance, high power density, and excellent cycle life.[135,136] The surface area and chemical composition of the electrode have a great influence on the properties of the supercapacitor. In this regard, MOF-derived porous hollow structures meet requirements such as affording rich redox chemistry with abundant active sites, inducing the hollow structures for larger capacitance, forming carbon-based nanomaterials for improving surface areas, and enhancing conductivity (Table S3).[110] Until now, hollow-structured metal oxides (MnO_2,[137] NiO/ZnO,[29] Co_3O_4,[138] $Ni_xCo_{3-x}O_4$,[139] Co_3O_4/$NiCo_2O_4$,[27] Co_3O_4/PANI[140]) and metal sulfides (NiCo-LDH/Co_9S_8,[141] Zn-Co-S RDCs,[142] NiS_2/ZnS,[143] $CoS_{1.097}$ NPs,[144] H-NiS_{1-x}/C-50,[145] Co_9S_8@NiO,[146] NiCoMn-S,[147] CoSNP/CoS-NS DSNBs[148]) have seen

widespread use as anode materials for supercapacitors. For instance, $Ni_xCo_{3-x}O_4$ with a hollow structure was used as anode material in supercapacitors and demonstrated outstanding specific capacitance of 2,870.8 F g^{-1} at 1 A g^{-1} and excellent cyclic stability with 81% capacitance retention after 5,000 cycles.[139] In addition, Ho and colleagues fabricated hybrid NiCo-LDH/Co_9S_8 (C/LDH/S) with outstanding performance in supercapacitors.[141] As Figure S5D shows, galvanostatic CD indicated excellent capacitive behavior with highly reversible and rapid reaction kinetics of C/LDH/S. In addition, it exhibited excellent cycle stability and 95.4% retention capacitance after 3,000 cycles. They found that the uniform combination of multiple metal species, the production of heterosulfide-hydroxide, and the arrangement of the hollow structure optimize the catalytic site and enhance conductivity and hydrogen adsorption of NiCo-LDH/Co_9S_8.

Pang and co-workers proposed a general approach for preparing hollow 3D Mxene/MOF composites ($Ti_3C_2T_X$/ZIF-67/CoV_2O_6) by *in situ* growth of MOFs and subsequent ion exchange.[149] $Ti_3C_2T_X$/ZIF-67/CoV_2O_6 overcomes the disadvantage of poor conductivity of traditional MOFs.[150,151] Notably, the Ti_3C_2TX/ZIF-67/CoV_2O_6 electrode demonstrated excellent performance with a high specific capacitance of 253.8 F g^{-1} at 5 A g^{-1} and a high coulombic efficiency of 94.4% after 4,000 GCD cycles at 3 A g^{-1}.

Composite materials can overcome the defects of a single material and integrate the advantages of different nanomaterials, thus facilitating the performance of the target application. Therefore, a composite could obtain neoteric physical and chemical properties that cannot be achieved by a single component.

Environmental applications

Environmental pollution has become a critical issue in human health and environmental protection.[152,153] Benefiting from their tunable configuration, controllable composition, permanent porosity, and larger specific surface area, MOF-derived hollow structures have displayed fascinating physicochemical properties and amassed extensive attention in catalytic degradation of pollutants in our environment. Li and colleagues fabricated ZnO@C-N-Co core-shell nanostructures that used a hollow Zn/Co-ZIF matrix as precursor toward efficient degradation of MO and displayed excellently improved performance and remarkable recyclability.[154] The CeO_2/Au@SiO_2 hollow nanotubes obtained demonstrated high catalytic performance for 4-nitrophenol reduction. The kinetic reaction rate constant (k) of Ce-MOF/Au@SiO_2 (0.71 min^{-1}) is higher than that of Au@SiO_2 (typically 0.1 min^{-1}). Moreover, the catalytic activity was decreased by only ~17.6% after five successive cycles, indicating that the CeO_2/Au@SiO_2 catalyst has superior stability and reusability. The outstanding catalytic performance was attributable to the unique and small size of the Au NPs, as well as the strong synergistic effect between the CeO_2 and the Au NPs.[155] In addition, hollow materials have abundant metal active sites and can also realize the elimination of organic pollutants by exciting free radicals with strong oxidizing properties. For example, the homogeneous bimetallic hollow C-CoM-HNC derived from a MOF achieves the effective removal of RhB by activating persulfate.[156] According to reports, similar studies have used MOF-derived hollow Co_3O_4/carbon as an effective activator of PMPS and achieved the degradation of BPA.[157]

Adsorption technology is widely used to purify environmental pollutants due to its advantages of simple operation, simple regeneration, and large-scale applications.[158–160] The inherent specialties of MOF nanomaterials, including higher surface area, abundant pore structures, and adjustable chemical composition, have been allowing them to serve as prospective candidates for supernormal adsorbents. Yang and co-workers[161] reported MOF-derived porous $Ni_{1-x}Co_xFe_2O_4$ microcubes as adsorbents for efficient removal of nitrophenol. The equilibrium quantity of $Ni_{1-x}Co_xFe_2O_4$ for nitrophenol was 47 mg g^{-1} of ferrite accomplished in 7 min. High specific surface area and mesoporous nature are the keys to the excellent adsorption performance of $Ni_{1-x}Co_xFe_2O_4$ on nitrophenol.[161] Analogously, carbon aerogels bearing a hierarchical structure (Biomass-C@MIL-53-C) were prepared via direct carbonization of kapok fibers assembled with MIL-53 on its surface.[162] As an adsorbent, it demonstrated superior adsorption performance, excellent hydrophobic property, and outstanding cycling stability. Specifically, the adsorption capacities of Biomass-C@MIL-53-C were 35–119.5 times their own weight toward various kinds of oils and organic solvents. Moreover, after eight adsorption-squeezing cycles, it could retain 77.2%–96.7% of its initial adsorption capacity, implying that Biomass-C@MIL-53-C was an outstanding adsorbent for organic pollutant purification.

Advanced oxidation processes (AOPs) are a robust system to degrade refractor organic pollutants in environmental pollution management. MOF-derived SACs have been widely used due to the maximum utilization of metal atoms and the unique electronic properties of metal sites and ultralow metal loads.[163] Mi et al. reported that atomically dispersed Co-SA catalyst derived from MOFs applied to peroxymonosulfate (PMS) activation.[164] DFT calculations revealed that CoN_{2+2} was the definite active site (Figure 9A). $^1O_2$ was the predominant reactive oxygen species, and the proportion was 98.89% (Figures 9B and 9C). The generated $^1O_2$ showed excellent degradation activity for organic pollutants in a wide pH range.

Other applications

In addition to the above-mentioned emerging applications, the unique properties of MOF-derived hollow structures enable their applications in gas storage/separation,[166] gas sensors,[22,31,167–173] extraction of uranium from seawater,[165,174] etc. Xu and co-workers synthesized ZnO/$ZnFe_2O_4$ hollow nanocages and used them as a sensing material for gas sensors. It demonstrated an improved response to acetone (25.8) with a detection limit of 1 ppm.[167] Moreover, MOF-derived Ag/Au HPNS@FO were used for electrochemical As(III) determination and exhibited high sensitivity (922.5 μA ppb^{-1}) and sustained stability.[175]

Recently, our group applied the MOF-derived adsorption-electrocatalyst Fe-N_x-C-R in uranium extraction from seawater and demonstrated considerable results.[165] Real seawater experiments showed that Fe-N_x-C-R has a superior uranium extraction capacity of ~1.2 mg g^{-1} in 24 h. Interestingly, we found that the isolated Fe-N_x site first reduced UO_2^{2+} to UO_2^+, and then oxidized U(V) to U(VI) in the presence of Na^+, finally obtaining the product $Na_2O(UO_3\cdot H_2O)_x$ (Figures 9D and 9E). To our knowledge, the developed system is the first to yield a U(VI) solid product (i.e., $Na_2O(UO_3\cdot H_2O)_x$) in uranium extraction from seawater by an adsorption-electrocatalysis system. We further demonstrated that an amidoxime-functionalized indium-nitrogen-carbon catalyst (In-N_x-C-R) offers a 5-fold higher uranium extraction capacity in seawater compared with our aforementioned Fe-N_x-C-R system. X-ray absorption spectroscopy and *in situ* Raman spectroscopy allowed the relationship between the In-N_x-C-R structure and the adsorption-electrocatalytic mechanism for uranium extraction from seawater to be understood.[174] This provides meaningful clues for the study of uranium extraction from seawater. The adsorption-reduction of U(VI) to U(IV) is an efficient technique for the extraction or preconcentration of U(VI) from aqueous solutions.

In addition to the above applications, MOF-derived porous hollow materials also exhibited excellent performance in photocatalytic and electrocatalytic CO_2 RR. For example, Ma et al.[176] synthesized crystalline MoS_2@TiO_2 nanohybrids with MOFs as precursors through a simple hydrothermal method. The optimal material showed prominent catalytic activity for HER with a high hydrogen production rate of 10,046 μmol h^{-1} g^{-1} under visible light. The catalyst obtained also displayed excellent electrocatalytic activity. The extensive and close contact interface and synergistic effect of different components are regarded as the source of enhanced catalytic activity. Moreover, a novel ZnO@C-N-Co core-shell nanocomposite was also reported as a highly efficient photocatalyst for pollutant photodegradation.[154] The most significant advantage of this material is the recycling performance, which benefits from the assistance of magnetic Co NPs inside the material. Reducing CO_2 toward the generation of valuable chemicals is one of the important strategies to achieve the dual carbon goal.[177,178] Wang et al. presented a carbon-confined indium oxide electrocatalyst for efficient CO_2 RR toward the direct production of formic acid.[179] Experimental results showed that the formate selectivity exceeds 90% in a wide potential window from −0.8 to −1.3 V versus RHE in a liquid-phase flow cell. The high selectivity and activity for CO_2 RR benefited from the carbon protective layer preventing the reductive corrosion of indium oxide and the carbon layer, optimizing the adsorption of reaction intermediates.

CONCLUSION AND OUTLOOK

We have systematically summarized the design strategies of MOFs as precursors and/or soft templates in the fabrication of hollow porous materials, including hollow carbon, metals, metal oxides, metal carbides, metal sulfides, metal hydroxides, and their hybrid composites. Due to various favorable structural features, MOF-derived hollow nanomaterials are considered promising candidates for multifield applications: an enhanced surface area, low density, mesoporous structure,

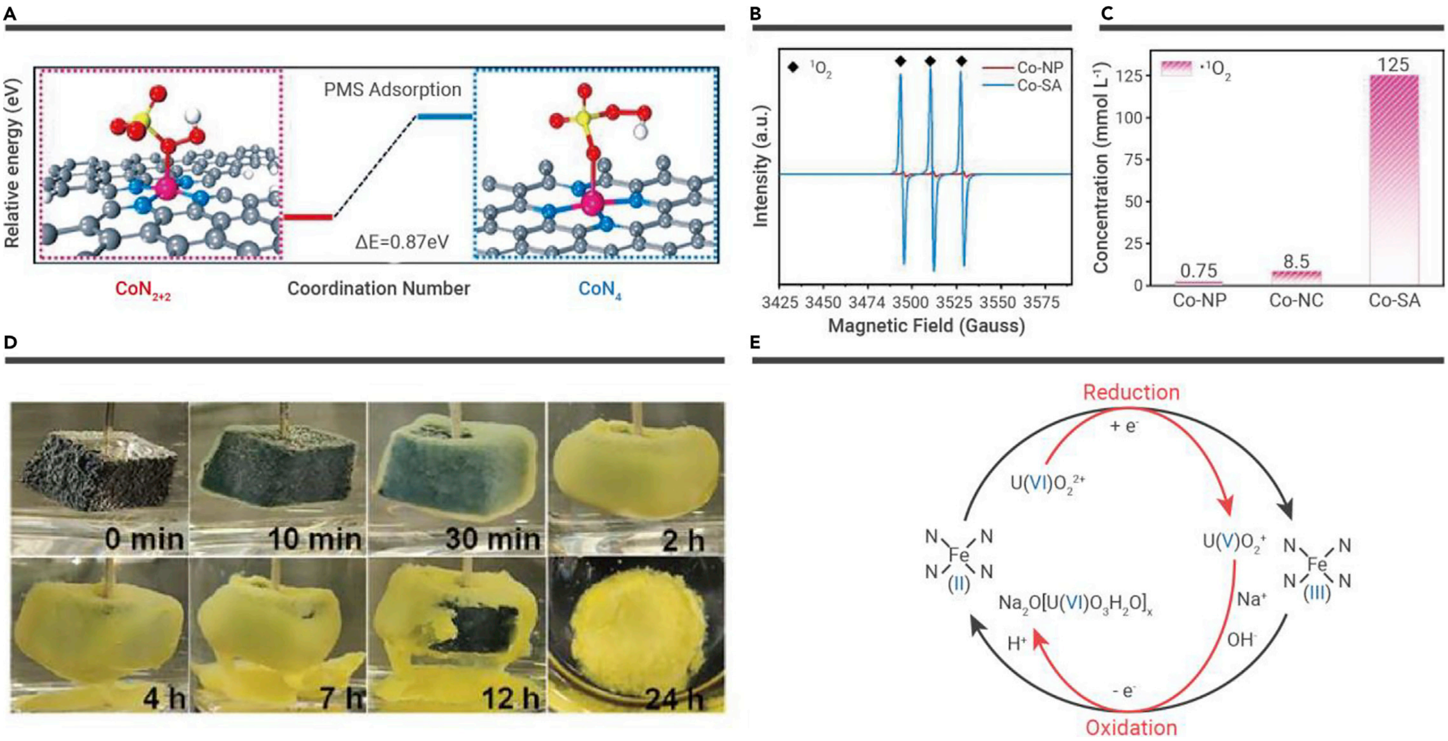

Figure 9. MOF-derived hollow porous materials and their application (A) Calculated free energy evolution of PMS adsorption on the CoN_{2+2} and CoN_4 sites. (B and C) (B) EPR spectra of $^1O_2$ and (C) quantitative determination of $^1O_2$.[164] (D) Photographs of the Fe-N_x-C-R electrode in spiked seawater (initial uranium concentration of ~1,000 ppm) during electrocatalytic extraction. (E) Schematic showing a plausible reaction mechanism for the Fe-N_x-C-R-catalyzed extraction of uranium from seawater.[165] Copyright John Wiley & Sons.

higher loading capacity, and shortened transport distance for mass and charge. Here, the promising applications of MOF-derived hollow structures for chemical catalysis, electrocatalysis, energy storage and conversion, environmental applications, and so on have been summarized.

Despite the intriguing progress and great achievements that have occurred in the synthesis and extensive application of various MOF-derived HPMs, the study is at a burgeoning stage, and more effort is needed to deal with the issues that will be encountered in the future development process. Here, we propose several challenges and research directions that MOF-derived hollow materials may face in future development: (1) more effort should be focused on controllably synthesizing complex structures and regulating components of hollow MOFs and derivatives according to the needs of specific applications; (2) combination with other functional nanomaterials, for example, NPs, clusters, CNTs, and GO, etc., is an economical and facile strategy to regulate the properties of hollow MOFs and their derivatives; (3) the design of hollow MOFs or their derivatives with different sizes and geometrical structures will greatly enrich the storage of HPMs and ultimately affect their internal properties; (4) realizing the large-scale yield of hollow MOFs or derivatives is significant in practical applications; (5) strengthening the deep understanding of structure-activity relationships of hollow material structures is important; particularly, the mechanisms of various MOF-derived hollow materials in electrochemistry have not been thoroughly investigated; and (6) future work should combine experimental and computational methods to explore the interior reaction mechanisms, revealing the influence of structure-property-function and their synergistic interactions.

REFERENCES

1. Furukawa, H., Cordova, K.E., O'Keeffe, M., et al. (2013). The chemistry and applications of metal-organic frameworks. Science ***341***, 974.
2. Kitagawa, S., Kitaura, R., and Noro, S. (2004). Functional porous coordination polymers. Angew. Chem. Int. Ed. Engl. ***43***, 2334–2375.
3. Wang, C., Kim, J., Tang, J., et al. (2020). New strategies for novel MOF-derived carbon materials based on nanoarchitectures. Chem ***6***, 19–40.
4. Yang, H., and Wang, X. (2019). Secondary-component incorporated hollow MOFs and derivatives for catalytic and energy-related applications. Adv. Mater. ***31***, 1800743.
5. Cai, Z.X., Wang, Z.L., Kim, J., et al. (2019). Hollow functional materials derived from metal-organic frameworks: synthetic strategies, conversion mechanisms, and electrochemical applications. Adv. Mater. ***31***, 1804903.
6. Liu, D., Wan, J., Pang, G., et al. (2019). Hollow metal-organic-framework micro/nanostructures and their derivatives: emerging multifunctional materials. Adv. Mater. ***31***, 1803291.
7. Yang, M., Zhang, C.H., Li, N.W., et al. (2022). Design and synthesis of hollow nanostructures for electrochemical water splitting. Adv. Sci. ***9***, 2105135.
8. Azhar, A., Li, Y., Cai, Z., et al. (2019). Nanoarchitectonics: a new materials horizon for prussian blue and its analogues. Bull. Chem. Soc. Jpn. ***92***, 875–904.
9. Cai, G., Yan, P., Zhang, L., et al. (2021). Metal-organic framework-based hierarchically porous materials: synthesis and applications. Chem. Rev. ***121***, 12278–12326.
10. He, L., Li, L., Zhang, L., et al. (2014). ZIF-8 templated fabrication of rhombic dodecahedron-shaped $ZnO@SiO_2$, ZIF-8@SiO_2 yolk-shell and SiO_2 hollow nanoparticles. CrystEngComm ***16***, 6534–6537.
11. Yang, H., Kruger, P.E., Telfer, S.G., et al. (2015). Metal-organic framework nanocrystals as sacrificial templates for hollow and exceptionally porous titania and composite materials. Inorg. Chem. ***54***, 9483–9490.
12. Zhu, W., Xiang, G., Shang, J., et al. (2018). Versatile surface functionalization of metal-organic frameworks through direct metal coordination with a phenolic lipid enables diverse applications. Adv. Funct. Mater. ***28***, 1705274.
13. Li, R., Che, R., Liu, Q., et al. (2017). Hierarchically structured layered-double-hydroxides derived by ZIF-67 for uranium recovery from simulated seawater. J. Hazard Mater. ***338***, 167–176.
14. Jiang, Z., Li, Z., Qin, Z., et al. (2013). LDH nanocages synthesized with MOF templates and their high performance as supercapacitors. Nanoscale ***5***, 11770–11775.
15. Du, Y., Li, G., Chen, M., et al. (2019). Hollow nickel-cobalt-manganese hydroxide polyhedra via MOF templates for high-performance quasi-solid-state supercapacitor. Chem. Eng. J. ***378***, 122210.
16. Wang, J., Li, S., Lin, R., et al. (2019). MOF-derived hollow β-FeOOH polyhedra anchored with α-$Ni(OH)_2$ nanosheets as efficient electrocatalysts for oxygen evolution. Electrochim. Acta ***301***, 258–266.
17. Bai, X., Liu, J., Liu, Q., et al. (2017). In-situ fabrication of MOF-derived Co-Co layered double hydroxide hollow nanocages/graphene composite: a novel electrode material with superior electrochemical performance. Chemistry ***23***, 14839–14847.
18. Zhang, J., Yu, L., Chen, Y., et al. (2020). Designed formation of double-shelled Ni-Fe layered-double-hydroxide nanocages for efficient oxygen evolution reaction. Adv. Mater. ***32***, 1906432.
19. Wang, W., Yan, H., Anand, U., et al. (2021). Visualizing the conversion of metal-organic framework nanoparticles into hollow layered double hydroxide nanocages. J. Am. Chem. Soc. ***143***, 1854–1862.
20. Tian, D., Zhou, X.L., Zhang, Y.H., et al. (2015). MOF-derived porous Co_3O_4 hollow tetrahedra with excellent performance as anode materials for lithium-ion batteries. Inorg. Chem. ***54***, 8159–8161.
21. Guo, W., Sun, W., Lv, L.P., et al. (2017). Microwave-assisted morphology evolution of Fe-based metal-organic frameworks and their derived Fe_2O_3 nanostructures for Li-ion storage. ACS Nano ***11***, 4198–4205.

22. Li, W., Wu, X., Han, N., et al. (2016). MOF-derived hierarchical hollow ZnO nanocages with enhanced low-concentration VOCs gas-sensing performance. Sensor. Actuator. B Chem. ***225***, 158–166.
23. Chen, G., Guo, Z., Zhao, W., et al. (2017). Design of porous/hollow structured ceria by partial thermal decomposition of Ce-MOF and selective etching. ACS Appl. Mater. Interfaces ***9***, 39594–39601.
24. Tao, K., Han, X., Yin, Q., et al. (2017). Metal-organic frameworks-derived porous In_2O_3 hollow nanorod for high-performance ethanol gas sensor. ChemistrySelect ***2***, 10918–10925.
25. Cai, Y., Fang, G., Zhou, J., et al. (2018). Metal-organic framework-derived porous shuttle-like vanadium oxides for sodium-ion battery application. Nano Res. ***11***, 449–463.
26. Guo, W., Sun, W., and Wang, Y. (2015). Multilayer CuO@NiO hollow spheres: microwave-assisted metal-organic-framework derivation and highly reversible structure-matched stepwise lithium storage. ACS Nano ***9***, 11462–11471.
27. Hu, H., Guan, B., Xia, B., et al. (2015). Designed formation of $Co_3O_4/NiCo_2O_4$ double-shelled nanocages with enhanced pseudocapacitive and electrocatalytic properties. J. Am. Chem. Soc. ***137***, 5590–5595.
28. Antony, R.P., Satpati, A.K., Bhattacharyya, K., et al. (2016). MOF derived nonstoichiometric $NixCo_{3-x}O_{4-y}$ nanocage for superior electrocatalytic oxygen evolution. Adv. Mater. Interfaces ***3***, 1600632.
29. Li, G.C., Liu, P.F., Liu, R., et al. (2016). MOF-derived hierarchical double-shelled NiO/ZnO hollow spheres for high-performance supercapacitors. Dalton Trans. ***45***, 13311–13316.
30. Zhen, S.Y., Wu, H.T., Wang, Y., et al. (2019). Metal-organic framework derived hollow porous $CuO-CuCo_2O_4$ dodecahedrons as a cathode catalyst for LiO_2 batteries. RSC Adv. ***9***, 16288–16295.
31. Qu, F., Jiang, H., and Yang, M. (2016). Designed formation through a metal organic framework route of $ZnO/ZnCo_2O_4$ hollow core-shell nanocages with enhanced gas sensing properties. Nanoscale ***8***, 16349–16356.
32. Chen, X., Li, J.J., Chen, X., et al. (2018). MOF-templated approach for hollow NiO_x/Co_3O_4 catalysts: enhanced light-driven thermocatalytic degradation of toluene. ACS Appl. Nano Mater. ***1***, 2971–2981.
33. Guan, B.Y., Kushima, A., Yu, L., et al. (2017). Coordination polymers derived general synthesis of multishelled mixed metal-oxide particles for hybrid supercapacitors. Adv. Mater. ***29***, 1605902.
34. Zhang, W., Jiang, X., Zhao, Y., et al. (2017). Hollow carbon nanobubbles: monocrystalline MOF nanobubbles and their pyrolysis. Chem. Sci. ***8***, 3538–3546.
35. Wu, H.B., Xia, B.Y., Yu, L., et al. (2015). Porous molybdenum carbide nano-octahedrons synthesized via confined carburization in metal-organic frameworks for efficient hydrogen production. Nat. Commun. ***6***, 6512.
36. Wang, J., Huang, Z., Liu, W., et al. (2017). Design of N-coordinated dual-metal sites: a stable and active Pt-free catalyst for acidic oxygen reduction reaction. J. Am. Chem. Soc. ***139***, 17281–17284.
37. Hu, M., Ju, Y., Liang, K., et al. (2016). Void engineering in metal-organic frameworks via synergistic etching and surface functionalization. Adv. Funct. Mater. ***26***, 5827–5834.
38. Yang, H., Bradley, S.J., Chan, A., et al. (2016). Catalytically active bimetallic nanoparticles supported on porous carbon capsules derived from metal–organic framework composites. J. Am. Chem. Soc. ***138***, 11872–11881.
39. Yang, S., Peng, L., Huang, P., et al. (2016). Nitrogen, phosphorus, and sulfur Co-doped hollow carbon shell as superior metal-free catalyst for selective oxidation of aromatic alkanes. Angew. Chem. ***128***, 4084–4088.
40. Liu, S., Wang, Z., Zhou, S., et al. (2017). Metal-organic-framework-derived hybrid carbon nanocages as a bifunctional electrocatalyst for oxygen reduction and evolution. Adv. Mater. ***29***, 1700874.
41. Yang, H., Bradley, S.J., Wu, X., et al. (2018). General synthetic strategy for libraries of supported multicomponent metal nanoparticles. ACS Nano ***12***, 4594–4604.
42. Yang, H., Chen, X., Chen, W.T., et al. (2019). Tunable synthesis of hollow metal–nitrogen-carbon capsules for efficient oxygen reduction catalysis in proton exchange membrane fuel cells. ACS Nano ***13***, 8087–8098.
43. Chen, Y., Ji, S., Zhao, S., et al. (2018). Enhanced oxygen reduction with single-atomic-site iron catalysts for a zinc-air battery and hydrogen-air fuel cell. Nat. Commun. ***9***, 5422.
44. Pan, Y., Sun, K., Liu, S., et al. (2018). Core-shell ZIF-8@ZIF-67-derived CoP nanoparticle-embedded N-doped carbon nanotube hollow polyhedron for efficient overall water splitting. J. Am. Chem. Soc. ***140***, 2610–2618.
45. Jagadeesh, R.V., Murugesan, K., Alshammari, A.S., et al. (2017). MOF-derived cobalt nanoparticles catalyze a general synthesis of amines. Science ***358***, 326–332.
46. Zou, F., Hu, X., Li, Z., et al. (2014). MOF-derived porous $ZnO/ZnFe_2O_4/C$ octahedra with hollow interiors for high-rate lithium-ion batteries. Adv. Mater. ***26***, 6622–6628.
47. He, P., Yu, X.Y., and Lou, X.W.D. (2017). Carbon-incorporated nickel-cobalt mixed metal phosphide nanoboxes with enhanced electrocatalytic activity for oxygen evolution. Angew. Chem. Int. Ed. Engl. ***56***, 3897–3900.
48. Liu, X., Liu, Y., and Fan, L.Z. (2017). MOF-derived $CoSe_2$ microspheres with hollow interiors as high-performance electrocatalysts for the enhanced oxygen evolution reaction. J. Mater. Chem. ***5***, 15310–15314.
49. Zou, F., Chen, Y.M., Liu, K., et al. (2016). Metal organic frameworks derived hierarchical hollow NiO/Ni/graphene composites for lithium and sodium storage. ACS Nano ***10***, 377–386.
50. Yu, L., Yang, J.F., and Lou, X.W.D. (2016). Formation of CoS_2 nanobubble hollow prisms for highly reversible lithium storage. Angew. Chem. Int. Ed. Engl. ***128***, 13620–13624.
51. Wang, S., Fan, Y., Teng, J., et al. (2016). Nanoreactor based on macroporous single crystals of metal-organic framework. Small ***12***, 5702–5709.
52. Shen, K., Zhang, L., Chen, X., et al. (2018). Ordered macro-microporous metal-organic framework single crystals. Science ***359***, 206–210.
53. Song, Y., Chen, Y., Wu, J., et al. (2017). Hollow metal organic frameworks-derived porous ZnO/C nanocages as anode materials for lithium-ion batteries. J. Alloys Compd. ***694***, 1246–1253.
54. Lv, Z., Fan, Q., Xie, Y., et al. (2019). MOFs-derived magnetic chestnut shell-like hollow sphere NiO/Ni@C composites and their removal performance for arsenic(V). Chem. Eng. J. ***362***, 413–421.
55. Wu, Y., Chen, H., Wang, J., et al. (2020). Metal-organic framework-templated hollow Co_3O_4/C with controllable oxygen vacancies for efficient oxygen evolution reaction. ChemNanoMat ***6***, 107–112.
56. Zhang, Z., Chen, Y., He, S., et al. (2014). Hierarchical Zn/Ni-MOF-2 nanosheet-assembled hollow nanocubes for multicomponent catalytic reactions. Angew. Chem. Int. Ed. Engl. ***53***, 12517–12521.
57. Liu, Y., Zhang, W., Li, S., et al. (2014). Designable yolk-shell nanoparticle@MOF petalous heterostructures. Chem. Mater. ***26***, 1119–1125.
58. Kuo, C.H., Tang, Y., Chou, L.Y., et al. (2012). Yolk-shell nanocrystal@ZIF-8 nanostructures for gas-phase heterogeneous catalysis with selectivity control. J. Am. Chem. Soc. ***134***, 14345–14348.
59. Guo, Y., Feng, L., Wu, C., et al. (2020). Confined pyrolysis transformation of ZIF-8 to hierarchically ordered porous Zn-NC nanoreactor for efficient CO_2 photoconversion under mild conditions. J. Catal. ***390***, 213–223.
60. Liu, W., Huang, J., Yang, Q., et al. (2017). Multi-shelled hollow metal-organic frameworks. Angew. Chem. Int. Ed. Engl. ***56***, 5512–5516.
61. Lee, J., Kwak, J.H., and Choe, W. (2017). Evolution of form in metal-organic frameworks. Nat. Commun. ***8***, 14070.
62. Liu, X.Y., Zhang, F., Goh, T.W., et al. (2018). Using a multi-shelled hollow metal-organic framework as a host to switch the guest-to-host and guest-to-guest interactions. Angew. Chem. Int. Ed. Engl. ***57***, 2110–2114.
63. Guo, J., Qin, Y., Zhu, Y., et al. (2021). Metal-organic frameworks as catalytic selectivity regulators for organic transformations. Chem. Soc. Rev. ***50***, 5366–5396.
64. Yang, Y., Wang, F., Yang, Q., et al. (2014). Hollow metal-organic framework nanospheres via emulsion-based interfacial synthesis and their application in size-selective catalysis. ACS Appl. Mater. Interfaces ***6***, 18163–18171.
65. Lin, X., Wang, S., Tu, W., et al. (2019). MOF-derived hierarchical hollow spheres composed of carbon-confined Ni nanoparticles for efficient CO_2 methanation. Catal. Sci. Technol. ***9***, 731–738.
66. Yang, Q., Yang, C.C., Lin, C.H., et al. (2019). Metal-organic-framework-derived hollow N-doped porous carbon with ultrahigh concentrations of single Zn atoms for efficient carbon dioxide conversion. Angew. Chem. Int. Ed. Engl. ***131***, 3549–3553.
67. Wan, M., Zhang, X., Li, M., et al. (2017). Hollow Pd/MOF nanosphere with double shells as multifunctional catalyst for hydrogenation reaction. Small ***13***, 1701395.
68. Zhang, Z., Chen, Y., Xu, X., et al. (2014). Well-defined metal-organic framework hollow nanocages. Angew. Chem. Int. Ed. Engl. ***53***, 429–433.
69. Fang, X., Shi, Y., Wu, K., et al. (2017). Upgrading of palmitic acid over MOF catalysts in supercritical fluid of n-hexane. RSC Adv. ***7***, 40581–40590.
70. Yang, J., Zhang, F., Lu, H., et al. (2015). Hollow Zn/Co ZIF particles derived from core-shell ZIF-67@ZIF-8 as selective catalyst for the semi-hydrogenation of acetylene. Angew. Chem. ***127***, 11039–11043.
71. Zhao, J., Tang, Z., Dong, F., et al. (2019). Controlled porous hollow Co_3O_4 polyhedral nanocages derived from metal-organic frameworks (MOFs) for toluene catalytic oxidation. Mol. Catal. ***463***, 77–86.
72. Chen, H., Shen, K., Mao, Q., et al. (2018). Nanoreactor of MOF-derived yolk-shell Co@C-N: precisely controllable structure and enhanced catalytic activity. ACS Catal. ***8***, 1417–1426.
73. Fang, R., Luque, R., and Li, Y. (2016). Selective aerobic oxidation of biomass-derived HMF to 2, 5-diformylfuran using a MOF-derived magnetic hollow Fe-Co nanocatalyst. Green Chem. ***18***, 3152–3157.
74. Sun, K.K., Chen, S.J., Li, Z.L., et al. (2019). Synthesis of a ZIF-derived hollow yolk-shell Co@CN catalyst for the oxidative esterification of 5-hydroxymethylfurfural. Green Chem. ***21***, 1602–1608.
75. Zhou, M., Wang, H.L., and Guo, S. (2016). Towards high-efficiency nanoelectrocatalysts for oxygen reduction through engineering advanced carbon nanomaterials. Chem. Soc. Rev. ***45***, 1273–1307.
76. Zhong, L., Huang, Q., Ding, J., et al. (2021). Abundant $Co-N_x$ sites onto hollow MOF-Derived nitrogen-doped carbon materials for enhanced oxygen reduction. J. Power Sources ***492***, 229632.
77. Zhao, S., Yin, H., Du, L., et al. (2014). Carbonized nanoscale metal-organic frameworks as high performance electrocatalyst for oxygen reduction reaction. ACS Nano ***8***, 12660–12668.
78. Xia, W., Zou, R., An, L., et al. (2015). A metal-organic framework route to in situ encapsulation of $Co@Co_3O_4@C$ core@bishell nanoparticles into a highly ordered porous carbon matrix for oxygen reduction. Energy Environ. Sci. ***8***, 568–576.
79. Wan, X., Liu, X., Li, Y., et al. (2019). Fe-N-C electrocatalyst with dense active sites and efficient mass transport for high-performance proton exchange membrane fuel cells. Nat. Catal. ***2***, 259–268.
80. Yang, H., Wang, X., Zheng, T., et al. (2021). CrN-encapsulated hollow Cr-NC capsules boosting oxygen reduction catalysis in PEMFC. CCS Chem. ***3***, 208–218.

81. Zhu, M., Zhao, C., Liu, X., et al. (2021). Single atomic cerium sites with a high coordination number for efficient oxygen reduction in proton-exchange membrane fuel cells. ACS Catal. ***11***, 3923–3929.
82. Wei, X., Zheng, D., Zhao, M., et al. (2020). Cross-linked polyphosphazene hollow nanosphere-derived N/P-doped porous carbon with single nonprecious metal atoms for the oxygen reduction reaction. Angew. Chem. Int. Ed. Engl. ***132***, 14747–14754.
83. Chai, L., Zhang, L., Wang, X., et al. (2019). Bottom-up synthesis of MOF-derived hollow N-doped carbon materials for enhanced ORR performance. Carbon ***146***, 248–256.
84. Kong, F., Cui, X., Huang, Y., et al. (2022). N-doped carbon electrocatalyst: marked ORR activity in acidic media without the contribution by metal sites? Angew. Chem. Int. Ed. Engl. ***61***, e202116290.
85. Zhang, S.L., Guan, B.Y., and Lou, X.W.D. (2019). Co-Fe alloy/N-doped carbon hollow spheres derived from dual metal-organic frameworks for enhanced electrocatalytic oxygen reduction. Small ***15***, 1805324.
86. Xiao, J., Zhao, C., Hu, C., et al. (2017). Pudding-typed cobalt sulfides/nitrogen and sulfur dual-doped hollow carbon spheres as a highly efficient and stable oxygen reduction electrocatalyst. J. Power Sources ***348***, 183–192.
87. Yang, L., Zeng, Y., Tang, X., et al. (2018). Self-sacrificial template synthesis of a nitrogen-doped microstructured carbon tube as electrocatalyst for oxygen reduction. Chemelectrochem ***5***, 3731–3740.
88. Deng, Y., Chi, B., Tian, X., et al. (2019). g-C_3N_4 promoted MOF derived hollow carbon nanopolyhedra doped with high density/fraction of single Fe atoms as an ultra-high performance non-precious catalyst towards acidic ORR and PEM fuel cells. J. Mater. Chem. ***7***, 5020–5030.
89. Yang, H., Liu, Y., Liu, X., et al. (2022). Large-scale synthesis of N-doped carbon capsules supporting atomically dispersed iron for efficient oxygen reduction reaction electrocatalysis. eScience ***2***, 227–234.
90. Wan, X., Liu, X., and Shui, J. (2020). Stability of PGM-free fuel cell catalysts: degradation mechanisms and mitigation strategies. Prog. Nat. Sci. Mater. Int. ***30***, 721–731.
91. Luo, E., Chu, Y., Liu, J., et al. (2021). Pyrolyzed M-N_x catalysts for oxygen reduction reaction: progress and prospects. Energy Environ. Sci. ***14***, 2158–2185.
92. Zhao, C.X., Li, B.Q., Liu, J.N., et al. (2021). Intrinsic electrocatalytic activity regulation of M-N-C single-atom catalysts for the oxygen reduction reaction. Angew. Chem. Int. Ed. Engl. ***60***, 4448–4463.
93. Chen, Y.Z., Wang, C., Wu, Z.Y., et al. (2015). From bimetallic metal-organic framework to porous carbon: high surface area and multicomponent active dopants for excellent electrocatalysis. Adv. Mater. ***27***, 5010–5016.
94. Meng, Z., Chen, N., Cai, S., et al. (2021). Recent advances of hierarchically porous bifunctional oxygen electrocatalysts derived from metal-organic frameworks for Zn-air batteries. Mater. Chem. Front. ***5***, 2649–2667.
95. Burke, M.S., Kast, M.G., Trotochaud, L., et al. (2015). Cobalt-iron (oxy) hydroxide oxygen evolution electrocatalysts: the role of structure and composition on activity, stability, and mechanism. J. Am. Chem. Soc. ***137***, 3638–3648.
96. Friebel, D., Louie, M.W., Bajdich, M., et al. (2015). Identification of highly active Fe sites in (Ni, Fe) OOH for electrocatalytic water splitting. J. Am. Chem. Soc. ***137***, 1305–1313.
97. Zhang, X., Wu, Y., Sun, Y., et al. (2019). CoFeP hollow cube as advanced electrocatalyst for water oxidation. Inorg. Chem. Front. ***6***, 604–611.
98. Kang, B.K., Woo, M.H., Lee, J., et al. (2017). Mesoporous Ni-Fe oxide multi-composite hollow nanocages for efficient electrocatalytic water oxidation reactions. J. Mater. Chem. ***5***, 4320–4324.
99. Cai, G., Zhang, W., Jiao, L., et al. (2017). Template-directed growth of well-aligned MOF arrays and derived self-supporting electrodes for water splitting. Chem ***2***, 791–802.
100. Han, L., Yu, X.Y., and Lou, X.W.D. (2016). Formation of prussian-blue-analog nanocages via a direct etching method and their conversion into ni–co-mixed oxide for enhanced oxygen evolution. Adv. Mater. ***28***, 4601–4605.
101. Xu, H., Cao, J., Shan, C., et al. (2018). MOF-derived hollow CoS decorated with CeO_x nanoparticles for boosting oxygen evolution reaction electrocatalysis. Angew. Chem. ***130***, 8790–8794.
102. Zheng, Y., Jiao, Y., Jaroniec, M., et al. (2015). Advancing the electrochemistry of the hydrogen-evolution reaction through combining experiment and theory. Angew. Chem. Int. Ed. Engl. ***54***, 52–65.
103. Xia, B.Y., Yan, Y., Li, N., et al. (2016). A metal-organic framework-derived bifunctional oxygen electrocatalyst. Nat. Energy ***1***, 15006.
104. Xu, X., Nosheen, F., and Wang, X. (2016). Ni-decorated molybdenum carbide hollow structure derived from carbon-coated metal-organic framework for electrocatalytic hydrogen evolution reaction. Chem. Mater. ***28***, 6313–6320.
105. Zhang, S.L., Lu, X.F., Wu, Z.P., et al. (2021). Engineering platinum-cobalt nano-alloys in porous nitrogen-doped carbon nanotubes for highly efficient electrocatalytic hydrogen evolution. Angew. Chem. Int. Ed. Engl. ***60***, 19068–19073.
106. Huang, Z.F., Song, J., Li, K., et al. (2016). Hollow cobalt-based bimetallic sulfide polyhedra for efficient all-pH-value electrochemical and photocatalytic hydrogen evolution. J. Am. Chem. Soc. ***138***, 1359–1365.
107. Guo, Y., Tang, J., Qian, H., et al. (2017). One-pot synthesis of zeolitic imidazolate framework 67-derived hollow Co_3S_4@MoS_2 heterostructures as efficient bifunctional catalysts. Chem. Mater. ***29***, 5566–5573.
108. Yang, L., Zhang, L., Xu, G., et al. (2018). Metal-organic-framework-derived hollow CoS_x@MoS_2 microcubes as superior bifunctional electrocatalysts for hydrogen evolution and oxygen evolution reactions. ACS Sustain. Chem. Eng. ***6***, 12961–12968.
109. Hu, W., Zheng, M., Xu, B., et al. (2021). Design of hollow carbon-based materials derived from metal-organic frameworks for electrocatalysis and electrochemical energy storage. J. Mater. Chem. ***9***, 3880–3917.
110. Xu, B., Zhang, H., Mei, H., et al. (2020). Recent progress in metal-organic framework-based supercapacitor electrode materials. Coord. Chem. Rev. ***420***, 213438.
111. Wu, R., Qian, X., Yu, F., et al. (2013). MOF-templated formation of porous CuO hollow octahedra for lithium-ion battery anode materials. J. Mater. Chem. ***1***, 11126.
112. Hu, L., Huang, Y., Zhang, F., et al. (2013). CuO/Cu_2O composite hollow polyhedrons fabricated from metal-organic framework templates for lithium-ion battery anodes with a long cycling life. Nanoscale ***5***, 4186–4190.
113. Ramaraju, B., Li, C.H., Prakash, S., et al. (2016). Metal-organic framework derived hollow polyhedron metal oxide posited graphene oxide for energy storage applications. Chem. Commun. ***52***, 946–949.
114. Shao, J., Wan, Z., Liu, H., et al. (2014). Metal organic frameworks-derived Co_3O_4 hollow dodecahedrons with controllable interiors as outstanding anodes for Li storage. J. Mater. Chem. ***2***, 12194–12200.
115. Han, Y., Zhao, M., Dong, L., et al. (2015). MOF-derived porous hollow Co_3O_4 parallelepipeds for building high-performance Li-ion batteries. J. Mater. Chem. ***3***, 22542–22546.
116. Wu, R., Qian, X., Zhou, K., et al. (2014). Porous spinel $Zn_xCo_{3-x}O_4$ hollow polyhedra templated for high-rate lithium-ion batteries. ACS Nano ***8***, 6297–6303.
117. Chen, Y., Wang, Y., Shen, X., et al. (2018). Cyanide-metal framework derived $CoMoO_4$/Co_3O_4 hollow porous octahedrons as advanced anodes for high performance lithium ion batteries. J. Mater. Chem. ***6***, 1048–1056.
118. Xu, W., Cui, X., Xie, Z., et al. (2016). Integrated Co_3O_4/TiO_2 composite hollow polyhedrons prepared via cation-exchange metal-organic framework for superior lithium-ion batteries. Electrochim. Acta ***222***, 1021–1028.
119. Sun, C., Yang, J., Rui, X., et al. (2015). MOF-directed templating synthesis of a porous multicomponent dodecahedron with hollow interiors for enhanced lithium-ion battery anodes. J. Mater. Chem. ***3***, 8483–8488.
120. Zheng, F., Xu, S., Yin, Z., et al. (2016). Facile synthesis of MOF-derived Mn_2O_3 hollow microspheres as anode materials for lithium-ion batteries. RSC Adv. ***6***, 93532–93538.
121. Liu, J., Wu, C., Xiao, D., et al. (2016). MOF-derived hollow Co_9S_8 nanoparticles embedded in graphitic carbon nanocages with superior Li-ion storage. Small ***12***, 2354–2364.
122. Lu, Y., Yu, L., Wu, M., et al. (2018). Construction of complex Co_3O_4@$Co_3V_2O_8$ hollow structures from metal-organic frameworks with enhanced lithium storage properties. Adv. Mater. ***30***, 1702875.
123. Ma, L., Yuan, H., Zhang, W., et al. (2017). Porous-shell vanadium nitride nanobubbles with ultrahigh areal sulfur loading for high-capacity and long-life lithium-sulfur batteries. Nano Lett. ***17***, 7839–7846.
124. Liu, T., Dai, C., Jia, M., et al. (2016). Selenium embedded in MOFs-derived hollow hierarchical porous carbon spheres for advanced lithium-selenium batteries. ACS Appl. Mater. Interfaces ***8***, 16063–16070.
125. Abouimrane, A., Dambournet, D., Chapman, K.W., et al. (2012). A new class of lithium and sodium rechargeable batteries based on selenium and selenium-sulfur as a positive electrode. J. Am. Chem. Soc. ***134***, 4505–4508.
126. Li, S., Zhang, W., Zheng, J., et al. (2021). Inhibition of polysulfide shuttles in Li-S batteries: modified separators and solid-state electrolytes. Adv. Energy Mater. ***11***, 2000779.
127. Zhang, S., Ao, X., Huang, J., et al. (2021). Isolated single-atom Ni-N_5 catalytic site in hollow porous carbon capsules for efficient lithium-sulfur batteries. Nano Lett. ***21***, 9691–9698.
128. Kaneti, Y.V., Zhang, J., He, Y.B., et al. (2017). Fabrication of an MOF-derived heteroatom-doped Co/CoO/carbon hybrid with superior sodium storage performance for sodium-ion batteries. J. Mater. Chem. ***5***, 15356–15366.
129. Zhang, X., Qin, W., Li, D., et al. (2015). Metal-organic framework derived porous CuO/Cu_2O composite hollow octahedrons as high performance anode materials for sodium ion batteries. Chem. Commun. ***51***, 16413–16416.
130. Guo, Y., Zhu, Y., Yuan, C., et al. (2017). $MgFe_2O_4$ hollow microboxes derived from metal-organic-frameworks as anode material for sodium-ion batteries. Mater. Lett. ***199***, 101–104.
131. Zhao, W., Wang, X., Ma, X., et al. (2021). In situ tailoring bimetallic-organic framework-derived yolk-shell NiS_2/CuS hollow microspheres: an extraordinary kinetically pseudocapacitive nanoreactor for an effective sodium-ion storage anode. J. Mater. Chem. ***9***, 15807–15819.
132. Liu, X., Zou, F., Liu, K., et al. (2017). A binary metal organic framework derived hierarchical hollow Ni_3S_2/Co_9S_8/N-doped carbon composite with superior sodium storage performance. J. Mater. Chem. ***5***, 11781–11787.
133. Li, Q., Zhang, W., Peng, J., et al. (2022). Nanodot-in-nanofiber structured carbon-confined Sb_2Se_3 crystallites for fast and durable sodium storage. Adv. Funct. Mater. 2112776.
134. Yao, L., Gu, Q., and Yu, X. (2021). Three-dimensional MOFs@MXene aerogel composite derived MXene threaded hollow carbon confined CoS nanoparticles toward advanced alkali-ion batteries. ACS Nano ***15***, 3228–3240.
135. Chen, L.F., Yu, Z.Y., Wang, J.J., et al. (2015). Metal-like fluorine-doped β-FeOOH nanorods grown on carbon cloth for scalable high-performance supercapacitors. Nano Energy ***11***, 119–128.
136. Grote, F., Yu, Z.Y., Wang, J.L., et al. (2015). Self-stacked reduced graphene oxide nanosheets coated with cobalt-nickel hydroxide by one-step electrochemical deposition toward flexible electrochromic supercapacitors. Small ***11***, 4666–4672.
137. Maiti, S., Pramanik, A., and Mahanty, S. (2015). Influence of imidazolium-based ionic liquid electrolytes on the performance of nano-structured MnO_2 hollow spheres as electrochemical supercapacitor. RSC Adv. ***5***, 41617–41626.

138. Li, H., Yue, F., Yang, C., et al. (2016). Porous nanotubes derived from a metal-organic framework as high-performance supercapacitor electrodes. Ceram. Int. **42**, 3121–3129.
139. Jayakumar, A., Antony, R.P., Wang, R., et al. (2017). MOF-derived hollow cage $NixCo_{3-x}O_4$ and their synergy with graphene for outstanding supercapacitors. Small **13**, 1603102.
140. Ren, X., Fan, H., Ma, J., et al. (2018). Hierarchical Co_3O_4/PANI hollow nanocages: synthesis and application for electrode materials of supercapacitors. Appl. Surf. Sci. **441**, 194–203.
141. Yilmaz, G., Yam, K.M., Zhang, C., et al. (2017). In situ transformation of MOFs into layered double hydroxide embedded metal sulfides for improved electrocatalytic and supercapacitive performance. Adv. Mater. **29**, 1606814.
142. Zhang, P., Guan, B.Y., Yu, L., et al. (2017). Formation of double-shelled zinc-cobalt sulfide dodecahedral cages from bimetallic zeolitic imidazolate frameworks for hybrid supercapacitors. Angew. Chem. Int. Ed. Engl. **129**, 7247–7251.
143. Li, G.C., Liu, M., Wu, M.K., et al. (2016). MOF-derived self-sacrificing route to hollow NiS_2/ZnS nanospheres for high performance supercapacitors. RSC Adv. **6**, 103517–103522.
144. Cao, F., Zhao, M., Yu, Y., et al. (2016). Synthesis of two-dimensional $CoS_{1.097}$/nitrogen-doped carbon nanocomposites using metal-organic framework nanosheets as precursors for supercapacitor application. J. Am. Chem. Soc. **138**, 6924–6927.
145. Huang, C., Gao, A., Yi, F., et al. (2021). Metal organic framework derived hollow NiS@C with S-vacancies to boost high-performance supercapacitors. Chem. Eng. J. **419**, 129643.
146. Wang, J., Huang, Y., Han, X., et al. (2021). Construction of hierarchical Co_9S_8@NiO synergistic microstructure for high-performance asymmetric supercapacitor. J. Colloid Interface Sci. **603**, 440–449.
147. Kang, C., Ma, L., Chen, Y., et al. (2022). Metal-organic framework derived hollow rod-like NiCoMn ternary metal sulfide for high-performance asymmetric supercapacitors. Chem. Eng. J. **427**, 131003.
148. Hu, H., Guan, B., and Lou, X. (2016). Construction of complex CoS hollow structures with enhanced electrochemical properties for hybrid supercapacitors. Chem **1**, 102–113.
149. Liu, C., Bai, Y., Li, W., et al. (2022). In situ growth of three-dimensional MXene/Metal-organic framework composites for high-performance supercapacitors. Angew. Chem. Int. Ed. Engl. **61**, e202116282.
150. Sheberla, D., Bachman, J.C., Elias, J.S., et al. (2017). Conductive MOF electrodes for stable supercapacitors with high areal capacitance. Nat. Mater. **16**, 220–224.
151. Xiao, X., Zou, L., Pang, H., et al. (2020). Synthesis of micro/nanoscaled metal-organic frameworks and their direct electrochemical applications. Chem. Soc. Rev. **49**, 301–331.
152. Cai, Y., Zhang, Y., Lv, Z., et al. (2022). Highly efficient uranium extraction by a piezo catalytic reduction-oxidation process. Appl. Catal. B Environ. **310**, 121343.
153. Chen, T., Yu, K., Dong, C., et al. (2022). Advanced photocatalysts for uranium extraction: elaborate design and future perspectives. Coord. Chem. Rev. **467**, 214615.
154. Chen, H., Shen, K., Chen, J., et al. (2017). Hollow-ZIF-templated formation of a ZnO@ C-N-Co core-shell nanostructure for highly efficient pollutant photodegradation. J. Mater. Chem. **5**, 9937–9945.
155. Zhang, Z., Shi, H., Wu, Q., et al. (2019). MOF-derived CeO_2/Au@SiO_2 hollow nanotubes and their catalytic activity toward 4-nitrophenol reduction. New J. Chem. **43**, 4581–4589.
156. Li, S., Hou, Y., Chen, Q., et al. (2019). Promoting active sites in MOF-derived homobimetallic hollow nanocages as a high-performance multifunctional nanozyme catalyst for biosensing and organic pollutant degradation. ACS Appl. Mater. Interfaces **12**, 2581–2590.
157. Khan, M.A.N., Klu, P.K., Wang, C.H.C., et al. (2019). Metal-organic framework-derived hollow Co_3O_4/carbon as efficient catalyst for peroxymonosulfate activation. Chem. Eng. J. **363**, 234–246.
158. Liu, X., Ma, R., Wang, X., et al. (2019). Graphene oxide-based materials for efficient removal of heavy metal ions from aqueous solution: a review. Environ. Pollut. **252**, 62–73.
159. Liu, X., Ma, R., Zhuang, L., et al. (2021). Recent developments of doped g-C_3N_4 photocatalysts for the degradation of organic pollutants. Crit. Rev. Environ. Sci. Technol. **51**, 751–790.
160. Liu, X., Pang, H., Liu, X., et al. (2021). Orderly porous covalent organic frameworks-based materials: superior adsorbents for pollutants removal from aqueous solutions. Innovation **2**, 100076.
161. Pervaiz, E., Virk, M.S.A., Tareen, A.K., et al. (2018). Magnetic micro scavengers: highly porous $Ni_{1-x}Co_xFe_2O_4$ microcubes for efficient disintegration of nitrophenol. Nanotechnology **29**, 215710.
162. Zhao, Y., Shi, J., Wang, X., et al. (2020). Biomass@MOF-derived carbon aerogels with a hierarchically structured surface for treating organic pollutants. Ind. Eng. Chem. Res. **59**, 17529–17536.
163. Shang, Y., Xu, X., Gao, B., et al. (2021). Single-atom catalysis in advanced oxidation processes for environmental remediation. Chem. Soc. Rev. **50**, 5281–5322.
164. Mi, X., Wang, P., Xu, S., et al. (2021). Almost 100% peroxymonosulfate conversion to singlet oxygen on single-atom CoN_{2+2} sites. Angew. Chem. Int. Ed. Engl. **133**, 4638–4643.
165. Yang, H., Liu, X., Hao, M., et al. (2021). Functionalized iron-nitrogen-carbon electrocatalyst provides a reversible electron transfer platform for efficient uranium extraction from seawater. Adv. Mater. **33**, 2106621.
166. Peng, Y., Li, Y., Ban, Y., et al. (2014). Metal-organic framework nanosheets as building blocks for molecular sieving membranes. Science **346**, 1356–1359.
167. Wang, X., Zhang, S., Shao, M., et al. (2017). Fabrication of ZnO/$ZnFe_2O_4$ hollow nanocages through metal organic frameworks route with enhanced gas sensing properties. Sensor. Actuator. B Chem. **251**, 27–33.
168. Li, Z., Zhang, Y., Zhang, H., et al. (2020). Superior NO_2 sensing of MOF-derived indium-doped ZnO porous hollow cages. ACS Appl. Mater. Interfaces **12**, 37489–37498.
169. Koo, W.T., Yu, S., Choi, S.J., et al. (2017). Nanoscale PdO catalyst functionalized Co_3O_4 hollow nanocages using MOF templates for selective detection of acetone molecules in exhaled breath. ACS Appl. Mater. Interfaces **9**, 8201–8210.
170. Cao, E., Guo, Z., Song, G., et al. (2020). MOF-derived $ZnFe_2O_4$/(Fe-ZnO) nanocomposites with enhanced acetone sensing performance. Sensor. Actuator. B Chem. **325**, 128783.
171. Wang, C., Kim, J., Tang, J., et al. (2020). Large-scale synthesis of MOF-derived superporous carbon aerogels with extraordinary adsorption capacity for organic solvents. Angew. Chem. Int. Ed. Engl. **59**, 2066–2070.
172. Chen, J., Lv, H., Bai, X., et al. (2021). Synthesis of hierarchically porous Co_3O_4/Biomass carbon composites derived from MOFs and their highly NO_2 gas sensing performance. Microporous Mesoporous Mater. **321**, 111108.
173. Garg, N., Deep, A., and Sharma, A.L. (2021). Metal-organic frameworks based nanostructure platforms for chemo-resistive sensing of gases. Coord. Chem. Rev. **445**, 214073.
174. Liu, X., Xie, Y., Chen, Z., et al. (2022). Highly efficient electrocatalytic uranium extraction from seawater over a flexible amidoxime—functionalized In—N—C catalyst. Adv. Sci. **9**, 2201735.
175. Zhao, Z., Zhang, Z., Li, C., et al. (2018). MOF derived iron oxide-based smart plasmonic Ag/Au hollow and porous nanoshells "ultra-microelectrodes" for ultra-sensitive detection of arsenic. J. Mater. Chem. **6**, 16164–16169.
176. Ma, B., Guan, P.Y., Li, Q.Y., et al. (2016). MOF-derived flower-like MoS_2@TiO_2 nanohybrids with enhanced activity for hydrogen evolution. ACS Appl. Mater. Interfaces **8**, 26794–26800.
177. Zhao, Y., Zheng, L., Jiang, D., et al. (2021). Nanoengineering metal-organic framework-based materials for use in electrochemical CO_2 reduction reactions. Small **17**, 2006590.
178. Li, Y., Adli, N.M., Shan, W., et al. (2022). Atomically dispersed single Ni site catalysts for high-efficiency CO_2 electroreduction at industrial-level current densities. Energy Environ. Sci. **15**, 2108–2119.
179. Wang, Z., Zhou, Y., Liu, D., et al. (2022). Carbon-confined indium oxides for efficient carbon dioxide reduction in a solid-state electrolyte flow cell. Angew. Chem. Int. Ed. Engl. e202200552. https://doi.org/10.1002/ange.2022.

ACKNOWLEDGMENTS

Financial support from the National Key Research and Development Program of China (2018YFC1900105), NSFC (22006036; U2167218), and Beijing Outstanding Young Scientist Program is greatly appreciated.

AUTHOR CONTRIBUTIONS

X.L. wrote the entire manuscript. G.V., Z.C., B.H., and Q.H. polished the article. H.Y., S.M., and X.W. revised and improved the entire article.

DECLARATION OF INTERESTS

The authors declare no competing interests.

SUPPLEMENTAL INFORMATION

Supplemental information can be found online at https://doi.org/10.1016/j.xinn.2022.100281.

LEAD CONTACT WEBSITE

http://www.craes.cn/zjhky/craes_kydw/yjy/201808/t20180819_454143.shtml
http://life.usx.edu.cn/info/1010/1383.htm
https://cese.ncepu.edu.cn/szdw/6e9a3bd804ff41e39fdbe63fc9f8f2ed.htm.

The Innovation, Volume *3*

Supplemental Information

Metal-organic framework nanocrystal-derived hollow porous materials: Synthetic strategies and emerging applications

Xiaolu Liu, Gaurav Verma, Zhongshan Chen, Baowei Hu, Qifei Huang, Hui Yang, Shengqian Ma, and Xiangke Wang

Supplemental Information on

Metal-Organic Framework Nanocrystals Derived Hollow Porous Materials: Synthetic Strategies and Emerging Applications

Xiaolu Liu[a,b], Gaurav Verma[c], Zhongshan Chen[a], Baowei Hu[b], Qifei Huang[d], Hui Yang,[a,*] Shengqian Ma[c,*] Xiangke Wang,[a,b*]

[a] College of Environmental Science and Engineering, North China Electric Power University, Beijing, 102206, P. R. China. E-mail: h.yang@ncepu.edu.cn; xkwang@ncepu.edu.cn

[b] School of Life Science, Shaoxing University, Huancheng West Road 508, Shaoxing 312000, P.R. China

[c] Department of Chemistry, University of North Texas, 1508 W Mulberry St, Denton, TX 76201, USA. E-mail: shengqian.ma@unt.edu

[d] State Key Laboratory of Environmental Criteria and Risk Assessment, Chinese Research Academy of Environmental Sciences, Beijing 100012, P.R. China

Contents:

Supplementary Figures: 5
Supplementary Tables: 4
References for SI reference citations

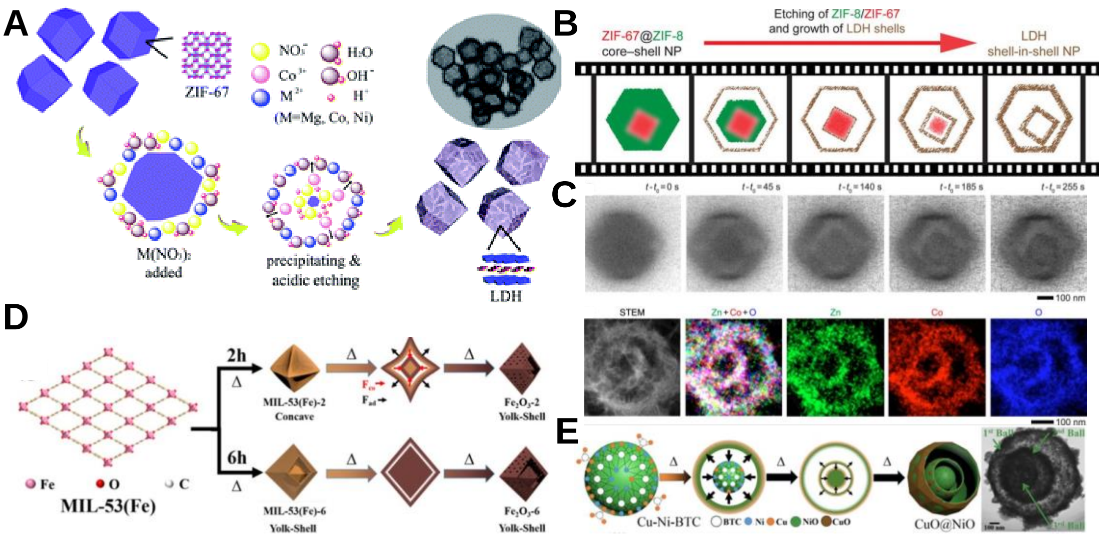

Figure S1. (A) The formation illustration of LDH nanocages by simultaneous precipitation and acidic etching.[1] (B) Schematic illustration depicting the conversion process of ZIF-67@ZIF-8 core-shell NPs into "shell-in-shell" LDH nanocages. (C) (top) Time series of in situ liquid-phase TEM images showing the room-temperature conversion of a ZIF-67@ZIF-8 NP into a shell-in-shell LDH nanocage, (bottom) STEM image and the corresponding EDX maps of the shell-in-shell LDH nanocage.[2] (D) The formation process of hollow Fe_2O_3 nanostructures with the octahedron and yolk-shell octahedron.[3] (E) Schematic illustration of strategies for using MOF nanocrystals as sacrificial templates and precursors for the synthesis of multilayer CuO@NiO hollow spheres.[4] Copyright Royal Society of Chemistry and American Chemical Society.

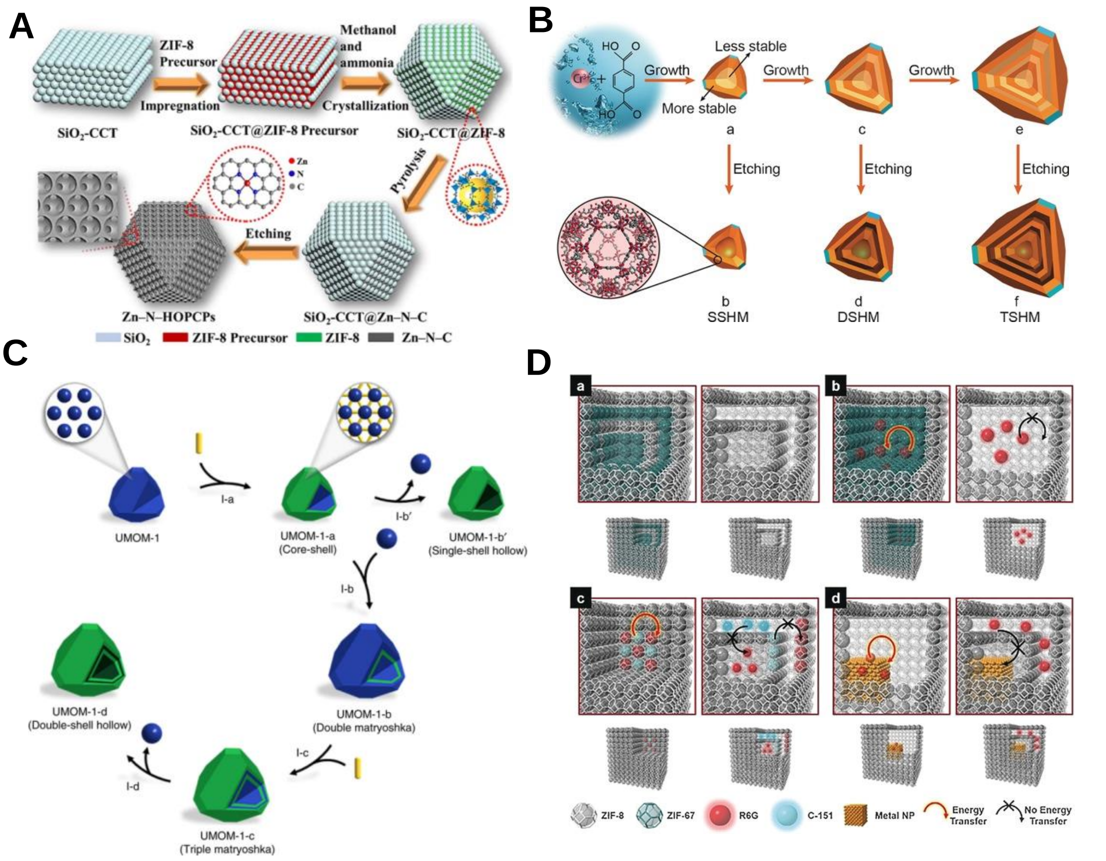

Figure S2. (A) Illustration of the fabrication of the Zn-N-HOPCPs.[5] (B) Fabrication of single-, double-, and triple-shelled hollow MIL-101.[6] (C) Schematic showing the synthetic process of single-crystal hollow MOFs with multishell via an etching-epitaxial growth strategy.[7] (D) Fabrication of multishelled hollow ZIF-8 by using multilayered ZIF-67@ZIF-8 microcrystals as templates.[8] Copyright Elsevier, John Wiley and Sons, and Springer Nature.

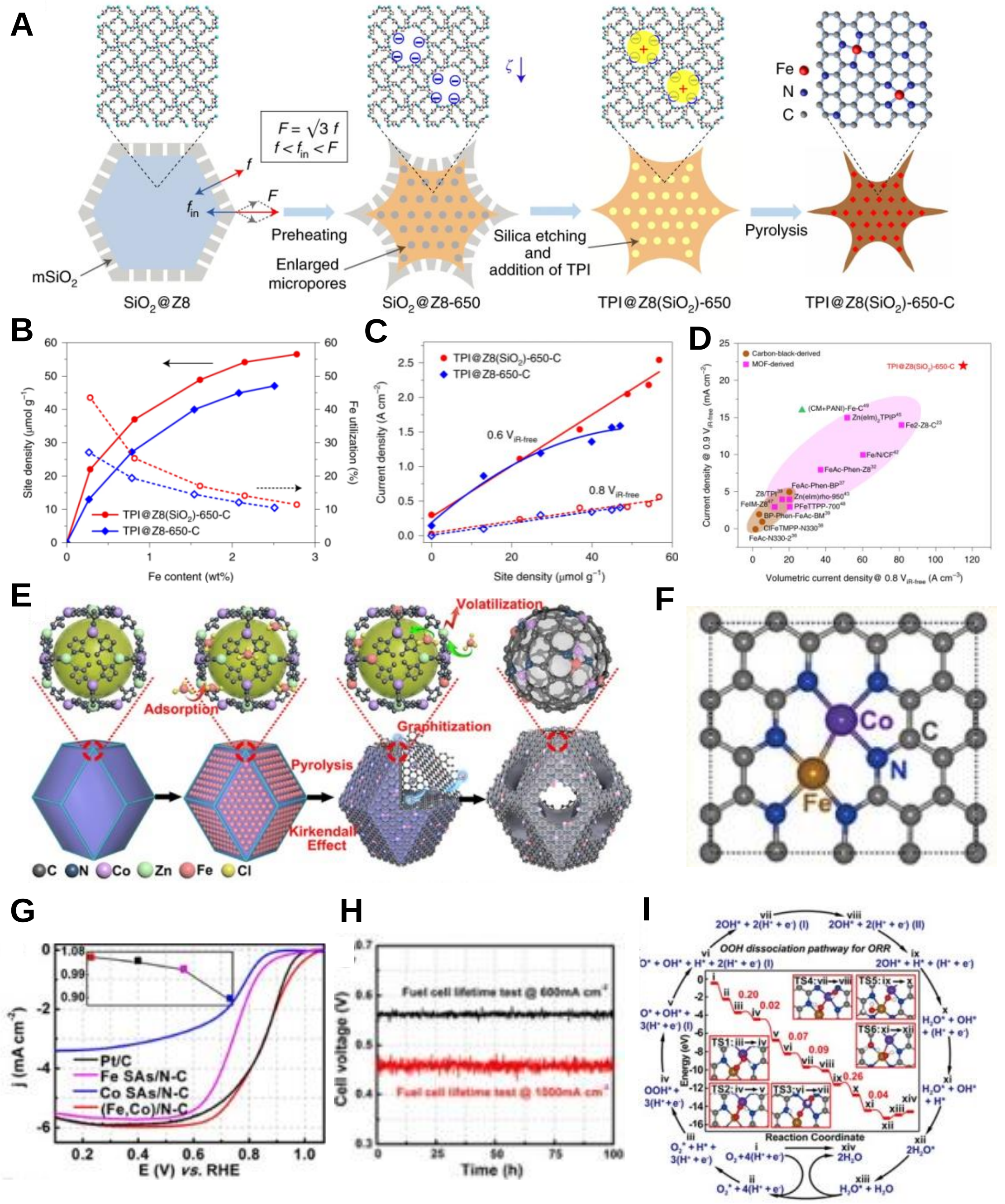

Figure S3. MOF-derived hollow porous materials and their application in ORR. (A) Schematic synthesis process of TPI@Z8(SiO_2)-650-C. (B) The SD and Fe utilization of indicated catalysts as a function of Fe content. (C) The PEMFC current density as a function of SD of indicated Fe-N-C catalysts (voltages of 0.8 and 0.6 $V_{iR\text{-free}}$, test conditions: 80 °C, 100% RH, 2.5 bar H_2-O_2). (D) PEMFC activity comparison between TPI@Z8(SiO_2)-650-C and the literature.[9] (E) Preparation of (Fe,Co)/N-C and (F) proposed architectures of Fe-Co dual sites. (G) RDE polarization curves of (Fe,Co)/N-C and reference sample in O_2-saturated 0.1 M $HClO_4$ with sweep rate 10 mV s^{-1} and rotation rate 1600 rpm. (H) Stability of (Fe,Co)/N-C in a H_2/air fuel cell measured at 600 mA cm^{-2}

and 1000 mA cm^{-2}. (I) Energies of intermediates and transition states in mechanism of ORR at (Fe,Co)/N-C from DFT.[10] Copyright Spring Nature and American Chemical Society.

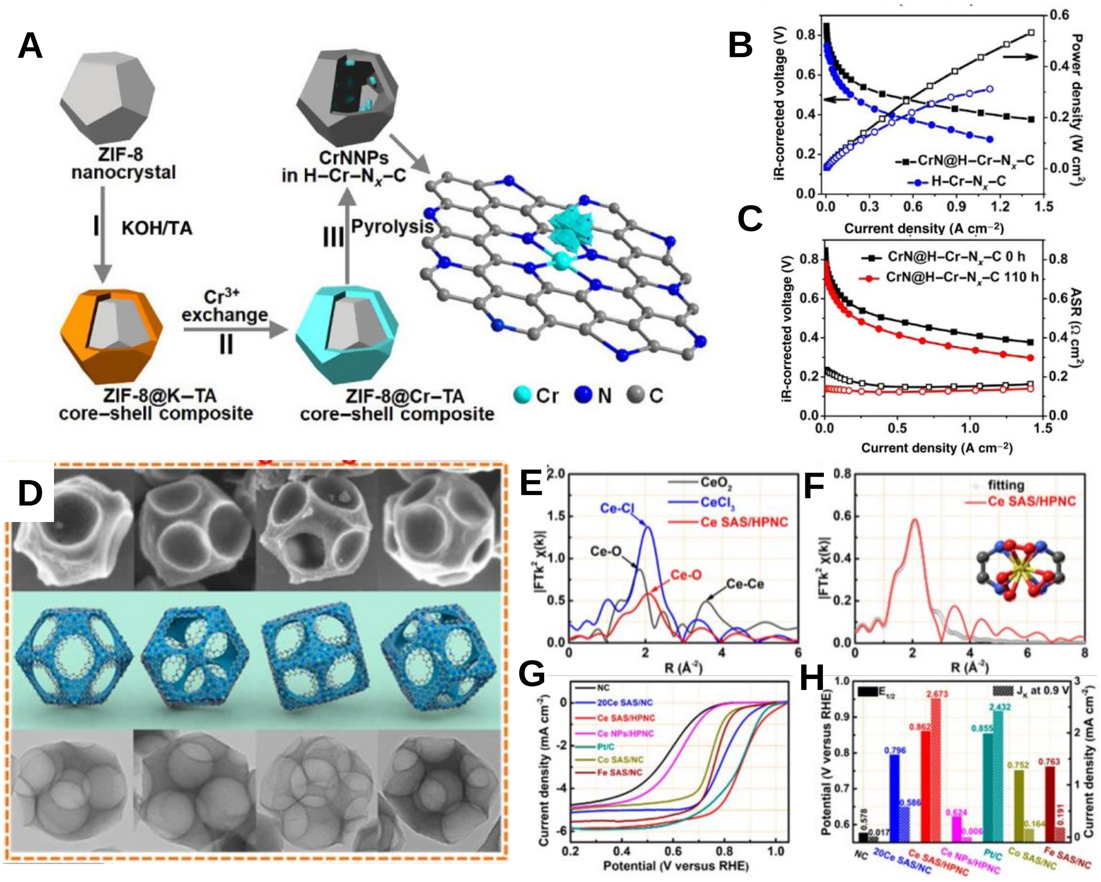

Figure S4. MOF-derived hollow porous materials and their application in ORR. (A) Synthetic scheme for the preparation of CrN@H-Cr-N_x-C. (B) I-V polarization and power density curves for H_2-O_2 PEMFC and (C) fuel cell durability tests of catalysts.[11] (D) Images of Ce SAS/HPNC corresponding to the models from different angles. (E) FT-EXAFS spectra of Ce SAS/HPNC, and reference sample at the Ce L_3-edge. (F) FT-EXAFS fitting curve of the sample and proposed architectures of Ce-N_4/O_6. (G) ORR performance of Ce SAS/HPNC and reference samples (electrolyte 0.1 M $HClO_4$, sweep rate 10 mV s^{-1}, rotating rate 1600 rpm). (H) Comparison of $E_{1/2}$ and J_K of different catalysts.[12] Copyright CCS Chemistry and American Chemical Society.

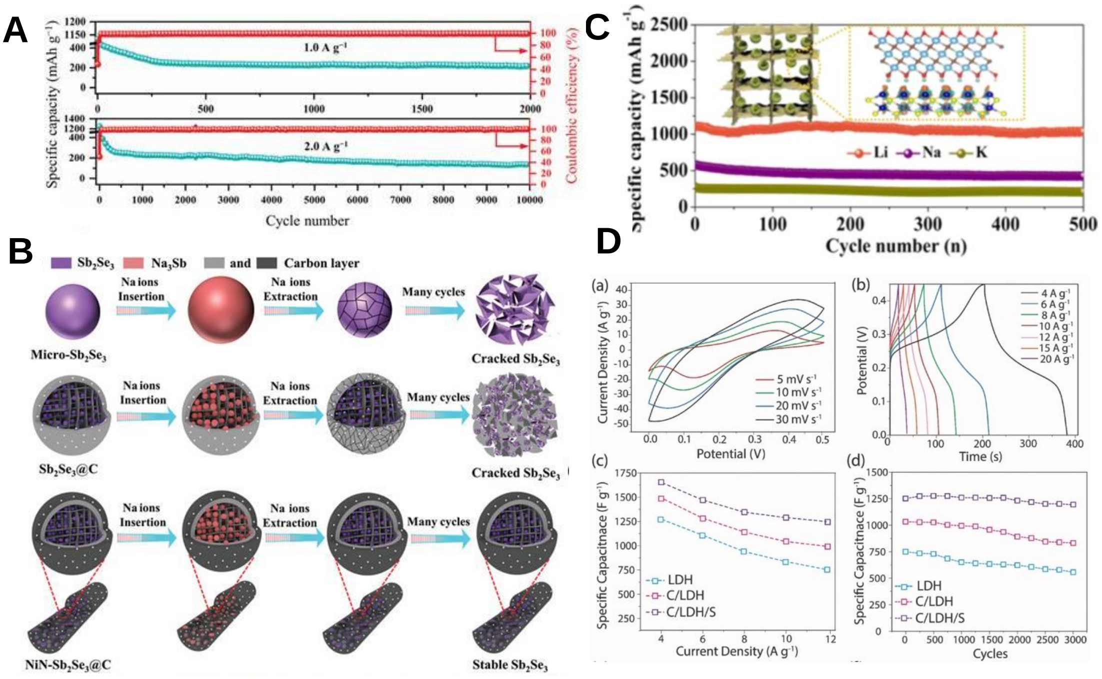

Figure S5. MOF-derived hollow porous materials and their application energy storage and conversions. (A) Cycling performance of NiN-Sb_2Se_3@C electrodes. (B) Schematic illustration of the structure evolution of different electrodes.[13] (C) The long-term cycling stability of (CoS NP@NHC)@MXene for LIBs, SIBs), and PIBs.[14] (D) Supercapacitive performance of NiCo-LDH/Co9S8, (a) CV curves at various scan rates, (b) charge-discharge profiles at various current densities, (c)specific capacitances as a function of current density, (d) cycling stability tests over 3000 cycles.[15] Copyright American Chemical Society, and John Wiley & Sons.

Table S1 A summary of synthetic strategies of MOFs- derived hollow porous materials.

Materials type	Derived from	Preparation strategy	Ref.
Yolk-shell $ZnO@SiO_2$	Core-shell ZIF-8@SiO_2	ZIF-8 as removable templates	16
Yolk–shell ZIF-8@SiO_2	Core-shell ZIF-8@SiO_2	ZIF-8 as removable templates	16
Hollow SiO_2	Core-shell ZIF-8@SiO_2	ZIF-8 as removable templates	16
Hollowparticle	ZIF@shell composite material	ZIF-8 as sacrificial templates	17
Hollow composite nanostructures	ZIF@shell composite material	ZIF-8 as sacrificial templates	17
Hollow ZIF/NP yolk@shell material	ZIF@shell composite material	ZIF-8 as sacrificial templates	17
Mg-Co LDH nanocages.	ZIF-67(Co)	ZIF-67 as both self-sacrificing templates and precursors	18
Ni-Co LDH nanocages	ZIF-67(Co)	ZIF-67 as both self-sacrificing templates and precursors	18
NiCoMn-OH	ZIF-67(Co)	ZIF-67 as both self-sacrificing templates and precursors	19
$FeOOH@Ni(OH)_2$	MIL-53(Fe)	MIL-53 as both self-sacrificing templates and precursors	20
Co^{2+}-Co^{3+} LDH	ZIF-67(Co)	ZIF-67 as both self-sacrificing templates and precursors	21
Ni-Fe LDH nanocages	MIL-88A	MIL-88A as both self-sacrificing templates and precursors	22
Hollow Co_3O_4 tetrahedra	Soild Co-based MOFs	MOFs as both self-sacrificing templates and precursors	23
Hollow Fe_2O_3 nanostructures	MIL-53 (Fe)	MIL-53 as both self-sacrificing templates and precursors	24
Multilayer CuO@NiO	Ni-Co-BTC MOF	Ni-Co-BTC MOF as both self-sacrificing templates and precursors	25
ZIF-67/Ni-Co LDH yolk-shell structure	ZIF-67(Co)	ZIF-67 as both self-sacrificing templates and precursors	26
$Co_3O_4@NiCo_2O_4$ core-shell nanocages	ZIF-67(Co)	ZIF-67 as both self-sacrificing templates and precursors	26
$Ni_xCo_{3-x}O_{4-y}$ nanocage	ZIF-67(Co)	ZIF-67 as both self-sacrificing templates and precursors	27
Hollow CuO-$CuCo_2O_4$ dodecahedrons	ZIF-67(Co)	ZIF-67 as both self-sacrificing templates and precursors	28
$ZnO/ZnCo_2O_4$ core-shell nanocages	ZIF-8	ZIF-8 as both self-sacrificing templates and precursors	29
Hollow NiO_x/Co_3O_4	ZIF-67(Co)	ZIF-67 as both self-sacrificing templates and precursors	30
Hollow carbon matrix	ZIF-8	ZIF-8 as both self-sacrificing templates and precursors	31
NPS-HCS	ZIF-8@PZS	ZIF-8@PZS composites as precursors	32

NC@Co-NGC nanocage	ZIF-8@ZIF-67 core-shell	ZIF-8@ZIF-67 core-shell composites as precursors	33
NiO_x/Ni@C composites	Ni-MOFs	Ni-MOFs composites as precursors	34
Hierarchical CoS_2 hollow prisms	ZIF-67 hollow prisms	ZIF-67 hollow prisms as precursors	35
Zn/Ni-MOF-2 hollow nanocubes	Zn/Ni-MOF-5	Zn/Ni-MOF-5 composites as precursors	36
3D hollow SOM-ZIF-8	ZIF-8@PSs	ZIF-8@PSs composites as precursors	37

Table S2 Summary of OER and HER performance of MOFs derived hollow porous electrocatalysts.

Materials	Application	Performance	Electrolyte	Ref.
Co/CoS/Fe-HSNC	OER	J=10 mA cm^{-2}, η=250 mV	1.0 M KOH	38
FeS_2–CoS_2/NCFs	OER	1.57 V at 10 mA cm^{-2}	0.1 M KOH	39
FeNiCo@NC-P	OER	1.54 V at 10 mA cm^{-2}	0.1 M KOH	40
Co-CNTs/NS-$CoO_x$$AlO_y$	OER	J=10 mA cm^{-2}, η=280 mV	0.1 M KOH	41
CoNCNTF/CNFs	OER	1.61 V at 10 mA cm^{-2}	0.1 M KOH	42
Co/Co_3O_4@PGS	OER	1.58 V at 10 mA cm^{-2}	0.1 M KOH	43
CoNi-SAs/NC	OER	J=10 mA cm^{-2}, η=340 mV	in alkaline media	44
Co@hNCTs-800	OER	1.63 V at 10 mA cm^{-2}	0.1 M KOH	45
Capsular-MOF	OER	1.59 V at 10 mA·cm^{-2}	1.0 M KOH	46
Co_3O_4/HNCP-40	OER	J=10 mA cm^{-2}, η=333 mV	1.0 M KOH	47
NC@GC	OER	1.59 V at 10 mA cm^{-2}	1.0 M KOH	48
		J=10 mA cm^{-2}, η=132 mV	1.0 M KOH	
Ni-CoP/HPFs	HER	J=10 mA cm−2, η=144 mV	0.5 M H_2SO_4	49
		J=10 mA cm−2, η=92 mV	1.0 M KOH	

Table S3 Summary of MOFs derived hollow porous materials for supercapacitor applications.

Materials	Capacity (F g^{-1})	Current density (A g^{-1})	scan rates (mV s^{-1})	BET/cm g^{-1}	Ref.
Co_9S_8@NiO	1627	1.0	/	42.62	50
NiCoMn-S	2098.2	1.0	/	94.9	51
CoS-NP/CoS-NS DSNBs	980	1.0	/	110	52
$Ti_3C_2T_X$/ZIF67/CoV_2O_6	253.8	5.0	100	/	53
NPC530	158	0.05	/	3040	54
MC-A	208	/	2	1674	55
Z-900	257	/	5	1075	56
AS-ZC-800	211	/	10	1972	57
NPC	226	/	5	1523	58
NC@GC	216	2	/	1276	59
MOF-DC	238	0.15	/	2714	60
Co-MOF	103	0.6	/	2900	61
UIO-66(Zr)	104	/	5	596	62
Ni-BDC	395	1.4		117	63
Ni-BDC-24	394	0.5	/	/	64

Co-BDC derived Co_3O_4	69	0.5	/	47.12	65
Fe-MIL-88B-NH_2 derived Fe_3O_4	139	0.5	/	37.7	66
MIL-101 (Cr) derived Cr_2O_3	180	/	2	438	67
Ce-BTC derived CeO_2	201	0.2	/	77	68
MOF-199 derived copper oxide	300	/	2	/	69
ZIF-67 derived hollow Co_3O_4	440	1.25	/	128	70
MOF-Ni-CoP@C@CNT	425	1	/	97	71
$Co(VO_3)_2$ $Co(OH)_2$	402	0.5	/	/	72
CC@$NiCo_2O_4$	633	2.5	/	11.6	73
MOF-Co_9S_8	1252	1	/	/	74
MOF-NiS	744	1	/	/	75
Ni–Zn–Co–S NSAs	1289	/	10	/	76
Co_3O_4-C/$Ni_2P_2O_7$	1142	1	/	/	77
C-ZIF-8	100	/	20	925	78

Table S4 Abbreviations

Abbreviation	Full title
BPA	Bisphenol A
BTC	Benzene-1,3,5-tricarboxylate
CCT	Colloidal crystal template
CD	Charge-discharge
CdI	Couble-layer capacitance
CHE	Computational hydrogen electrode
CM	Carbon matrix
CNTs	Carbon nanotubes
CO_2RR	CO_2 reduction reactions
DABCO	1,4-diazabicyclo [2.2.2] octane, as a linker
DFT	Density functional theory
DOE	Department of Energy
DPGG	1,2-dipalmitoyl-sn-glycero-3-galloyl
$E_{1/2}$	Positive half-wave potential
E_{onset}	Onset potential
FT-EXAFS	Fouriertransformed extended X-ray absorption fine structure
FESEM	Field emission scanning electron microscopy
HER	Hydrogen evolution reaction
HMF	5-hydroxymethylfurfural
HPC	Hollow porous carbons
HPMs	Hollow porous materials
J_k	Kinetic current density
K-TA	Polymer potassium tannate
LDH	Layered Double Hydroxide
LIBs	Lithium-ion batteries
LiPSs	Lithium polysulfides
MNPs	Metal nanoparticles
MO	Methyl orange
MOFs	Metal-organic frameworks
MOP	Metal−organic polyhedral
NCNTFs	N-doped carbon nanotube frameworks
NHPC	Hollow nitrogen-doped porous carbon
NPs	Nanoparticles
NPS-HCS	N, P, S-doped carbon shell
OCV	Open-circuit voltage
OER	Oxygen evolution reaction
ORR	Oxygen reduction reaction
PCPs	Porous coordination polymers
PIBs	Potassium-ion batteries
PMPS	Peroxymonosulfate
PZS	Cyclotriphosphazene-co-4,4’-sulfonyldiphenol
PS	Polystyrene microsphere

RH	Relative humidity
RhB	Rhodamine B
SIBs	Sodium-ion batteries
TEM	Transmission electron microscopy
TEOS	Tetraethylorthosilicate
ZABs	Zn-air batteries
ZIF-8@Cr-TA	ZIF-8@chromium-tannic acid

References

[1] Jiang, Z., Li, Z.P., Qin, Z.H., et al. (2013). LDH nanocages synthesized with MOF templates and their high performance as supercapacitors. Nanoscale ***5***, 11770-11775.

[2] Wang, W., Yan, H., Anand, U., et al. (2021). Visualizing the conversion of metal-organic framework nanoparticles into hollow layered double hydroxide nanocages. J. Am. Chem. Soc. ***143***, 1854-1862.

[3] Guo, W., Sun, W., Lv, L.P., et al. (2017). Microwave-assisted morphology evolution of Fe-based metal-organic frameworks and their derived Fe_2O_3 nanostructures for Li-ion storage. ACS Nano ***11***, 4198-4205.

[4] Guo, W., Sun, W., Wang, Y. (2015). Multilayer CuO@NiO hollow spheres: microwave-assisted metal-organic-framework derivation and highly reversible structure-matched stepwise lithium storage. ACS Nano ***9***, 11462-11471.

[5] Guo, Y., Feng, L., Wu, C., et al. (2020). Confined pyrolysis transformation of ZIF-8 to hierarchically ordered porous Zn-NC nanoreactor for efficient CO_2 photoconversion under mild conditions. J. Catal. ***390***, 213-223.

[6] Liu, W., Huang, J., Yang, Q., et al. (2017). Multi-shelled hollow metal-organic frameworks. Angew. Chem. Int. Edit. ***56***, 5512-5516.

[7] Lee, J., Kwak, J. H., Choe, W. (2017). Evolution of form in metal-organic frameworks. Nat. Commu. ***8***, 1-8.

[8] Liu, X.Y., Zhang, F., Goh T.W., et al. (2018). Using a multi-shelled hollow metal-organic framework as a host to switch the guest-to-host and guest-to-guest interactions. Angew. Chem. Int. Ed. ***57***, 2110-2114.

[9] Wan, X., Liu, X., Li, Y., et al. (2019). Fe-N-C electrocatalyst with dense active sites and efficient mass transport for high-performance proton exchange membrane fuel cells. Nat. Catal. ***2***, 259-268.

[10] Wang, J., Huang, Z., Liu, W., et al. (2017). Design of N-coordinated dual-metal sites: a stable and active Pt-free catalyst for acidic oxygen reduction reaction. J. Am. Chem. Soc. ***139***, 17281-17284.

[11] Yang, H., Wang, X., Zheng, T., et al. (2021). CrN-encapsulated hollow Cr-NC capsules boosting oxygen reduction catalysis in PEMFC. CCS Chemistry ***3***, 208-218.

[12] Zhu, M., Zhao, C., Liu, X., et al. (2021). Single atomic cerium sites with a high coordination number for efficient oxygen reduction in proton-exchange membrane fuel cells. ACS Catal. ***11***, 3923-3929.

[13] Li, Q., Zhang, W., Peng, J., et al. (2022). Nanodot-in-nanofiber structured carbon-confined Sb_2Se_3 crystallites for fast and durable sodium storage. Adv. Funct. Mater. 2112776.

[14] Yao, L., Gu, Q.M., Yu, X.B. (2021). Three-dimensional MOFs@MXene aerogel composite derived MXene threaded hollow carbon confined CoS nanoparticles toward advanced alkali-ion batteries. ACS Nano ***15***, 3228-3240.

[15] Yilmaz, G., Yam, K.M., Zhang, C., et al. (2017). In situ transformation of MOFs into layered double hydroxide embedded metal sulfides for improved electrocatalytic and supercapacitive performance. Adv. Mater. ***29***, 1606814.

[16] He, L., Li, L., L. Zhang, L.Y., et al. (2014) ZIF-8 templated fabrication of rhombic dodecahedron-shaped ZnO@SiO_2, ZIF-8@SiO_2 yolk-shell and SiO_2 hollow nanoparticles. Crystengcomm ***16***, 6534-6537.

[17] Yang, H., Kruger, P.E., Telfer, S.G., et al. (2015). Metal-organic framework nanocrystals as sacrificial templates for hollow and exceptionally porous titania and composite materials. Inorg. Chem. ***54***, 9483-9490.

[18] Jiang, Z., Li, Z.P., Qin, Z.H., et al. (2013). LDH nanocages synthesized with MOF templates and their high performance as supercapacitors. Nanoscale ***5***, 11770-11775.

[19] Du, Y.Q., Li, G.Y., Chen, M.D., et al. (2019). Hollow nickel-cobalt-manganese hydroxide polyhedra via MOF templates for high-performance quasi-solid-state supercapacitor. Chem. Eng. J. ***378***, 122210.

[20] Wang, J., Li, S., Lin, R., et al. (2019). MOF-derived hollow β-FeOOH polyhedra anchored with α-Ni(OH)2 nanosheets as efficient electrocatalysts for oxygen evolution. Electrochim Acta. ***301***, 258-266.

[21] Bai, X., Liu, J., Liu, Q., et al. (2017). In-situ fabrication of MOF-derived Co-Co layered double hydroxide hollow nanocages/graphene composite: a novel electrode material with superior electrochemical performance. Chemistry A European Journal ***23***, 14839-14847.

[22] Zhang, J., Yu, L., Chen, Y., et al. (2020). Designed formation of double-shelled Ni-Fe layered-double-hydroxide nanocages for efficient oxygen evolution reaction. Adv. Mater. ***32***, 1906432.

[23] Tian, D., Zhou, X.L., Zhang, Y.H., et al. (2015). MOF-derived porous Co_3O_4 hollow tetrahedra with excellent performance as anode materials for lithium-ion batteries. Inorg. Chem. ***54***, 8159-8161.

[24] Guo, W., Sun, W., Lv, L.P., et al. (2017). Microwave-assisted morphology evolution of Fe-based metal-organic frameworks and their derived Fe_2O_3 nanostructures for Li-ion storage. ACS Nano ***11***, 4198-4205.

[25] Guo, W., Sun, W., Wang, Y. (2015). Multilayer CuO@NiO hollow spheres: microwave-assisted metal-organic-framework derivation and highly reversible structure-matched stepwise lithium storage. ACS Nano ***9***, 11462-11471.

[26] Hu, H., Guan, B., Xia, B., et al. (2015). Designed formation of Co_3O_4/$NiCo_2O_4$ double-shelled nanocages with enhanced pseudocapacitive and electrocatalytic properties. J. Am. Chem. Soc. ***137***, 5590-5595.

[27] Antony, R.P., Satpati, A.K., Bhattacharyya, K., et al. (2016). MOF derived nonstoichiometric $Ni_xCo_{3-x}O_{4-y}$ nanocage for superior electrocatalytic oxygen evolution. Adv. Mater. Interfaces, 3, 1600632.

[28] Zhen, S.Y., Wu, H.T., Wang, Y., et al. (2019). Metal-organic framework derived hollow porous CuO-$CuCo_2O_4$ dodecahedrons as a cathode catalyst for LiO_2 batteries. RSC Adv. 9, 16288-16295.

[29] Qu, F., Jiang, H., Yang, M. (2016). Designed formation through a metal organic framework route of ZnO/$ZnCo_2O_4$ hollow core-shell nanocages with enhanced gas sensing properties. Nanoscale ***8***, 16349-16356.

[30] Chen, X., Li, J.J., Chen, X., et al. (2018). MOF-templated approach for hollow NiO_x/Co_3O_4 catalysts: enhanced light-driven thermocatalytic degradation of toluene. ACS Appl. Nano. Mater. ***1***, 2971-2981.

[31] Zhang, W., Jiang, X., Zhao, Y., et al. (2017). Hollow carbon nanobubbles: monocrystalline MOF nanobubbles and their pyrolysis. Chem. Sci. ***8***, 3538-3546.

[32] Yang, S., Peng, L., Huang, P., et al. (2016). Nitrogen, phosphorus, and sulfur Co-doped hollow

carbon shell as superior metal-free catalyst for selective oxidation of aromatic alkanes. Angew. Chem. ***128***, 4084-4088.

[33] Liu, S., Wang, Z., Zhou, S., et al. (2017). Metal-organic-framework-derived hybrid carbon nanocages as a bifunctional electrocatalyst for oxygen reduction and evolution. Adv. Mater. ***29***, 1700874.

[34] Lv, Z., Fan, Q., Xie, Y., et al. (2019). MOFs-derived magnetic chestnut shell-like hollow sphere NiO/Ni@C composites and their removal performance for arsenic(V). Chem. Eng. J. ***362***, 413-421.

[35] Yu, L., Yang, J.F., Lou, X.W. (2016). Formation of CoS2 nanobubble hollow prisms for highly reversible lithium storage. Angew. Chem. Int. Edit. ***128***, 13620-13624.

[36] Zhang, Z., Chen, Y., He, S., et al. (2014). Hierarchical Zn/Ni-MOF-2 nanosheet-assembled hollow nanocubes for multicomponent catalytic reactions. Angew. Chem. Int. Edit. ***53***, 12517-12521.

[37] Shen, K., Zhang, L., Chen, X., et al. (2018). Ordered macro-microporous metal-organic framework single crystals. Science ***359***, 206-210.

[38] Yan, L., Wang, H., Shen, J., et al. (2021). Formation of mesoporous Co/CoS/Metal-NC@S, N-codoped hairy carbon polyhedrons as an efficient trifunctional electrocatalyst for Zn-air batteries and water splitting. Chem. Eng. J. ***403***, 126385.

[39] Shi, X., He, B., Zhao, L., et al. (2021). FeS_2-CoS_2 incorporated into nitrogen-doped carbon nanofibers to boost oxygen electrocatalysis for durable rechargeable Zn-air batteries. J. Power Sources ***482***, 228955.

[40] Ren, D., Ying, J., Xiao, M., et al. (2020). Hierarchically porous multimetal-based carbon nanorod hybrid as an efficient oxygen catalyst for rechargeable zinc-air batteries. Adv. Funct. Mater. ***30***, 1908167.

[41] Najam, T., Shah, S.S.A., Ding, W., et al. (2019). Enhancing by nano-engineering: Hierarchical architectures as oxygen reduction/evolution reactions for zinc-air batteries. J. of Power Sources ***438***, 226919.

[42] Ji, D.X., L. F., Li, L.L., et al. (2019). Hierarchical catalytic electrodes of cobalt-embedded carbon nanotube/carbon flakes arrays for flexible solid-state zinc-air batteries. Carbon ***142***, 379-387.

[43] Jiang, Y., Deng, Y.P., Fu, J., et al. (2018). Interpenetrating triphase cobalt-based nanocomposites as efficient bifunctional oxygen electrocatalysts for long-lasting rechargeable zn-air batteries. Adv. Energy Mater. ***8***, 1702900.

[44] Han, X., Ling, X., Yu, D., et al. (2019). Atomically dispersed binary Co-Ni sites in nitrogen-doped hollow carbon nanocubes for reversible oxygen reduction and evolution. Adv. Mater. ***31***, 1905622.

[45] Zhou, Q., Zhang, Z., Cai, J., et al. (2020). Template-guided synthesis of Co nanoparticles embedded in hollow nitrogen doped carbon tubes as a highly efficient catalyst for rechargeable Zn-air batteries. Nano Energy ***71***, 104592.

[46] Wei, Y.S., Zhang, M., Kitta, M., et al. (2019). A single-crystal open-capsule metal-organic framework. J. Am. Chem. Soc. ***141***, 7906-7916.

[47] Ding, D., Shen, K., Chen, X., et al. (2018). Multi-level architecture optimization of MOF-templated Co-based nanoparticles embedded in hollow N-doped carbon polyhedra for efficient OER and ORR. ACS Catal. 8, 7879-7888.

[48] Wang, Z., Lu, Y., Yan, Y., et al. (2016). Core-shell carbon materials derived from metal-organic frameworks as an efficient oxygen bifunctional electrocatalyst. Nano Energy ***30***, 368-378.

[49] Pan, Y., Sun, K., Lin, Y., et al. (2019). Electronic structure and d-band center control engineering over M-doped CoP (M= Ni, Mn, Fe) hollow polyhedron frames for boosting hydrogen production. Nano energy ***56***, 411-419.

[50] Wang, J., Huang, Y., Han, X., et al. (2021). Construction of hierarchical Co_9S_8@NiO synergistic microstructure for high-performance asymmetric supercapacitor. J. Colloid. Interf. Sci. ***603***, 440-449.

[51] Kang, C., Ma, L., Chen, Y., et al. (2022). Metal-organic framework derived hollow rod-like NiCoMn ternary metal sulfide for high-performance asymmetric supercapacitors. Chem. Eng. J. ***427***, 131003.

[52] Hu, H., Guan, B.Y., Lou, X.W. (2016). Construction of complex CoS hollow structures with enhanced electrochemical properties for hybrid supercapacitors. Chem ***1***, 102-113.

[53] Liu, C., Bai, Y., Li, W., et al. (2022). In situ growth of three-dimensional MXene/Metal-organic framework composites for high-performance supercapacitors. Angew. Chem. Int. Edit., ***134***, e202116282.

[54] Liu, B., Shioyama, H., Jiang, H., et al. (2010). Metal-organic framework (MOF) as a template for syntheses of nanoporous carbons as electrode materials for supercapacitor. Carbon ***48***, 456-463.

[55] Hu, J., Wang, H., Gao, Q., et al. (2010). Porous carbons prepared by using metal-organic framework as the precursor for supercapacitors. Carbon ***48***, 3599-3606.

[56] Chaikittisilp, W., Hu, M., Wang, H., et al. (2012). Nanoporous carbons through direct carbonization of a zeolitic imidazolate framework for supercapacitor electrodes. Chem.

Commun. ***48***, 7259-7261.

[57] Amali, A.J., Sun, J.K., Xu, Q. (2014). From assembled metal-organic framework nanoparticles to hierarchically porous carbon for electrochemical energy storage. Chem. Comm. ***50***, 1519-1522.

[58] Salunkhe, R.R., Kamachi, Y., Torad, N.L., et al. (2014). Fabrication of symmetric supercapacitors based on MOF-derived nanoporous carbons. J. Mater. Chem. A ***2***, 19848-19854.

[59] Tang, J., Salunkhe, R.R., Liu, J., et al. (2015). Thermal conversion of core-shell metal-organic frameworks: a new method for selectively functionalized nanoporous hybrid carbon. J. Am. Chem. Soc. ***137***, 1572-1580.

[60] Banerjee, A., Upadhyay, K. K., Puthusseri, D., et al. (2014). MOF-derived crumpled-sheet-assembled perforated carbon cuboids as highly effective cathode active materials for ultra-high energy density Li-ion hybrid electrochemical capacitors (Li-HECs). Nanoscale ***6***, 4387-4394.

[61] Diaz, R., Orcajo, M.G., Botas, J.A., et al. (2012). Co8-MOF-5 as electrode for supercapacitors. Mater. Lett. ***68***, 126-128.

[62]Tan, Y., Zhang, W., Gao, Y., et al. (2015). Facile synthesis and supercapacitive properties of Zr-metal organic frameworks (UiO-66). RSC Adv. ***5***, 17601-17605.

[63] Yan, Y., Gu, P., Zheng, S., et al. (2016). Facile synthesis of an accordion-like Ni-MOF superstructure for high-performance flexible supercapacitors. J. Mater. Chem. A ***4***, 19078-19085.

[64] Yang, J., Xiong, P., Zheng, C., et al. (2014). Metal-organic frameworks: a new promising class of materials for a high performance supercapacitor electrode. J. Mater. Chem. A ***2***, 16640-16644.

[65] Meng, F., Fang, Z., Li, Z., et al. (2016). Porous Co_3O_4 materials prepared by solid-state thermolysis of novel Co-MOF crystal and their superior storage-energy performances for supercapacitors. J. Mater. Chem. A, ***1***, 1.

[66] Meng, W., Chen, W., Zhao, L., et al. (2014). Porous Fe_3O_4/carbon composite electrode material prepared from metal-organic framework template and effect of temperature on its capacitance. Nano Energy, ***8***, 133-140.

[67] Ullah, S., Khan, I.A., Choucair, M., et al. (2015). A novel Cr_2O_3-carbon composite as a high performance pseudo-capacitor electrode material. Electrochimi. Acta ***171***, 142-149.

[68] Maiti, S., Pramanik, A., Mahanty, S. (2014). Extraordinarily high pseudocapacitance of metal organic framework derived nanostructured cerium oxide. Chem. Commun. ***50***, 11717-11720.

[69] Khan, I.A., Badshah, A., Nadeem, M.A., et al. (2014). A copper based metal-organic framework as single source for the synthesis of electrode materials for high-performance supercapacitors and glucose sensing applications. Int. J. Hydrogen. Energ. ***39***, 19609-19620.

[70] Zhang, Y.Z., Wang, Y., Xie, Y.L., et al. (2014). Porous hollow Co_3O_4 with rhombic dodecahedral structures for high-performance supercapacitors. Nanoscale ***6***, 14354-14359.

[71] Gu, J., Sun, L., Zhang, Y., et al. (2020). MOF-derived Ni-doped CoP@C grown on CNTs for high-performance supercapacitors. Chem. Eng. J. ***385***, 123454.

[72] Zhang, Y., Chen, H., Guan, C., et al. (2018). Energy-saving synthesis of MOF-derived hierarchical and hollow $Co(VO_3)_2$-$Co(OH)_2$ composite leaf arrays for supercapacitor electrode materials. ACS appl. Mater. Inter. ***10***, 18440-18444.

[73] Guan, C., Liu, X., Ren, W., et al. (2017). Rational design of metal-organic framework derived hollow $NiCo_2O_4$ arrays for flexible supercapacitor and electrocatalysis. Adv. Energy Mater. ***7***, 1602391.

[74] Yang, Q., Wang, Q., Long, Y., et al. (2020). In situ formation of Co_9S_8 quantum dots in MOF-derived ternary metal layered double hydroxide nanoarrays for high-performance hybrid supercapacitors. Adv. Energy Mater. ***10***, 1903193.

[75] Qu, C., Zhang, L., Meng, W., et al. (2018). MOF-derived α-NiS nanorods on graphene as an electrode for high-energy-density supercapacitors. J. Mater. Chem. A ***6***, 4003-4012.

[76] Huang, Y., Quan, L., Liu, T., et al. (2018). Construction of MOF-derived hollow Ni-Zn-Co-S nanosword arrays as binder-free electrodes for asymmetric supercapacitors with high energy density. Nanoscale ***10***, 14171-14181.

[77] Zhou, Y., Li, X., Li, J., et al. (2019). MOF-derived Co_3O_4-C/$Ni_2P_2O_7$ electrode material for high performance supercapacitors. Chem. Eng. J. ***378***, 122242.

[78] Tang, J., Salunkhe, R.R., Zhang, H., et al. (2016). Bimetallic metal-organic frameworks for controlled catalytic graphitization of nanoporous carbons. Sci. Rep. ***6***, 30295.
